# Gastrulation-stage gene expression in *Nipbl*^+/−^ mouse embryos foreshadows the development of syndromic birth defects

**DOI:** 10.1126/sciadv.adl4239

**Published:** 2024-03-20

**Authors:** Stephenson Chea, Jesse Kreger, Martha E. Lopez-Burks, Adam L. MacLean, Arthur D. Lander, Anne L. Calof

**Affiliations:** ^1^ Department of Developmental and Cell Biology, School of Biological Sciences, University of California Irvine, Irvine, CA 92697, USA.; ^2^ Center for Complex Biological Systems, University of California Irvine, Irvine, CA 92697, USA.; ^3^ Department of Quantitative and Computational Biology, Dornsife College of Letters, Arts, and Sciences, University of Southern California, Los Angeles, CA 90089, USA.; ^4^ Department of Anatomy and Neurobiology, School of Medicine, University of California Irvine, Irvine, CA 92697, USA.

## Abstract

In animal models, *Nipbl* deficiency phenocopies gene expression changes and birth defects seen in Cornelia de Lange syndrome, the most common cause of which is *Nipbl* haploinsufficiency. Previous studies in *Nipbl^+/−^* mice suggested that heart development is abnormal as soon as cardiogenic tissue is formed. To investigate this, we performed single-cell RNA sequencing on wild-type and *Nipbl^+/−^* mouse embryos at gastrulation and early cardiac crescent stages. *Nipbl^+/−^* embryos had fewer mesoderm cells than wild-type and altered proportions of mesodermal cell subpopulations. These findings were associated with underexpression of genes implicated in driving specific mesodermal lineages. In addition, *Nanog* was found to be overexpressed in all germ layers, and many gene expression changes observed in *Nipbl^+/−^* embryos could be attributed to *Nanog* overexpression. These findings establish a link between *Nipbl* deficiency, *Nanog* overexpression, and gene expression dysregulation/lineage misallocation, which ultimately manifest as birth defects in *Nipbl^+/−^* animals and Cornelia de Lange syndrome.

## Teaser

Gene expression changes during gastrulation of *Nipbl*-deficient mice shed light on early origins of structural birth defects.

## INTRODUCTION

Each year, 1 of every 33 babies in the United States is born with a birth defect (*1*), the most prevalent of which are congenital heart defects (CHDs), neural tube defects, and cleft lip/palate (*2*). Given the major impact that birth defects have on infant mortality and morbidity (*3*, *4*), there is a need to elucidate their origins, but their diversity and sporadic nature pose challenges for identifying causal mechanisms. A promising approach is to study genetic syndromes that present multiple, concurrent defects in various body parts, many of which mirror common isolated birth defects. Studying them might thus provide insights into the causes and development of isolated birth defects.

Cornelia de Lange syndrome (CdLS) affects an estimated 1 in 10,000 to 1 in 30,000 live births (*5*) and is characterized by craniofacial anomalies, delays in growth and maturation, intellectual disability, neurological impairments, and abnormalities of limbs, especially arms and hands, coupled with issues in the visual, auditory, gastrointestinal, genitourinary, and cardiopulmonary systems (*6*).

Most CdLS cases—more than 55%—are caused by heterozygous mutations in the gene *Nipped-B-like* (*NIPBL*) (*7*), named for its homology to the *Drosophila* gene *Nipped-B*. These mutations often produce a nonfunctional protein, suggesting that CdLS arises from haploinsufficiency (*8*). Even a subtle 15% reduction in *NIPBL* gene expression can produce a mild yet recognizable CdLS phenotype (*9*). These observations highlight the importance of precise *NIPBL* gene dosage in human development.

The *NIPBL* gene encodes a universally conserved protein that plays a role in loading cohesin onto chromosomes (*10*). Cohesin, similarly conserved and ubiquitous, is a four-subunit protein complex (encoded by the genes *Smc1a*, *Smc3*, *Rad21*, and either *Stag1* or *Stag2*) essential for chromosome organization and genome stability (*11*). Mutations in the cohesin subunits *SMC1* and *SMC3* account for a small proportion of clinically mild CdLS (~5% and <1%, respectively) (*12**–**14*). In addition, mutations in *HDAC8*, which catalyzes cohesin release from chromatin during mitosis, are found in a distinct subset of patients with CdLS (*15*). Mutations in *RAD21* have also surfaced in individuals exhibiting a CdLS-like phenotype but with substantially milder cognitive impairment (*16*). Collectively, these findings reinforce the idea that impairment of cohesin function contributes to CdLS.

Cohesin was initially identified for its role in sister chromatid cohesion during mitosis (*11*). However, pronounced defects in sister chromatid cohesion or irregularities in mitosis have not been observed in either patients with CdLS or *Nipbl*-haploinsufficient (*Nipbl^+/−^*) mice (*17*, *18*), suggesting that cohesin has additional functions. Studies in CdLS patient cells and *Nipbl*-haploinsufficient animal models indicate that cohesin is involved in transcriptional regulation (*19*). Specifically, *Nipbl* haploinsufficiency leads to alterations in the expression of many hundreds to thousands of genes (*18*). Many of the affected genes are regulated by long-distance enhancers (*20*), aligning with the emerging concept of NIPBL and cohesin as critical determinants in DNA looping (*21*).

Most gene expression changes in *Nipbl*-deficient animals are small, usually less than twofold. Although likely to be inconsequential individually, these small changes can act collectively to produce structural and functional defects. For example, in zebrafish, joint depletion of two developmental genes down-regulated by *nipbl* deficiency produced a CdLS-like phenotype (*22*), suggesting that altered gene expression is the ultimate cause of developmental and physiological abnormalities. Thus, CdLS exemplifies a class of genetic disorders known as “transcriptomopathies” (*23*), in which the cumulative or synergistic effects of minor disturbances to gene expression lead to developmental abnormalities.

Previously, we reported that *Nipbl*-haploinsufficient mice displayed birth defects phenocopying those in CdLS (*18*). These included CHDs (primarily atrial septal defects), in about 30% of *Nipbl^+/−^* mice. Subsequently, we used a conditional *Nipbl* allelic series to investigate the role of *Nipbl* expression in the production of CHDs (*24*). That study showed that *Nipbl^+/−^* mice exhibit heart abnormalities early in development: At embryonic day 13.5 (E13.5), 70% of *Nipbl*^+/−^ mice displayed delays in ventricular septal fusion, whereas 3 days earlier, at E10.5, 100% showed right ventricle hypoplasia (*24*). In situ hybridization experiments showed reduced expression of two transcription factors crucial for early heart progenitor cell growth and differentiation: *Nkx2-5* and *Mesp1* (*24*). That structural abnormalities may begin at the earliest stages of heart development was suggested by results in in *nipbl*-morphant zebrafish, in which defects in the initial migration of cardiogenic mesoderm were detected as early as 18 hours after fertilization (*22*). These results suggested that, at least for CHDs, causal events may occur as early as gastrulation, when the three primary germ layers form and the earliest progenitor cells of major tissues and organs begin to differentiate (*25*).

In this study, we sought to identify developmental alterations during gastrulation that might account for birth defects in *Nipbl*^+/−^ mice. We used single-cell RNA sequencing (scRNA-seq) to compare the cellular compositions, lineage trajectories, and transcriptional landscapes of *Nipbl*^+/−^ mouse embryos to their wild-type (WT) counterparts at two stages spanning the end of gastrulation: the late bud (LB) stage (E7.5) and cardiac crescent (CC) stage (E7.75). Our findings reveal that *Nipbl*^+/−^ embryos have the same cell populations as WT embryos but display subtle misallocation of specific mesodermal cell populations. Our evidence suggests that this occurs as a result of alterations in specific cell fate decisions, including the choice by mesoderm cells to progress toward a noncardiac versus cardiac fate. Our observations strongly suggest that these events cannot be attributed to changes in apoptosis, cell proliferation, global developmental delay, or the structure of cell lineages. As in earlier research, we observed that most gene expression changes in *Nipbl*-deficient tissues at these stages were small (*18*), but we also identified several key developmental genes that are more markedly misexpressed in *Nipbl*^+/−^ embryos, the most notable of which was *Nanog*. *Nipbl*^+/−^ embryos failed to down-regulate *Nanog* at the end of gastrulation, which normally occurs in all but germ cells. As a result, the misexpression of many *Nanog* target genes was observed in all germ layers. We also saw substantial underexpression of *Hox* genes and overexpression of Nodal signaling genes, which play roles in anterior-posterior and left-right patterning, respectively. As a result, we propose a model in which birth defects in CdLS arise from the prolonged overexpression of *Nanog* and dysregulation of developmental pathways governing axial patterning, resulting in the misdirection of cell fate decisions, and misallocation of specific progenitor cell populations.

## RESULTS

### Gastrula-stage *Nipbl*^+/−^ mice display the same cell populations as are found in WT mice

**Fig. 1.**
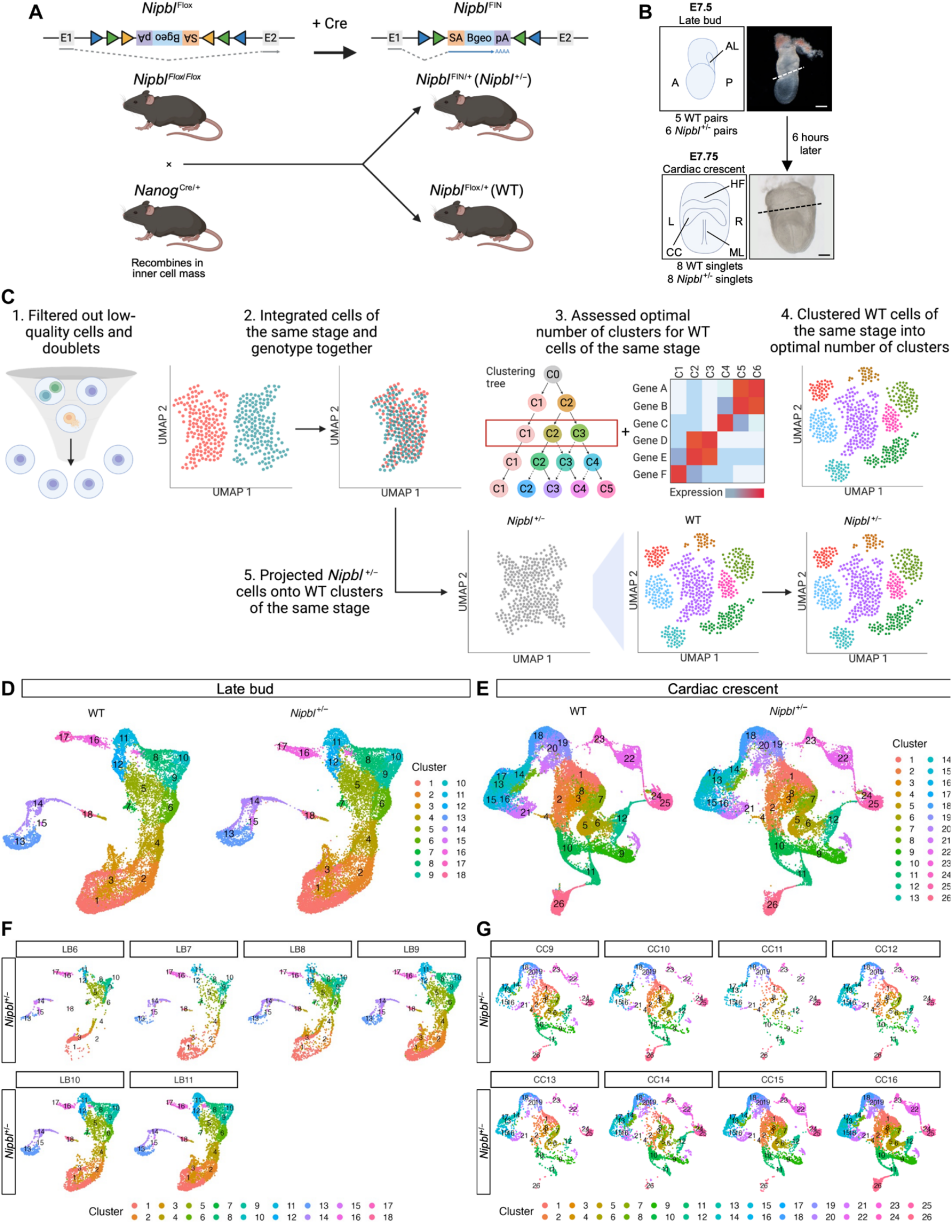
*Nipbl*^+/−^ mice do not lack any cell populations found in WT mice. (**A**) *Nipbl* alleles used in this study. *Nipbl*^Flox^ contains an inverted gene trap cassette encoding β-*geo* flanked by *Cre* recombinase target sites in intron 1 of *Nipbl* gene alleles (*24*). In this inverted orientation, there is no trapping of the *Nipbl* gene, and *Nipbl* is expressed normally. However, when this cassette is exposed to Cre recombinase, the gene trap cassette gets inverted producing the *Nipbl*^FIN^ allele. In this orientation, trapping of the *Nipbl* gene occurs, and β-*geo* is expressed as a reporter of successful gene trapping. When *Nanog*^Cre/+^ mice are mated with *Nipbl*^Flox/Flox^ mice, the resulting littermates are either entirely *Nipbl*^Flox/+^ or entirely *Nipbl*^FIN/+^, as *Nanog*^Cre/+^ mice carry a transgene that expresses *Cre* recombinase in the earliest cells of the developing embryo (*26*). (**B**) Lateral view of LB stage and anterior view of CC-stage embryos subjected to scRNA-seq. A, anterior; P, posterior; AL, allantois; L, left; R, right; HF, head fold; ML, midline. Dashed line represents where embryonic tissue was separated from extraembryonic tissue. Scale bars, 100 μm. (**C**) Workflow used to filter out low-quality cells and doublets, cluster WT cells into optimal number of clusters at each stage, and project *Nipbl^+/−^* cells onto WT clusters of the same stage. Uniform manifold approximation and projection (UMAP) of clusters in WT and *Nipbl^+/−^* embryos at (**D**) LB and (**E**) CC stages. UMAP of clusters in each *Nipbl*^+/−^ embryo at (**F**) LB and (**G**) CC stages.

To investigate early factors influencing birth defects in CdLS, we generated WT and *Nipbl*^+/−^ littermate embryos by crossing *Nanog*^Cre/+^ mice (*26*) with *Nipbl*^Flox/Flox^ mice (*24*) as described in Materials and Methods and shown in Fig. 1A. To ensure an accurate accounting of cell populations and proportions at well-defined stages of early embryonic development, we generated a large excess of embryos and sorted them into groups of narrowly defined stage based on morphological criteria (*27*). Using the 10x Genomics Chromium Single Cell Expression platform (*28*), we performed scRNA-seq on five WT and six *Nipbl^+/−^* samples of LB-stage embryos (E7.5) and eight WT and eight *Nipbl^+/−^* samples of CC-stage embryos (E7.75) (Fig. 1B and fig. S1). Given the low cell numbers in individual LB-stage embryos and the 10x Genomics Chromium Single Cell Expression system’s capture efficiency, samples at the LB stage consisted of pairs of embryos, whereas at the CC stage, they consisted of single embryos. Pairing LB-stage embryos facilitated the capture of rare cell groups, such as primordial germ cells (PGCs), which typically comprise about 50 cells per embryo at this stage (*29*).

At LB stage, we captured a median of 2537 cells per sample, with a median of 17,301 RNA transcripts per cell (fig. S2 and table S1). At CC stage, we captured a median of 4116 cells per sample, with a median of 14,997 RNA transcripts per cell (table S1). As expected, all *Nipbl*^+/−^ embryos expressed both *Cre* and *Betageo*, which report an inactivated *Nipbl* allele (*24*), while WT embryos did not (fig. S3, A and B). *Nipbl*^+/−^ embryos across both stages expressed *Nipbl* at levels that were ~50% lower than WT counterparts (fig. S3C).

We corrected for batch effects among embryos of identical stage and genotype using Seurat’s integration protocol (*30*) (Fig. 1C and fig. S4), which identifies anchors (cells of similar gene expression across samples of the same biological condition) and uses them to align cells into a space shared by all samples. Recognizing that all cells in *Nipbl*^+/−^ tissues exhibit substantial gene expression changes (*18*) and considering that clustering algorithms rely on differences in gene expression among cells (*30*), we implemented measures to prevent our cell clustering from being skewed by gene expression alterations attributable to *Nipbl* haploinsufficiency. We did this by first clustering cells from WT samples (Fig. 1C), using a robust iterative clustering method that considered both intracluster stability and intercluster variation (fig. S5) to determine the optimal number of clusters for cells at each developmental stage. WT cells were clustered into 18 cell populations at LB stage (Fig. 1D) and 26 cell populations at CC stage (Fig. 1E). We then projected *Nipbl*^+/−^ cells onto these WT cell populations at corresponding stages (Fig. 1C). We observed that all *Nipbl*^+/−^ cells could be projected to WT cell populations, with each WT population receiving some *Nipbl*^+/−^ cells (Fig. 1, D and E). This pattern held true across individual *Nipbl*^+/−^ samples, as well as the total *Nipbl*^+/−^ cells in aggregate at each stage (Fig. 1, F and G). To ensure that any differences observed in cell populations were not the result of technical artifacts introduced by the projection algorithm, we performed additional studies in which WT cells were projected onto *Nipbl*^+/−^ clusters (figs. S6 and S15). In both cases, *Nipbl*^+/−^ cells contributed to all clusters but, as we describe later, in varying proportions.

### scRNA-seq captured cells from all germ layers and progenitors of major tissues

**Fig. 2.**
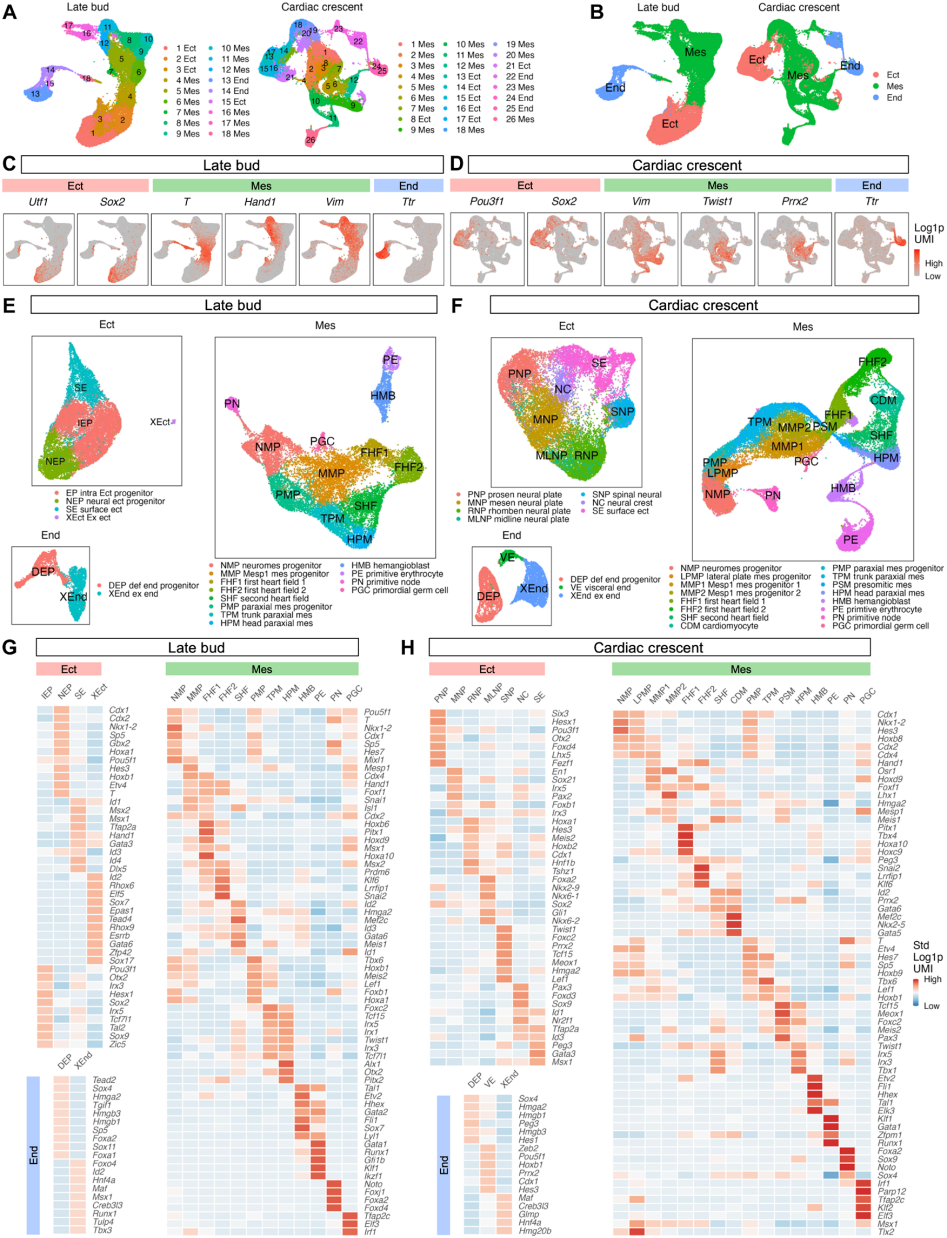
scRNA-seq captured cells from all germ layers, as well as progenitors of major tissues. (**A**) UMAP of clusters assigned to germ layers in LB- and CC-stage embryos. (**B**) UMAP of germ layers in LB- and CC-stage embryos. Expression of genes marking germ layers of (**C**) LB- and (**D**) CC-stage embryos in UMAP. UMAP of cell populations in germ layers of (**E**) LB- and (**F**) CC-stage embryos. Heatmap of fold change in expression of the most differentially expressed transcription factor genes (lowest *Q* values from Mann-Whitney *U* test) (*101*) of cell populations from all other cell populations in germ layers of (**G**) LB- and (**H**) CC-stage embryos.

To assign biological identities to clustered cell populations, we used WT cells as a reference. For each WT cell population, we performed differential gene expression analysis (DGEA) by comparing it to all other cells within embryos of identical stage (Mann-Whitney *U* test). We detected markers indicative of germ layer identity in every cluster (data S1 and S2 and table S2), enabling us to associate each cluster with a specific germ layer at each developmental stage (Fig. 2A). Figure 2B displays all cells from all samples, categorizing them into germ layers at both LB and CC stages of development. Figure 2 (C and D) further depicts the assignment of cells based on the expression of germ layer markers, at LB and CC stages, respectively. These markers include those for ectoderm, such as *Utf1*, *Sox2*, and *Pou3f1* (*31**–**33*), mesoderm, including *T*, *Hand1*, *Vim*, *Twist1*, and *Prrx2* (*34*–*38*), and endoderm, *Ttr* (*39*).

To identify cell types within germ layers, we reapplied DGEA after clustering individual germ layers. For the endoderm, this enabled us to identify definitive endoderm progenitors (DEPs) (*Sox4* and *Foxa2*) (*40*, *41*), as well as extraembryonic endoderm (XEnd) (*Hnf4a* and *Tbx3*) (*42*, *43*) at LB stage (Fig. 2E, data S3, and table S3). In CC-stage embryos, we captured identical cell types in the endoderm and visceral endoderm (VE) (*Hnf4a* and *Esx1*) (Fig. 2F, data S4, and table S3) (*42*, *44*). DEPs and XEnd give rise to the gut and placenta, respectively.

Within the ectoderm of LB-stage embryos, we identified early progenitor populations including intraembryonic ectoderm progenitors (IEPs) (*Pou3f1* and *Otx2*) (*33*, *45*), neural ectoderm progenitors (NEPs) (*Cdx1* and *Nkx1-2*) (*46*, *47*), and surface ectoderm (SE) (*Id1*, *Msx2*, and *Msx1*) (*48*) (Fig. 2, E and G, data S5, and table S4). In addition, we detected a small portion of extraembryonic ectoderm (XEct) (*Elf5* and *Tead4*) (*49*, *50*), which was expected, as a small quantity of XEct cells was included in our dissection. By CC stage, IEPs, NEPs, and XEct were no longer detectable, a phenomenon previously noted (*51*). Rather, we continued to detect SE. We also detected six distinct neural populations (Fig. 2, F and H, data S6, and table S4). These neural populations included prosencephalic neural plate (*Six3* and *Hesx1*) (*52*, *53*), mesencephalic neural plate (*En1* and *Pax2*) (*54*, *55*), rhombencephalic neural plate (*Hoxa1* and *Hes3*) (*56*, *57*), midline neural plate (*Foxa2* and *Nkx2-9*) (*58*, *59*), spinal neural plate (*Sox2* and *Ezr*) (*60*, *61*), and neural crest (*Pax3* and *Foxd3*) (*62*, *63*) (data S6). Collectively, these populations constitute the nascent progenitors of the brain, spinal cord, and peripheral nervous system.

Within the mesoderm of LB-stage embryos, we identified six distinct groups of cells, detailed in Fig. 2 (E and G), data S7, and table S5. These consisted of (i) neuromesodermal progenitors (NMPs) expressing markers *T* and *Nkx1-2* (*34*, *47*); (ii) derivatives of NMPs including *Mesp1*-expressing mesoderm progenitors (MMPs) (*Mesp1*) (*64*), two types of first heart field (FHF) cells (FHF1 and FHF2) marked by *Hand1* (*35*), cells of the second heart field (SHF) with *Id2* and *Mef2c* (*65*, *66*), and three kinds of paraxial mesoderm (PM) cells, further categorized into PM progenitors (PMPs) (*Tbx6* and *Meis2*) (*67*, *68*), trunk PM (TPM) (*Foxc2* and *Tcf15*) (*69*, *70*), and head PM (HPM) (*Alx1* and *Tcf15*) (*70*, *71*); (iii) hematopoietic cells including hemangioblasts (HMBs) (*Tal1* and *Etv2*) (*72*, *73*) and primitive erythrocytes (PEs) (*Gata1* and *Tal1*) (*72*, *74*); and (iv) two rare populations comprising cells of the primitive node (PN) (*Noto* and *Foxj1*) (*75*, *76*) and PGCs (*Tfap2c* and *Msx1*) (*77*, *78*). At CC stage, we detected all mesodermal populations found earlier, along with several that emerge later in development. These comprised lateral plate mesoderm progenitors (LPMPs) (*Tlx2* and *T*) (*34*, *79*), additional populations of MMPs (MMP1 and MMP2) (*Mesp1*) (*64*), cardiomyocytes (CDMs) (*Mef2c* and *Nkx2-5*) (*80*, *81*), and presomitic mesoderm (PSM) (*Tcf15* and *Meox1*) (*70*, *82*) (Fig. 2, F and H, data S8, and table S5). Collectively, these progenitors are responsible for the formation of structures including the skeletal muscle, bone, blood, and heart.

### *Nipbl^+/−^* mice have fewer mesoderm cells, PEs, FHF cells, and more PM cells

**Fig. 3.**
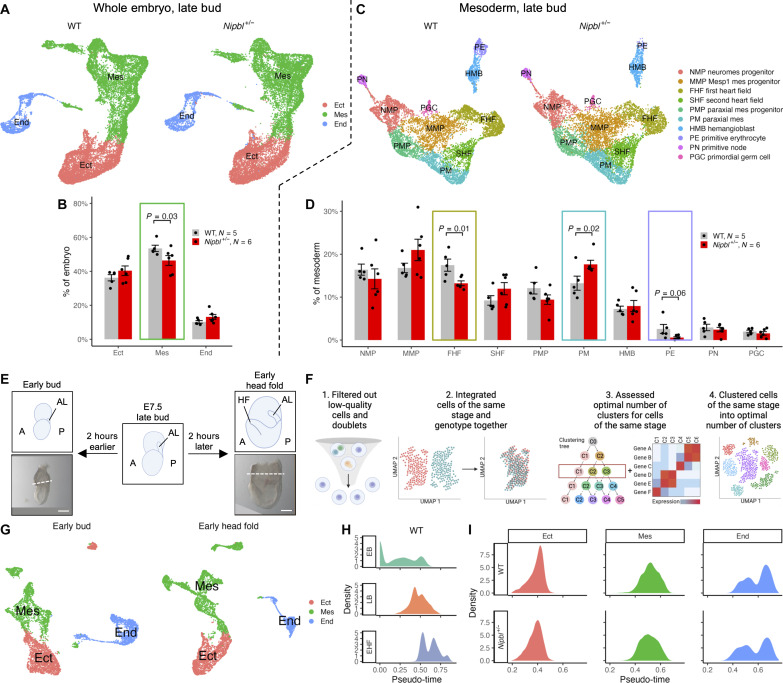
*Nipbl^+/−^* embryos exhibit changes in the sizes of mesodermal subpopulations that are not accompanied by changes in developmental timing. (**A**) UMAP of germ layers in LB-stage WT and *Nipbl*^+/−^ embryos. (**B**) Percentage of cells in germ layers from all cells in LB-stage WT and *Nipbl^+/−^* embryos. Error bars show SEM. *P* values from *t* test. (**C**) UMAP of cell populations in mesoderm of LB-stage WT and *Nipbl^+/−^* embryos. (**D**) Percentage of cells in mesodermal cell populations from all cells in LB-stage WT and *Nipbl^+/−^* embryos (bottom). Error bars show SEM. *P* values from *t* test. (**E**) Lateral view of WT EB- and EHF-stage embryos subjected to scRNA-seq. Dashed line represents where embryonic tissue was separated from extraembryonic tissue. Scale bars, 100 μm. (**F**) Workflow used to filter out low-quality cells and doublets and cluster cells at EB and EHF stages into optimal number of clusters. (**G**) UMAP of clusters assigned to germ layers in EB- and EHF-stage embryos. (**H**) Density of cells from WT EB-, LB-, and EHF-stage embryos along pseudo-time (calculated using URD). (**I**) Density of cells in germ layers of LB-stage WT and *Nipbl*^+/−^ embryos along pseudo-time.

We analyzed the cellular composition of embryos at both LB and CC stages, quantifying the proportion of cells within each germ layer that contributed to the total cell count. LB-stage mutants exhibited 13% fewer mesoderm cells (Fig. 3, A and B, and data S9), while concurrently displaying a greater proportion of endoderm and ectoderm cells. We sought to ascertain whether this decrease in mesoderm was generalized or restricted to specific subpopulations. Focusing on LB-stage embryos, we merged mesodermal cell subpopulations of similar biological identity together (FHF1 + FHF2 into FHF and PSM + HPM into PM) (Fig. 3C) and calculated the percentage of cells across these subpopulations, relative to the overall mesoderm. The findings (Fig. 3D) revealed a 77% reduction in PEs *Nipbl*^+/−^ embryos (*P* = 0.06, *t* test; data S10). In addition, differences were observed in two mesodermal derivatives of the neuromesoderm: (i) Mutants had 24% fewer FHF cells (Fig. 3D); and (ii) *Nipbl*^+/−^ embryos showed a contrasting pattern in PM, a 33% increase in PM cells compared to WT (Fig. 3D).

Since *Nipbl^+/−^* cells were projected onto WT cell populations, we wondered whether any observed differences between *Nipbl^+/−^* and WT embryos might have occurred as a result of misprojecting cells, i.e., assigning cells to incorrect clusters. To investigate this, we independently clustered *Nipbl^+/−^* cells and projected WT cells onto those clusters; we refer to this as reverse projection (fig. S6). If observed differences in the allocation of *Nipbl^+/−^* cells to particular clusters had been a result of misclassification due to technical limitations of projecting cells, then the same differences should not appear when reverse projection was done. If misclassified cells were simply distributed at random one should expect reverse projection to produce differences opposite to those produced by the original, forward projection. However, this was not found to be the case. Cluster assignments resulting from reverse projection quantitatively resembled those of forward projection (fig. S6, A to D). Germ layer identities, which were based on the expression of specific markers (table S6 and data S11), were generally the same in the reverse projection analysis as well (fig. S6, E to H).

Last, we quantified the proportion of cells within each germ layer and mesodermal population following reverse projection. Consistent with the findings from forward projection (Fig. 3B), *Nipbl^+/−^* embryos showed 13% fewer mesoderm cells than WT embryos, while concurrently showing more endoderm and ectoderm cells (fig. S6M and data S13). Likewise, the mesoderm of *Nipbl^+/−^* embryos showed fewer FHF cells (24% fewer) and more PM cells (22% more) than WT embryos (Fig. 3D, fig. S6N, and data S14). [Because very few PEs are present in *Nipbl^+/−^* embryos (Fig. 3D), it was not possible to cluster these cells into a distinct cell population during reverse projection (fig. S6, K and L).] Together, these data indicate that differences in cell population sizes observed in *Nipbl^+/−^* embryos represent true biological changes and are not a consequence of technical issues, such as misprojection.

### *Nipbl^+/−^* embryos do not show changes in developmental timing

Cellular composition changes very quickly during and after gastrulation; one possible explanation for differences in proportions of cell types could be a change in the overall pace of development, such that embryos of one genotype were slightly delayed or accelerated relative to the other. To investigate whether this was the case for *Nipbl^+/−^* embryos, we used pseudo-temporal ordering. To ensure that pseudo-time accurately mirrored real developmental time, we augmented our analysis with scRNA-seq data from an additional two pairs of WT embryos at the early bud (EB) stage and two pairs of WT embryos at the early head fold (EHF) stage (Fig. 3E and fig. S7). EB-stage embryos are 2 hours younger and EHF-stage embryos are 2 hours older than LB-stage embryos, so these supplementary samples provided the temporal resolution necessary to discern even minor changes (less than 2 hours) in developmental timing (*27*). We eliminated low-quality cells and doublets (Fig. 3F, fig. S8, and table S8) before integrating cells of the same stage (fig. S9). We then clustered EB-stage cells into 8 distinct populations and EHF-stage cells into 13 distinct populations (fig. S10, A and B). Each population was subsequently annotated as ectoderm, mesoderm, or endoderm (Fig. 3G and fig. S10, C and D) based on the expression of specific germ layer markers (data S15 and S16 and table S9).

We ordered cells from each embryo at every developmental stage (EB, LB, and EHF) using URD (*83*), which constructs a diffusion map of transition probabilities and, starting with an assigned group of root cells, performs a probabilistic breadth-first graph search using the transition probabilities. When we visualized the arrangement of cells from each stage based on their density in pseudo-time, we found that cells from WT embryos ordered in accordance to their developmental stage: EB came first, followed by LB, and lastly EHF (Fig. 3H and data S17), while showing partial overlap between cells of different stages. This confirmed that the pseudo-time values we acquired were an accurate representation of actual developmental timing. When the pseudo-time orderings of WT and *Nipbl^+/−^* LB-stage embryos were compared with each other, we found no statistically significant deviations (Kolmogorov-Smirnov test) (Fig. 3I). These findings argue that the observed reduction in mesoderm cells in *Nipbl^+/−^* mice at LB stage cannot be attributed to overall developmental delay or acceleration. The fact that *Nipbl* haploinsufficiency does not result in global changes in developmental timing strongly suggests that birth defects in CdLS result from specific cellular or molecular irregularities within individual developmental pathways and not a globally altered developmental timeline.

### *Nipbl^+/−^* embryos do not show changes in cell lineage trajectory

**Fig. 4.**
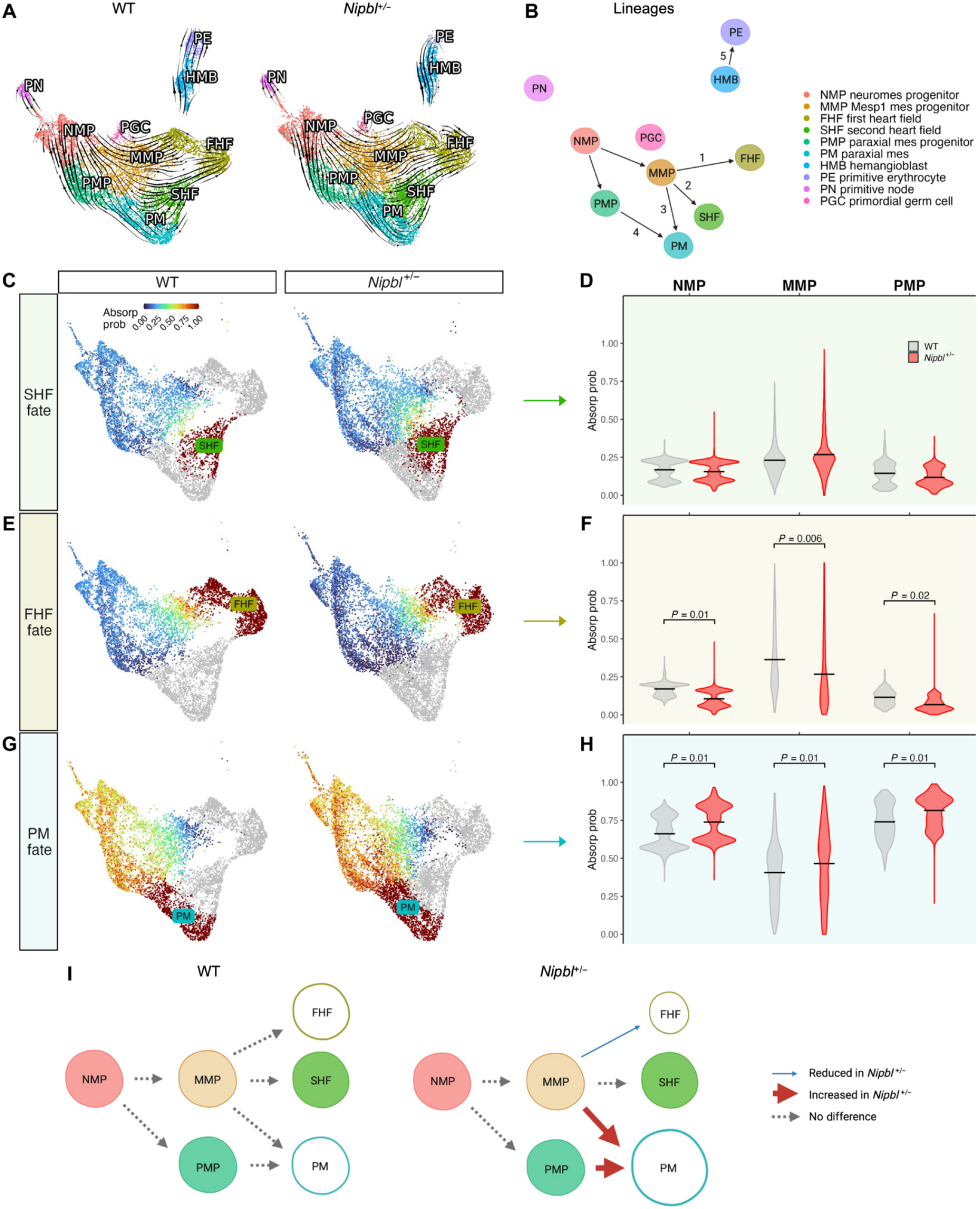
*Nipbl^+/−^* mice misdirect mesoderm cells into PM at the expense of the FHF. (**A**) RNA velocities, calculated by scVelo of mesoderm cells from LB-stage WT and *Nipbl^+/−^* embryos in UMAP. (**B**) Cell lineage trajectories in mesoderm of LB-stage WT and *Nipbl^+/−^* embryos. Probability of mesoderm cells from LB-stage WT and *Nipbl^+/−^* embryos terminally transitioning (absorption probabilities calculated using CellRank) into (**C**) SHF, (**E**) FHF, or (**G**) PM fates in UMAP. Violin plot of probability of NMPs, MMPs, and PMPs from LB-stage WT and *Nipbl^+/−^* embryos terminally transitioning (absorption probabilities) into (**D**) SHF, (**F**) FHF, or (**H**) PM fates. Lines show means. *P* values from *t* test. (**I**) Schematic illustrating how mesoderm cells in *Nipbl^+/−^* embryos are misdirected into PM fate at the expense of FHF fate.

To discern whether alterations in the sizes of cell population in *Nipbl^+/−^* embryos were attributable to changes in the structures of cell lineages, we used scVelo (*84*) to compute the RNA velocity for all mesoderm cells from LB-stage embryos. RNA velocity provides a predictive metric for a cell’s future transcriptional state, gauging the equilibrium between the synthesis of spliced mRNA from unspliced mRNA and mRNA degradation. scVelo was used to build cell lineage trajectories separately for WT and *Nipbl^+/−^* embryos at LB stage (Fig. 4A). In WT mesoderm, five distinct lineages were discernible (Fig. 4B and data S18), with NMPs giving rise to three terminal fates via four specific pathways. These pathways encompassed the differentiation of NMPs into FHF, SHF, and PM. Note that *Mesp1*-expressing cells have the versatility to differentiate into both cardiac mesoderm and PM. While *Mesp1* is typically recognized as a cardinal factor for cardiac specification (*85*), our findings suggest that its influence may extend beyond this role into paraxial specification as well. As a result, the PM has dual progenitors, PMPs and MMPs. We also observed a fifth lineage, independent of NMP lineages, where HMBs differentiate into PEs. Notably, PGCs and PN cells did not form part of any identified lineages. Examination of the mesoderm in *Nipbl^+/−^* embryos revealed lineage trajectories identical to those in WT (Fig. 4A and data S19). These observations suggest that variations in the overall structures of cell lineage pathways do not explain differences in the number of FHF and PM cells in *Nipbl^+/−^* embryos (Fig. 3, B and D).

### *Nipbl^+/−^* embryos misdirect mesoderm cells into PM at the expense of the FHF

To explore whether variations in cell fate decisions might be responsible for the observed differences in the numbers of FHF and PM cells in *Nipbl^+/−^* embryos, we used CellRank (*86*) to compute the likelihood of cells from the mesoderm (excluding HMBs, PEs, PGCs, and PN cells) transitioning terminally into cells of SHF, FHF, or PM fate (data S20 and S21). HMBs, PEs, PGCs, and PN were omitted from the calculation because our trajectories derived from RNA velocity indicated that these populations do not ultimately transition into SHF, FHF, or PM. In CellRank, the absorption probability defines that the likelihood that a specific cell type will make a transition to a terminal state, derived using RNA velocity–directed, random walks from initial to terminal cell states (*86*). We represented these probabilities in uniform manifold approximation and projection (UMAP) space, with shades of blue to signify low probabilities and red for high probabilities. Figure 4 (C and D) illustrates that the probabilities of NMPs, MMPs, and PMPs making a terminal transition solely into SHF do not vary significantly between *Nipbl^+/−^* and WT embryos, as quantified in the adjacent violin plots. However, the transition probabilities into FHF are 27 to 48% lower in *Nipbl^+/−^* embryos (Fig. 4, E and F), while the probabilities for transitions into PM are 9 to 11% higher (Fig. 4, G and H). Together, these results support the hypothesis that disparities in cell numbers in *Nipbl^+/−^* embryos stem from a misdirection of cells from FHF pathway toward a PM fate (Fig. 4I).

### *Nipbl^+/−^* embryos do not show evidence of altered apoptotic activity or cell proliferation

Altered cell numbers in different mesodermal subpopulations of *Nipbl^+/−^* embryos could also conceivably stem from modifications in rates of cell death or proliferation. Focusing on apoptotic activity, we carried out DGEA on WT and *Nipbl^+/−^* embryos at LB stage, using genes associated with apoptosis from two well-known gene sets: Reactome Apoptosis and Hallmark Apoptosis (*87*, *88*). We ranked each gene by minimum *Q* value, arranging them from lowest to highest, and visualized their expression across all germ layers in *Nipbl^+/−^* embryos as heatmaps of the fold change relative to their WT counterparts (fig. S11, A and B, and data S22). For both gene sets, we saw no visible pattern of differences in the expression of genes with even the lowest *Q* values between any of the germ layers in both WT and *Nipbl^+/−^* embryos. Although genes from these gene sets tend to mark cells with the capacity for apoptosis, their expression levels are not necessarily indicative of cells actively undergoing apoptosis. We therefore also turned to a gene set identified in a study that compared 180 apoptosis-associated genes in hematopoietic cells that were either healthy or undergoing apoptosis (*89*) and identified 93 apoptosis-associated genes that were differentially expressed. When we examined these genes in the germ layers of *Nipbl^+/−^* embryos, we also saw no visible difference with WT embryos (fig. S11C and data S22). These data suggest that changes in apoptotic activity are unlikely to be a major contributor to differences in cell type proportions.

To examine cell proliferation, we again used DGEA across all germ layers of both WT and *Nipbl^+/−^* embryos at LB stage, focusing on the meta–proliferating cell nuclear antigen (PCNA) gene set, an ensemble of genes that exhibit the strongest positive correlation with *PCNA* expression, a recognized biomarker of cell proliferation (*90*). When we visualized the expression of these genes in the same manner as the gene sets above, we found no discernible variations in the expression levels of the meta-PCNA genes between the germ layers of WT and *Nipbl^+/−^* embryos (fig. S12A and data S23). As another measure of cell proliferation, we used Seurat to assign cells from LB-stage WT and *Nipbl^+/−^* embryos into phases (G_1_, S, and G_2_-M) of the cell cycle (*91*). Seurat does this by calculating a cell cycle phase score based on the expression of canonical S phase and G_2_-M phase markers. Seurat considers these marker sets to be anticorrelated in their expression, so when cells express neither, they are considered to be in G_1_ phase. As expected, in all germ layers, cells from LB-stage WT and *Nipbl^+/−^* embryos assigned into all phases of the cell cycle (fig. S12B and data S24). In all germ layers, there was no statistically significant difference in the proportions of cells in each cell cycle between WT and *Nipbl^+/−^* embryos (fig. S12C). We therefore concluded that extensive alterations in cell proliferation are not likely responsible for disparities in cell subpopulations.

### *Nipbl*^+/−^ mice underexpress genes predicted to drive the transition of mesoderm cells into FHF

**Fig. 5.**
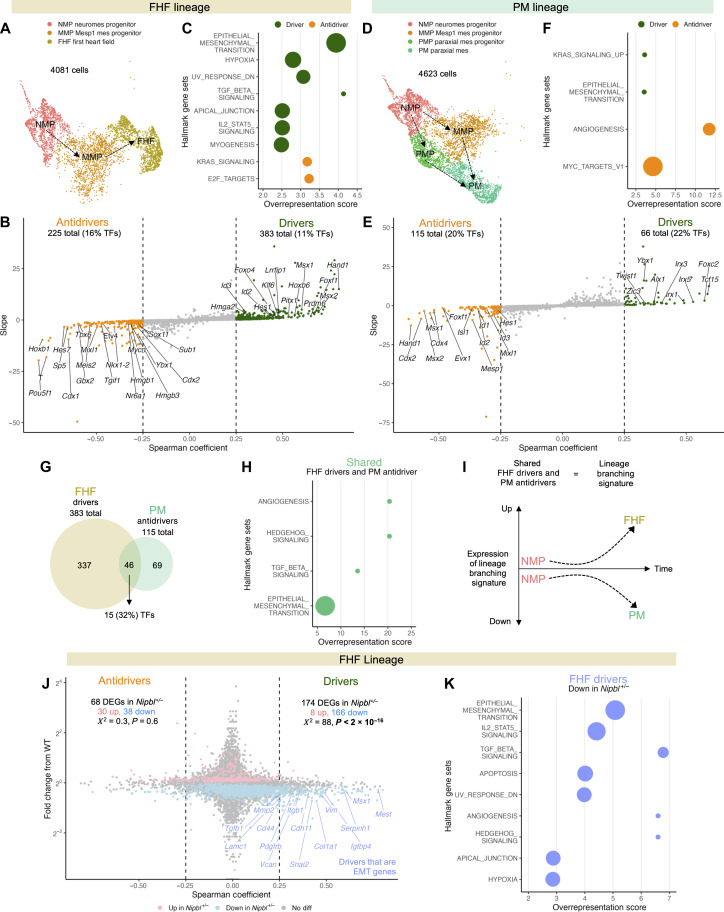
*Nipbl*^+/−^ mice underexpress genes predicted to drive the transition of mesoderm cells into FHF. UMAP of mesoderm cells in (**A**) FHF and (**D**) PM lineages of WT LB-stage embryos. Genes whose expression is positively correlated (drivers) or negatively correlated (antidrivers) with the transition (absorption probabilities from Fig. 3J) of mesoderm cells from WT LB-stage embryos into (**B**) FHF or (**E**) PM fates. Correlation coefficients calculated using Spearman’s rank correlation. Genes with correlation coefficient greater than 0.25 or less than −0.25 were considered drivers and antidrivers, respectively. Slope refers to change in gene expression along absorption probability. Overrepresentation score of MSigDB’s Hallmark gene sets among drivers and antidrivers of (**C**) FHF and (**F**) PM fates. (**G**) Venn diagram of genes shared between PM antidrivers and FHF drivers. (**H**) Overrepresentation score of MSigDB’s Hallmark gene sets among shared FHF drivers and PM antidrivers. (**I**) Expression of lineage branching signature as NMPs transition into FHF or PM fates. (**J**) Fold change in expression of genes in mesoderm cells of FHF lineage of LB-stage *Nipbl*^+/−^ embryos differentially expressed (*Q* < 0.05, Mann-Whitney *U* test) from that of WT embryos along Spearman’s rank correlation coefficient from (B). Genes associated with EMT are labeled. (**K**) Overrepresentation score of MSigDB’s Hallmark gene sets among shared FHF drivers down-regulated in FHF lineage of *Nipbl^+/−^* mice. TFs, transcription factor.

Next, we sought to investigate whether the misdirection of mesoderm cells in *Nipbl^+/−^* embryos at the LB stage could be attributed to the misexpression of genes that drive the transition of mesoderm cells into either FHF or PM. To answer this question, we also used CellRank to identify potential driver genes of the FHF and PM fates, using those mesoderm cells that specifically contribute to these developmental pathways (Fig. 5, A and D). CellRank achieves this by calculating a correlation coefficient between the likelihood (absorption probabilities) of cells progressing toward a particular lineage fate and the expression level of individual genes (*86*). In this context, genes with positive correlation coefficients are considered drivers, as their expression elevates alongside an increase in absorption probabilities. Conversely, genes with negative correlation coefficients are deemed antidrivers, as their expression diminishes with increasing absorption probabilities. To minimize the risk of false discoveries, we set a threshold, accepting genes with correlation coefficients greater than 0.25 as drivers and those less than −0.25 as antidrivers.

CellRank identified 383 genes as drivers of FHF fate (Fig. 5B and data S25). Eleven percent of them were transcription factors. Among these were *Hand1* and *Tbx20*, transcription factors previously recognized for their roles in promoting FHF development (*35*, *92*). Several transcription factors that have not been previously linked to FHF development were also predicted to be FHF drivers, including *Msx1*, *Msx2*, *Foxf1*, *Hoxb6*, and *Pitx1*. Of these, *Hoxb6* and *Pitx1* are known to play important roles in segmentation and patterning. Gene set overrepresentation analysis using the Molecular Signatures Database (MSigDB)’s Hallmark gene sets (*87*), which characterize well-defined biological states and processes, revealed that FHF drivers were significantly enriched in genes associated with transforming growth factor–β (TGF-β) signaling (Fig. 5C), which has been previously described as important in CDM proliferation (*93*). Genes associated with epithelial-to-mesenchymal transition (EMT) were also significantly enriched (see below). Conversely, CellRank predicted 225 genes to be antidrivers of FHF fate, with 16% being transcription factors. Among these were genes known to be involved in pluripotency (*Pou5f1*), mesoderm specification/expansion (*T*, *Cdx1*, *Sp5*, and *Zic3*), anterior-posterior patterning (*Hoxb1* and *Hoxa1*), and PM development (*Hes7*, *Meis2*, *Tbx6*, *Gbx2*, and *Foxb1*).

CellRank predicted 66 genes to be drivers and 115 genes to be antidrivers of PM fate (Fig. 5E and data S26). A substantial proportion (22%) of PM drivers were transcription factors. These included *Tcf15* and *Foxc2*, transcription factors that have been previously recognized for their role in regulating PM development (Fig. 5E) (*69*, *94*). The *Irx* transcription factors *Irx5*, *Irx1*, and *Irx3*—known for their roles in segmentation during development (*95*)—were also identified as PM drivers. PM drivers did not exhibit significant overrepresentation of any Hallmark gene sets (Fig. 5F). Of all PM antidrivers, 20% were identified as transcription factors, including *Hand1*, *Cdx2*, *Msx2*, *Msx1*, and *Cdx4*. *Hand1*, *Msx2*, and *Msx1* had also been predicted as drivers for FHF. This overlap prompted us to conduct a comparative analysis between PM antidrivers and FHF drivers. Our examination revealed that 40% of all PM antidrivers were concurrently FHF drivers (Fig. 5, G and H).

The confluence of expressed genes between FHF drivers and PM antidrivers seems to indicate that during normal development, increased expression of these shared genes by mesoderm cells predisposes them to transition into FHF, while decreased expression of these same genes steers them toward PM (Fig. 5I). This set of shared genes may thus be considered to be a “lineage branching signature.”

How does *Nipbl* haploinsufficiency affect the expression of driver and antidriver genes? Examination of *Nipbl*^+/−^ embryos revealed significant misexpression in NMP, MMP, and PMP cells of numerous driver and antidriver genes associated with both FHF and PM (Fig. 5J, fig. S13, and data S27 and S28). Most affected driver and antidriver genes were down-regulated. To ascertain whether this was more than coincidental, we used a chi-square analysis, which revealed a notable pattern: *Nipbl^+/−^* embryos underexpressed FHF drivers at a higher frequency than they overexpressed them [166 of 174 differentially expressed genes (DEGs)] (Fig. 5J). Similarly, they commonly underexpressed PM antidrivers (40 of 51) (fig. S13), aligning with expectations since many PM antidrivers serve dual roles as FHF drivers.

Notably, genes associated with EMT were highly enriched among FHF drivers that were underexpressed in *Nipbl^+/−^* embryos (Fig. 5K). A total of 15 EMT genes was found to be underexpressed, including the EMT transcription factors *Msx1* and *Snai2* and signaling genes such as *Igfbp4*, *Pdgfrb*, and *Tgfb1* (Fig. 5J). These results suggest that, in the context of *Nipbl* haploinsufficiency, skewed differentiation of mesoderm cells toward the PM lineage at the expense of FHF lineage may be attributable to the underexpression of genes driving FHF fate. These results are consistent with the idea that genes associated with EMT play a role in this lineage misdirection.

### *Nipbl*^+/−^ mice show large changes in the expression of major developmental regulators in all germ layers

**Fig. 6.**
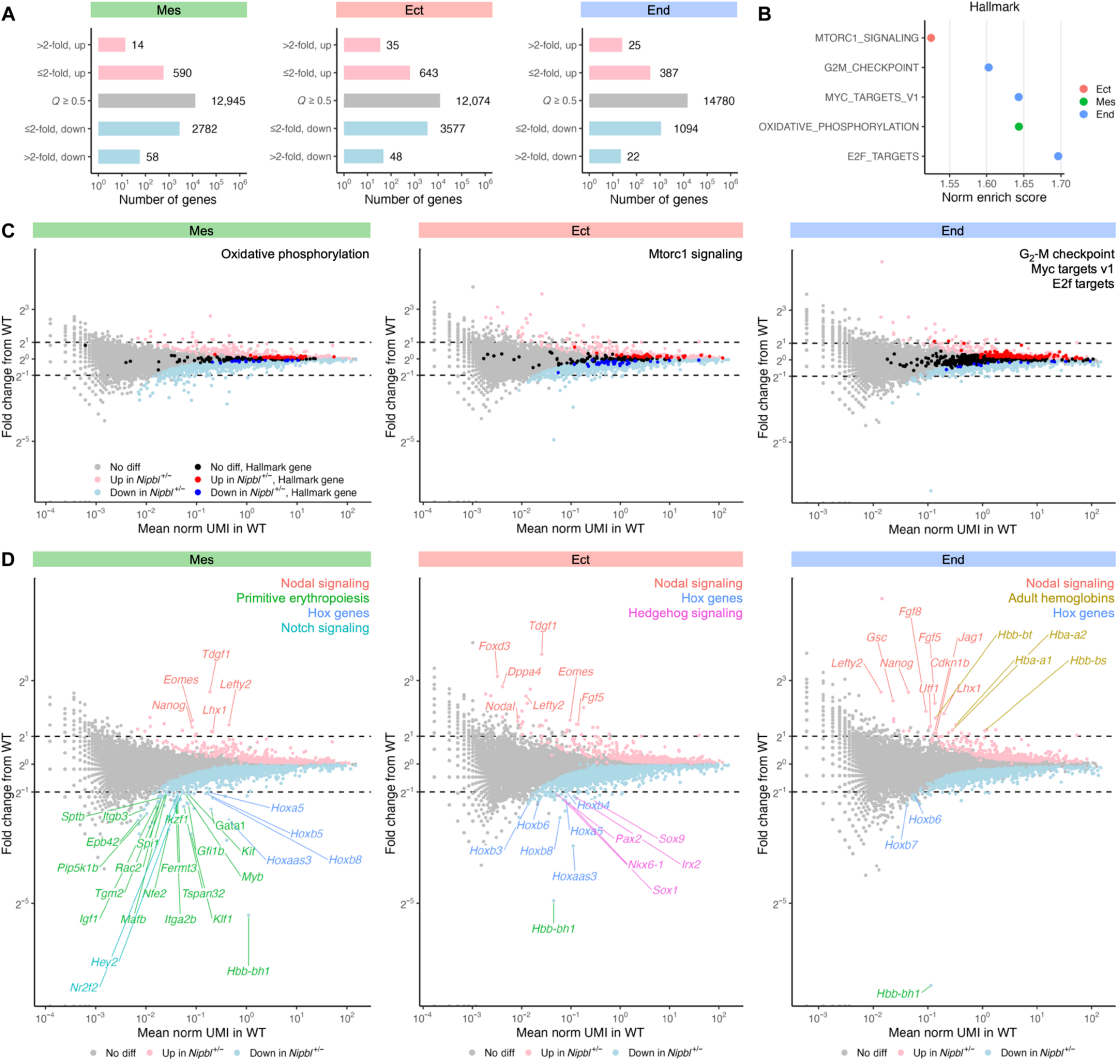
*Nipbl*^+/−^ mice show large changes in the expression of major developmental regulators in all germ layers. (**A**) Number of differentially expressed genes (*Q* < 0.05, Mann-Whitney *U* test) in germ layers of LB-stage *Nipbl*^+/−^ embryos showing small (≤2-fold) or large (>2-fold) changes in expression from that of WT embryos. (**B**) Normalized enrichment score of statistically significant (*Q* < 0.05, FGSEA) Hallmark genes sets in germ layers of LB-stage *Nipbl*^+/−^ embryos from that of WT embryos. (**C**) Fold change in expression of Hallmark gene sets from Fig. 5B in germ layers of LB-stage *Nipbl^+/−^* embryos from that of WT embryos along their average expression in WT embryos. (**D**) Fold change in expression of DEGs (*Q* < 0.05, Mann-Whitney *U* test) in germ layers of LB-stage *Nipbl^+/−^* embryos showing large changes in expression (>2-fold) from that of WT embryos along their average expression in WT embryos.

Using DGEA to compare the germ layers of LB-stage WT and *Nipbl^+/−^* embryos (data S29 to S31), we found that *Nipbl^+/−^* embryos misexpressed hundreds of genes across all germ layers. Underexpression was more common than overexpression in all cases (Fig. 6A), supporting a broad role for *Nipbl* in enhancing gene expression. Most of gene expression changes were subtle, i.e., less than twofold (Fig. 6A). This pattern of gene expression changes agrees with earlier studies (*18*). Gene set enrichment analysis (GSEA) for MSigDB’s Hallmark gene sets found that *Nipbl^+/−^* embryos showed enrichment, and no de-enrichment, for 4 of 40 gene sets: oxidative phosphorylation in the mesoderm; MTORC1 signaling in the ectoderm; and G_2_-M checkpoint, Myc targets, and E2F targets in the endoderm (Fig. 6B). In these cases, however, enrichment was driven by a relatively small number of the genes in these sets; the vast majority was either not differentially expressed in *Nipbl^+/−^* embryos or showed changes much smaller than 1.5-fold (Fig. 6C).

Genes exhibiting substantial changes in expression—greater than twofold up-regulated or down-regulated—were nevertheless observed across every germ layer of *Nipbl^+/−^* embryos (Fig. 6A). Individual curation of these genes unveiled associations with developmental processes (Fig. 6D). Within the mesoderm, two- to sixfold up-regulation of genes involved in Nodal signaling, including *Tdgf1*, *Eomes*, *Lefty2*, and *Lhx1*, was observed (*96**–**99*) . At the same time, three clusters of genes were robustly down-regulated: those associated with primitive erythropoiesis, including *Gata1* and *Klf1* (two- to threefold) (*74*, *100*); *Hox* genes such as *Hoxb8*, *Hoxb5*, and *Hoxa5* (twofold); and genes associated with Notch signaling, including *Hey2* and *Nr2f2* (twofold) (*101*, *102*) (Fig. 6D).

In the ectoderm, five transcription factor genes tied to the hedgehog signaling pathway were down-regulated more than twofold, including *Pax2*, *Sox9*, *Nkx6-1*, *Sox1*, and *Irx2* (*103**–**107*) (Fig. 6D). In the endoderm, there was a distinct two- to threefold up-regulation of several adult hemoglobin genes such as *Hbb-bt*, *Hba-a2*, *Hba-a1*, and *Hbb-bs* (Fig. 6D). Consistent patterns were evident in both the ectoderm and endoderm, mirroring the mesoderm’s trends. Genes related to Nodal signaling were up-regulated, and *Hox* genes were noticeably down-regulated, across all germ layers. The magnitude of changes in Nodal signaling genes and *Hox* genes, being greater than twofold in both directions, and their critical roles in various germ layers, strongly suggests that disruptions in these pathways could be major contributors to phenotypes observed in *Nipbl^+/−^* mice.

When we compared the sets of DEGs obtained from forward projection (Fig. 6) with those obtained from reverse projection (fig. S14), we found that greater than 94% of DEGs in mesoderm and ectoderm were identical (fig. S14, A and B). For the endoderm, reverse projection showed more than 75% of the same DEGs (fig. S14C). As was the case for changes in the relative sizes of cell populations, we conclude that DEGs observed in *Nipbl^+/−^* embryos represent true biological differences and are not a consequence of technical issues in cell classification.

### *Nipbl*^+/−^ mice overexpress *Nanog* during and after gastrulation

**Fig. 7.**
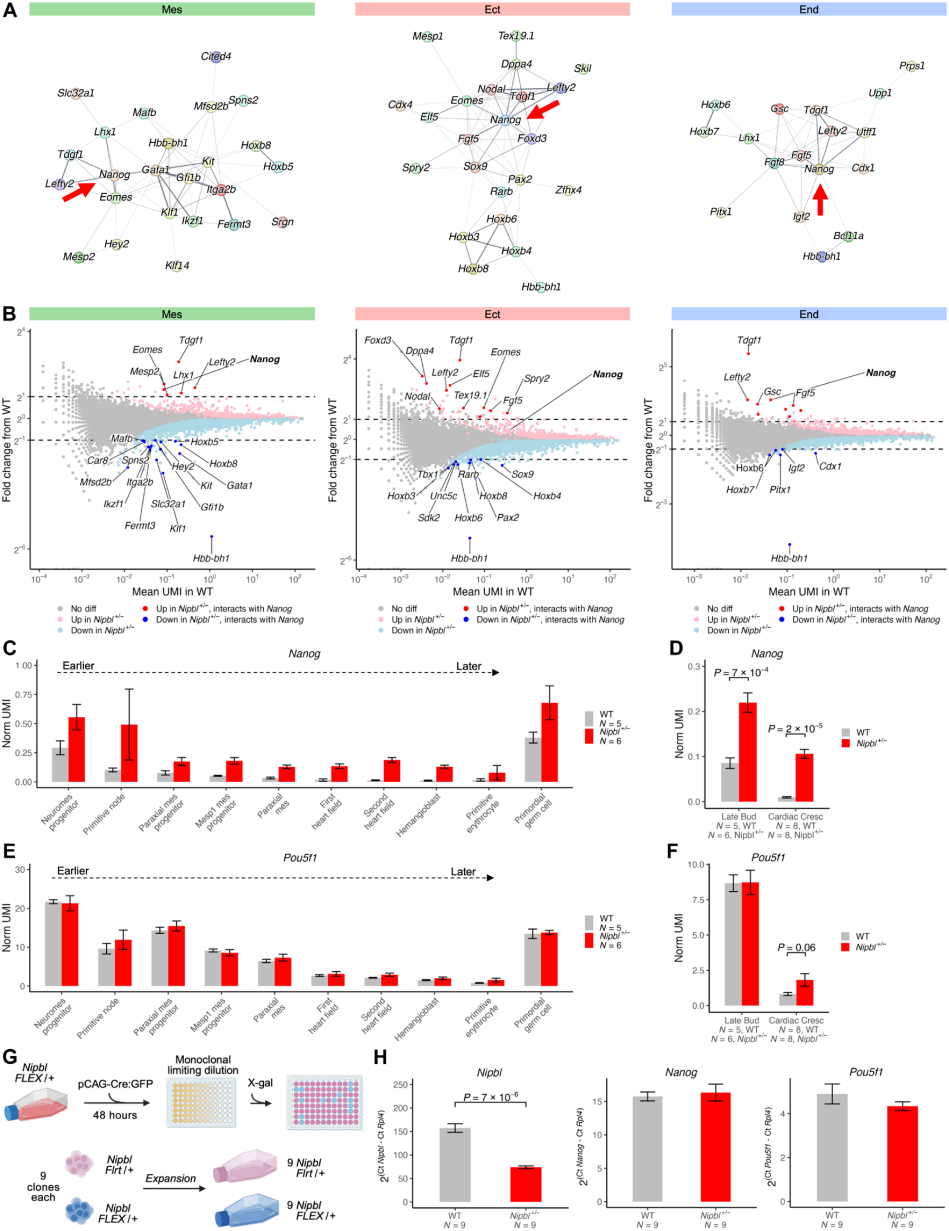
*Nipbl*^+/−^ mice overexpress *Nanog* during and after gastrulation. (**A**) DEGs (greater than twofold up-regulated or down-regulated) between the germ layers of WT and *Nipbl*^+/−^ embryos predicted by STRING to interact with each other. (**B**) Fold change in expression of DEGs (*Q* < 0.05, Mann-Whitney *U* test) in germ layers of LB-stage *Nipbl^+/−^* embryos showing large changes in expression (>2-fold) from that of WT embryos along their average expression in WT embryos that are predicted by STRING to interact with *Nanog*. (**C**) Expression of *Nanog* in mesodermal cell populations of LB-stage WT and *Nipbl^+/−^* embryos ordered from left (earlier) to right (later) by their RNA velocity positions in Fig. 4A. Error bars show SEM. (**D**) Expression of *Nanog* in WT and *Nipbl^+/−^* embryos from LB to CC stages. Error bars show SEM. *P* values from *t* test. (**E**) Expression of *Pou5f1* in mesodermal cell populations of LB-stage WT and *Nipbl^+/−^* embryos ordered from left (earlier) to right (later) by their RNA velocity positions in Fig. 4A. Error bars show SEM. (**F**) Expression of *Pou5f1* in WT and *Nipbl^+/−^* embryos from LB to CC stages. Error bars show SEM. *P* values from *t* test. (**G**) Monoclonal generation of *Nipbl*^Flrt/+^ (WT) and *Nipbl*^Flex/+^ (*Nipbl^+/−^*) ESCs. (**H**) Expression of *Nipbl*, *Nanog*, and *Pou5f1* in WT and *Nipbl*^+/−^ ESCs as measured by RT-qPCR and normalized to the housekeeping gene, *Rpl4*. Error bars show SEM. *P* values from *t* test.

To uncover developmental processes that might be the most broadly affected in *Nipbl^+/−^* embryos, we used the large, significant, changes in gene expression (greater than twofold up or down) as input data for STRING (*108*), a database and algorithm that constructs a network of potential gene interactions (data S35), in this case, limiting predictions to those informed by experiments demonstrating coexpression or protein-protein interactions. The outcome of this analysis (Fig. 7, A and B) revealed that many genes with substantial expression changes in *Nipbl^+/−^* embryos are predicted to interact either directly or indirectly with *Nanog*. *Nanog* itself was overexpressed across all three germ layers in *Nipbl^+/−^* embryos, with a more pronounced overexpression, exceeding twofold, in the mesoderm and endoderm (Fig. 7B).

*Nanog*, which encodes a transcription factor, is notably expressed at two junctures during mouse embryonic development. Initially, it appears at the blastocyst stage, where it plays an essential role in sustaining the pluripotency of the inner cell mass cells (*109*). Subsequently, during gastrulation, *Nanog* expression temporarily surges, only to be silenced as cells transition out of the primitive streak (*110*). This expression pattern could be observed directly in our data: In Fig. 7C, we ordered mesodermal cell populations from LB-stage embryos by their stage in gastrulation, as inferred from their RNA velocity positions (Fig. 4A). In WT NMPs, *Nanog* is expressed at a relatively high level. As cells move through successive stages of gastrulation within WT embryos, *Nanog*’s expression exhibits a consistent decline (Fig. 7C and data S36), reaching near zero in cells of the FHF and SHF. An exception to this trend occurs in PGCs, which are known to maintain elevated *Nanog* expression (*111*). In contrast, in *Nipbl^+/−^* embryos (Fig. 7C), *Nanog* expression remained elevated across all cell populations. Although expression still falls after the NMP stage in *Nipbl^+/−^* cells, *Nanog* never falls to WT levels. This is evident even in PGCs. Moreover, the overexpression of *Nanog* in *Nipbl^+/−^* embryos is sustained up to the CC stage (Fig. 7D and table S10), where it reaches a 10-fold increase over WT levels. These observations indicate that *Nipbl* haploinsufficiency leads to a marked failure of *Nanog* down-regulation.

Another transcription factor that plays a role in maintaining pluripotency is *Pou5f1* (*Oct4*). From studies in embryonic stem cells (ESCs), it is known that *Pou5f1* promotes the expression of *Nanog* and, conversely, *Nanog* promotes the expression of *Pou5f1* (*112*). Given this, we were curious about whether *Nipbl*^+/−^ embryos might also overexpress of *Pou5f1*. Despite the fact that *Pou5f1* is more highly expressed than *Nanog*, we did not observe any statistically significant difference in *Pou5f1* expression across mesodermal cell types or throughout LB-stage *Nipbl^+/−^* embryos (Fig. 7, E and F, data S37, and table S11). However, a more than twofold elevation in *Pou5f1* expression was detected in *Nipbl^+/−^* embryos at CC stage (Fig. 7F).

We sought to determine whether elevated *Nanog* expression in *Nipbl^+/−^* embryos during gastrulation might reflect some sort of nonspecific overactivity of the *Nanog* gene. To investigate this hypothesis, we looked at *Nanog* expression in WT and *Nipbl^+/−^* ESCs, as ESCs are known to express *Nanog* (as do to the blastocyst inner cell mass cells from which ESCs are derived). We generated WT and *Nipbl^+/−^* ESCs by treating *Nipbl*^Flex/+^ (*Nipbl^+/−^*) ESCs with Flp recombinase (which inverts a gene trap in *Nipbl*-Flex ESCs in such a way that reverses gene trapping) to produce *Nipbl*^Flrt/+^ (WT) ESCs (Fig. 7G) (*24*). We assessed *Nanog* expression in nine independent clones from each genotype, using reverse transcription quantitative polymerase chain reaction (RT-qPCR). We observed no discernible difference in *Nanog* expression between *Nipbl^+/−^* and WT ESCs, a pattern that was also observed for *Pou5f1* (Fig. 7H and data S38). As a control, we showed that *Nipbl^+/−^* ESCs exhibit the expected reduced expression of *Nipbl*, as also seen in *Nipbl^+/−^* embryos. Collectively, these findings support the conclusion that the overexpression of *Nanog* in *Nipbl^+/−^* LB-stage and CC-stage embryos is result of a specific failure to appropriately suppress *Nanog* following gastrulation, rather than unusually elevated *Nanog* expression persisting from early embryogenesis.

### *Nanog* overexpression accounts for many of the gene expression changes in *Nipbl^+/−^* mice

As a critical regulator of pluripotency in early embryonic development, *Nanog* directly influences the expression of a multitude of other genes. This relationship prompted us to ask how many of the gene expression differences in *Nipbl^+/−^* embryos might be attributable to the overexpression of *Nanog*. To address this question, we took advantage of data from a recent study, by Tiana *et al.* (*113*), in which mice were engineered to express *Nanog* under doxycycline (Dox)–inducible control. In that study, bulk RNA sequencing was performed on untreated (*Nanog* Dox–) and Dox-treated (*Nanog* Dox+) embryos at stages E7.5 and E9.5, with E7.5 corresponding closely to the LB-stage embryos analyzed here. The E7.5 *Nanog* Dox+ embryos that were sequenced had been treated with doxycycline from E4.5 to E7.5, while E9.5 *Nanog* Dox+ embryos were treated with doxycycline from E6.5 to E9.5. DGEA, performed in that study, found hundreds of gene expression changes between *Nanog* Dox– and *Nanog* Dox+ embryos at both stages (*113*). We subsequently analyzed the results of their analysis in four ways.

**Fig. 8.**
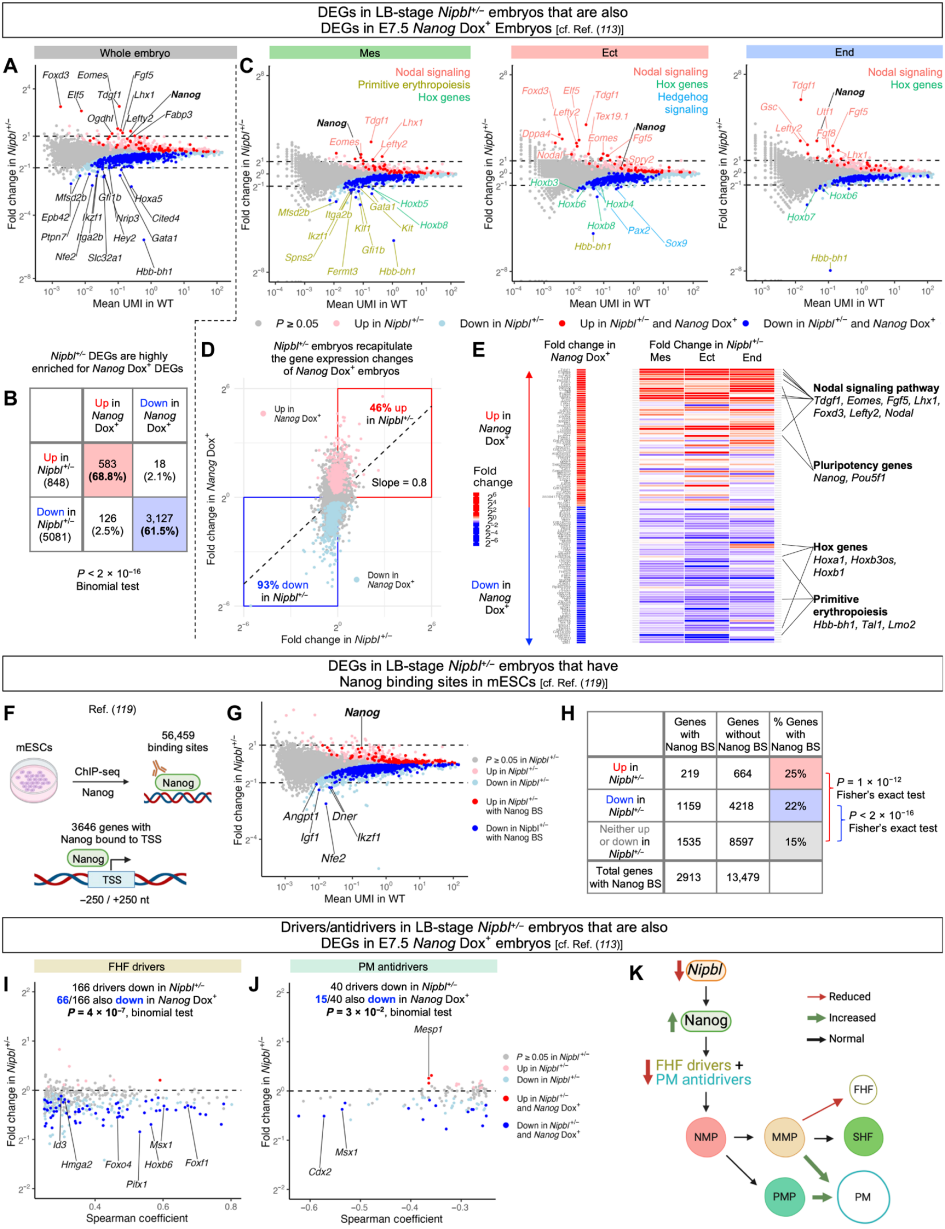
LB-stage *Nipbl^+/−^* mice replicate the gene expression changes of *Nanog* overexpression. (**A**) Fold change in expression of DEGs in LB-stage *Nipbl*^+/−^ embryos (*Q* < 0.05, Mann-Whitney *U* test) that are also DEGs in E7.5 *Nanog* Dox+ embryos (*Q* < 0.05, *t* test) (*113*). (**B**) Percentage of DEGs in LB-stage *Nipbl*^+/−^ embryos that are also DEGs in E7.5 *Nanog* Dox+ embryos (*113*). (**C**) Fold change in expression of DEGs in the germ layers of LB-stage *Nipbl*^+/−^ embryos that are also DEGs in E7.5 *Nanog* Dox+ embryos (*113*). (**D**) Fold change in expression of genes in LB-stage *Nipbl*^+/−^ embryos versus their fold change in E7.5 *Nanog* Dox+ embryos. DEGs in E7.5 *Nanog* Dox+ embryos (*113*) are colored pink and light blue. (**E**) Heatmap of the fold change in expression of DEGs from E7.5 *Nanog* Dox+ embryos (lowest *Q* values from *t* test) (*113*) in the germ layers of LB-stage *Nipbl*^+/−^ embryos. (**F**) ChIP sequencing for Nanog in mESCs from (*119*) identified 3646 genes with one or more Nanog binding sites within ±250 nt of their TSSs. (**G**) Fold change in expression of DEGs in LB-stage *Nipbl*^+/−^ embryos with one or more Nanog binding sites (BS) from (*119*). (**H**) Percentage of DEGs in LB-stage *Nipbl*^+/−^ embryos with one or more Nanog binding sites from (*119*). Fold change in expression of DEGs in mesoderm cells of (**I**) FHF and (**J**) PM lineages of LB-stage *Nipbl*^+/−^ embryos that are also DEGs in E7.5 *Nanog* Dox+ embryos (*113*). Transcription factor genes are labeled. (**K**) Reduction of *Nipbl* levels leads to the up-regulation of *Nanog* in LB-stage *Nipbl^+/−^* embryos and down-regulation of FHF drivers and PM antidrivers, resulting in the misallocation of mesoderm cells to PM at the expense of FHF.

First, we plotted the fold change in expression of genes in *Nipbl^+/−^* embryos, as a whole, from that of WT embryos, concentrating on LB stage (Fig. 8A and data S39). (CC-stage comparisons are shown in fig. S15, A and B, and data S40.) In Fig. 8A, genes significantly overexpressed in *Nipbl*^+/−^ embryos are colored pink and those significantly underexpressed, light blue. DEGs in *Nipbl^+/−^* embryos that are also significantly overexpressed or underexpressed in E7.5 *Nanog* Dox+ embryos are colored red and blue, respectively. The results are summarized in Fig. 8B. At LB stage, 68.8% of overexpressed and 61.5% of underexpressed genes in *Nipbl^+/−^* embryos were also overexpressed or underexpressed, respectively, in E7.5 *Nanog* Dox+ embryos. A binomial test confirmed that *Nanog* Dox+ DEGs are highly overrepresented in *Nipbl^+/−^* embryos (Fig. 8B).

Second, we again plotted the fold change in expression of genes in LB-stage *Nipbl^+/−^* embryos from that of WT embryos, but this time separately by germ layer, rather than across whole embryos (Fig. 8C). Genes colored red and blue are DEGs in *Nipbl^+/−^* embryos that are also overexpressed or underexpressed in *Nanog* Dox+ embryos. When we manually curated genes that were expressed with more than twofold change (Fig. 8C), we found that all germ layers showed up-regulation of genes involved with Nodal signaling (*Tdgf1*, *Lefty2*, *Eomes*, *Lhx1*, and *Ffg5*) (*96**–**99*, *114*) and down-regulation of *Hox* genes (*Hoxb8 and Hoxb6*). In addition, the mesoderm of *Nipbl^+/−^* embryos underexpressed genes associated with primitive erythropoiesis (*Hbb-bh1*, *Klf1*, and *Gata1*) (*74*, *100*) and hedgehog signaling (*Pax2* and *Sox9*) (*103*, *104*). (CC-stage gene expression changes are discussed in fig. S15C.) These findings are consistent with the idea that many of the largest changes in gene expression that occur in *Nipbl^+/−^* embryos, as well as the developmental pathways they regulate, could be attributable to overexpression of *Nanog*.

Third, we analyzed how closely *Nipbl^+/−^* embryos mirrored the gene expression alterations of *Nanog* Dox+ embryos in terms of magnitude and direction. To do this, we generated a plot illustrating the fold changes in expression of individual genes in *Nipbl^+/−^* embryos compared to their fold change in *Nanog* Dox+ embryos (Fig. 8D). We colored genes that were significantly overexpressed in *Nanog* Dox+ embryos pink and those significantly underexpressed light blue. In addition, we calculated the regression line depicting the relationship between fold changes in gene expression in *Nipbl^+/−^* versus *Nanog* Dox+ embryos. At LB stage, the slope of the regression line between *Nipbl^+/−^* and E7.5 *Nanog* Dox+ embryos was 0.8, implying that, on average, the fold changes in gene expression in *Nipbl^+/−^* embryos are quantitatively similar to those in *Nanog* Dox+ embryos (Fig. 8D). Figure 8D also depicts that, at LB stage, *Nipbl^+/−^* embryos up-regulate 46% of the same genes that were overexpressed in E7.5 *Nanog* Dox+ embryos and down-regulate 93% of the same genes that were underexpressed in E7.5 *Nanog* Dox+ embryos. (Similar analysis was performed at CC stage and is described in fig. S15D.) These findings highlight a remarkable similarity in gene expression changes between *Nipbl^+/−^* and *Nanog* Dox+ embryos.

Fourth, we examined the magnitudes of fold changes in individual gene expression in *Nipbl^+/−^* embryos and *Nanog* Dox+ embryos, focusing on the 50 most highly up- and down-regulated genes in *Nanog* Dox+ embryos. As shown in Fig. 8E, we categorized genes into two groups at each stage: those overexpressed in *Nanog* Dox+ embryos (ranked from top to middle) and those underexpressed in *Nanog* Dox+ embryos (ranked from bottom to middle), ordering them by ascending *Q* value. Next, we constructed a heatmap that visually captures the fold changes in gene expression within *Nanog* Dox+ whole embryos, contrasting them with the corresponding fold changes in the individual germ layers of *Nipbl^+/−^* embryos (Fig. 8E). E7.5 *Nanog* Dox+ embryos overexpressed genes linked with the Nodal signaling pathway (*Tdgf1*, *Eomes*, *Fgf5*, *Lhx1*, *Foxd3*, *Lefty2*, and *Nodal*) (*96**–**99*, *114**–**116*) and those integral to maintaining pluripotency (*Nanog* and *Pou5f1*) (Fig. 8E) (*112*). Concurrently, they underexpressed genes associated with primitive erythropoiesis (*Hbb-bh1*, *Tal1*, and *Lmo2*) (*117*, *118*) and specific *Hox* genes (*Hoxa1*, *Hoxb3os*, and *Hoxb1*). Notably, this pattern of fold changes was mirrored, to a remarkable degree, in the germ layers of LB-stage *Nipbl^+/−^* embryos (Fig. 8E). (The corresponding analysis for CC-stage embryos is given in fig. S15E.)

### DEGs from *Nipbl^+/−^* embryos are enriched for nanog binding sites

Given the large overlap between the gene expression changes observed in LB-stage *Nipbl*^+/−^ embryos and E7.5 *Nanog* Dox+ embryos, we wondered how many DEGs in *Nipbl^+/−^* embryos might be direct targets of Nanog. Although genome-wide patterns of Nanog binding have not been characterized for embryos at this stage, many groups have used chromatin immunoprecipitation sequencing (ChIP-seq) to define Nanog binding sites in mouse ESCs (mESCs). We turned to a recent study (*119*), in which Avsec *et al.* captured 52,456 Nanog binding sites in the genome of mESCs, and reported that 3645 genes showed one or more Nanog binding sites located within 250 nucleotides (nt) on either side of their transcription start sites (TSSs) (Fig. 8F). On comparing these genes with those up- and down-regulated in LB-stage *Nipbl^+/−^* embryos, we found that 25% of the overexpressed genes and 22% of the underexpressed genes match those genes with Nanog binding sites in mESCs (Fig. 8, G and H). This represented significant enrichment for Nanog binding sites in differentially versus non-DEGs (*P* < 1 × 10^−12^ and *P* < 2 × 10^−16^, Fisher’s exact tests). These data suggest that a substantial portion of DEGs in LB-stage *Nipbl^+/−^* embryos could indeed be direct targets of Nanog.

### *Nanog* overexpression may account for the down-regulation of FHF drivers in *Nipbl^+/−^* mice

As shown above in Fig. 3K and fig. S13, FHF drivers and PM antidrivers are down-regulated in LB-stage *Nipbl^+/−^* mice. Given the large overlap between down-regulated genes in LB-stage *Nipbl^+/−^* embryos and down-regulated genes in E7.5 *Nanog* Dox+ embryos (Fig. 8, B and D), we wondered how many of the down-regulated FHF drivers and PM antidrivers in *Nipbl^+/−^* embryos could have their changes in expression attributed to the overexpression of *Nanog*. To answer this question, we compared the FHF drivers and PM antidrivers down-regulated in LB-stage *Nipbl^+/−^* embryos to those genes exhibiting down-regulation in E7.5 *Nanog* Dox+ embryos. We found that ~40% of the FHF drivers and 38% of the PM antidrivers that were down-regulated in *Nipbl^+/−^* embryos were also down-regulated in E7.5 *Nanog* Dox+ embryos (Fig. 8, I and J). This raises the possibility that overexpression of *Nanog* leads to the down-regulation of FHF drivers and PM antidrivers and that this results in the misallocation of mesoderm cells to a PM fate at the expense of FHF (Figs. 3 and 8K).

### *Nipbl^+/−^* mice exhibit delayed expression of anterior *Hox* genes

**Fig. 9.**
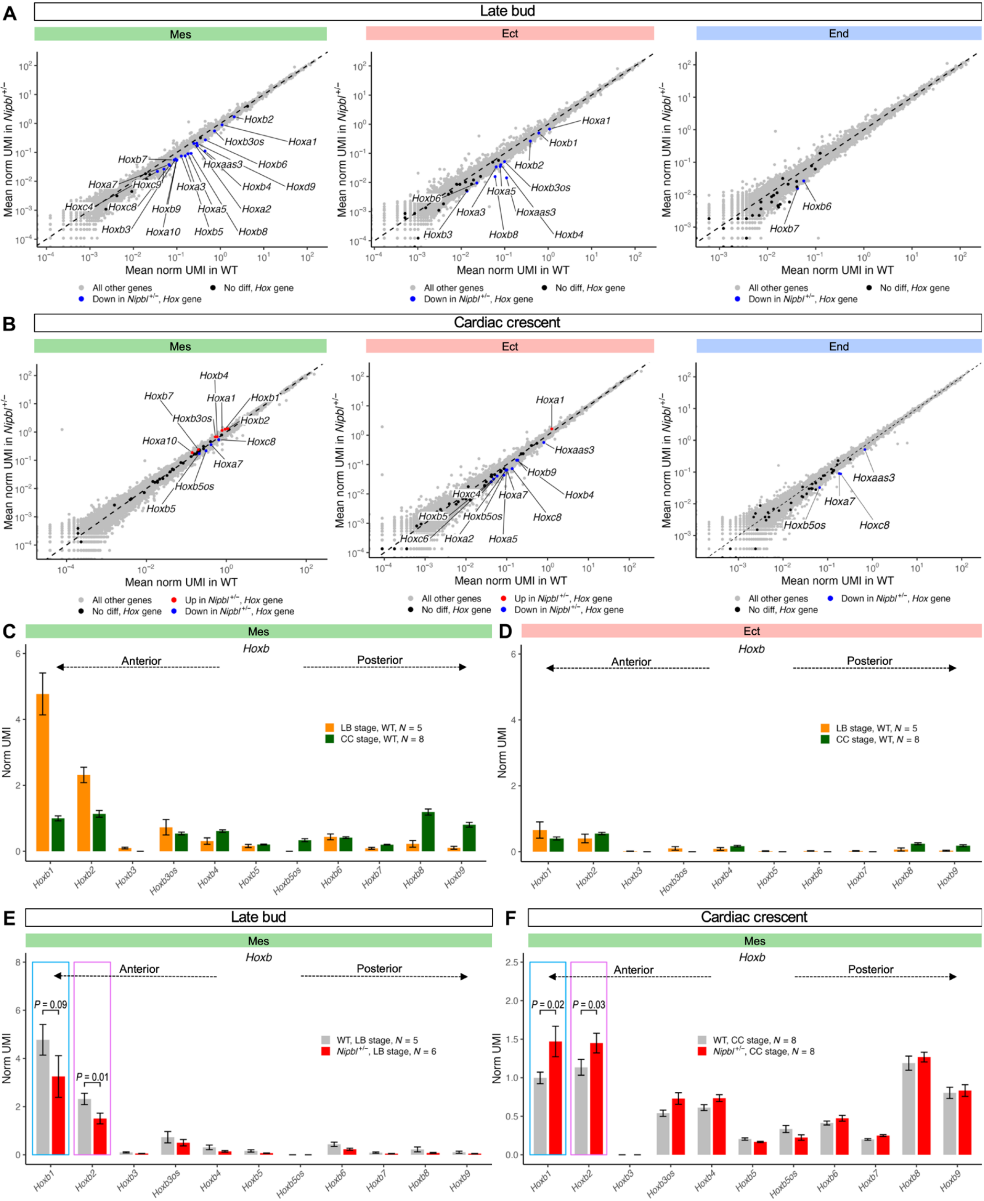
*Nipbl*^+/−^ mice show a delay in the expression of anterior *Hox* genes. Expression of *Hox* genes in germ layers of WT and *Nipbl*^+/−^ embryos at (**A**) LB and (**B**) CC stages. Expression of *Hoxb* genes in mesoderm (**C**) and ectoderm (**D**) of WT embryos from LB to CC stages ordered from left (5′) to right (3′) by their chromosomal position. Expression of *Hoxb* genes in mesoderm of WT embryos at (**E**) LB and (**F**) CC stages ordered from left (5′) to right (3′) by their chromosomal position.

We noticed that LB-stage *Nipbl^+/−^* embryos exhibited a significant underexpression, exceeding twofold, in *Hox* genes across all germ layers, especially genes within the *Hoxb* cluster (Figs. 6D and 8C). As *Hox* genes are integral to spatial patterning in development, we compared the expression of all *Hox* genes in *Nipbl^+/−^* embryos with their WT counterparts. The results, shown in Fig. 9 (A and B), revealed extensive misexpression at both LB and CC stages. At LB stage, any *Hox* gene that was significantly differentially expressed was underexpressed in *Nipbl^+/−^* embryos in every germ layer where expression was detectable (Fig. 9A). At CC stage, we observed a trend of underexpression across all germ layers for the majority of misexpressed *Hox* genes (Fig. 9B), but exceptions were found in the mesoderm and ectoderm, where a select few *Hox* genes were overexpressed. These included *Hoxb1* and *Hoxb2* in the mesoderm and *Hoxa1* in the ectoderm, all of which are characterized as anterior *Hox* genes.

*Hox* genes are organized into chromosomal clusters that turn on expression in a wave-like temporal pattern, producing highly structured and sequential activation during development. Specifically, genes at the 5′ end of the cluster, known as anterior *Hox* genes, are activated early in development and progressively silenced as genes further forward the 3′ end (posterior *Hox* genes) are turned on (*120*). To investigate whether our data reflected this known pattern, we examined the *Hoxb* genes, as they were the most prominently expressed *Hox* genes at both LB and CC stages. After ordering them according to their chromosomal positions, we plotted their expression within the mesoderm and ectoderm of WT embryos at the corresponding developmental stages. In the mesoderm of LB-stage embryos, anterior *Hoxb* genes were actively expressed, while posterior *Hoxb* genes were only faintly detectable, if at all (Fig. 9C and data S44). At CC stage, the expression of anterior *Hoxb* genes decreased from their LB-stage levels, accompanied by the emergence of posterior *Hoxb* gene expression (Fig. 9C). This dynamic shift in expression aligns closely with the established understanding of *Hox* gene regulation during development. In contrast, the ectoderm exhibited a less pronounced pattern, with both anterior and posterior *Hoxb* genes expressing at minimal levels (Fig. 9D and data S45). No discernible difference in the expression of anterior *Hoxb* genes was detected in the ectoderm between LB- and CC-stage embryos (Fig. 9D).

Next, we looked at the expression of *Hoxb* genes in the mesoderm of *Nipbl^+/−^* embryos. As in WT embryos, anterior *Hoxb* genes were actively expressed at LB stage, while posterior *Hoxb* genes were silent (Fig. 9E). However, *Hoxb1* and *Hoxb2* were expressed at levels that were conspicuously lower than in WT (Fig. 9E). At CC stage, *Nipbl^+/−^* embryos reflected the general trend found in WT embryos, with anterior *Hoxb* genes waning in expression as the posterior *Hoxb* genes began their ascent (Fig. 9F, note axis scale). The two most anterior Hox genes, *Hoxb1* and *Hoxb2*, did not decline as much, proportionally, as they did in WT embryos, with the outcome being that, by CC-stage, expression of *Hoxb1* and *Hoxb2* in *Nipbl^+/−^* embryos was actually higher than that in WT (Fig. 9F). These results collectively provide evidence for a temporal disruption in the *Hox* gene expression program in *Nipbl^+/−^* embryos. Specifically, *Hox* genes appear to be delayed in initiating expression at the LB stage and similarly tardy in becoming suppressed at the CC stage.

### *Nipbl^+/−^* mice show anteriorization of thoracic vertebrae, with left-right asymmetry

**Fig. 10.**
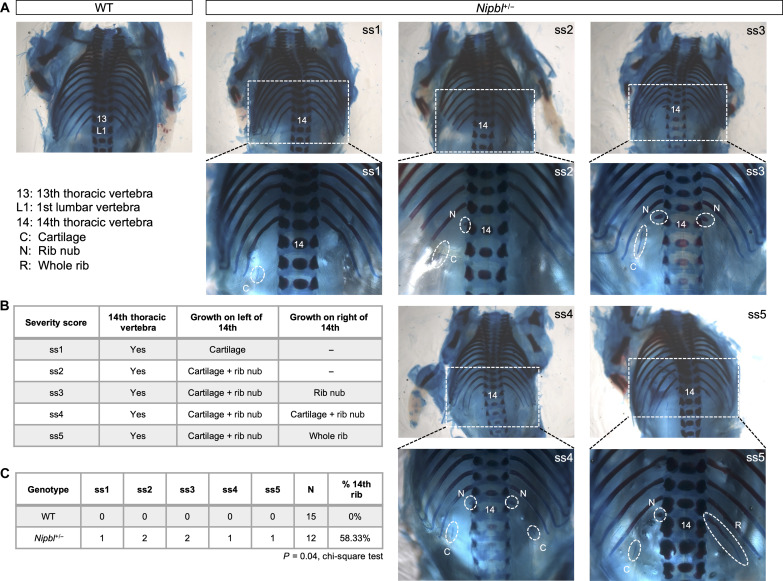
*Nipbl^+/−^* mice show anteriorization of thoracic vertebrae with left-right asymmetry. (**A**) Dorsal view of bone (Alizarin red) and cartilage (Alcian blue) stained rib cage of E18.5 WT and *Nipbl*^+/−^ embryos. WT embryos only show 13 ribs. *Nipbl*^+/−^ embryos show incomplete asymmetric growth of 14th rib. ss1 to ss5 refers to severity score in (B). 13, 13th thoracic vertebra; L1, first lumbar vertebra; C, cartilage; N, rib nub; R, whole rib. (**B**) Table categorizing range of incomplete asymmetric growth of 14th rib observed in E18.5 *Nipbl*^+/−^ embryos and ranking them by their severity (low, ss1; high, ss5). (**C**) Table quantifying numbers of WT and *Nipbl*^+/−^ embryos in which incomplete asymmetric growth of 14th rib was observed per severity score. *P* value from chi-square test.

A previous study (*121*) showed that a knockout of *Hoxb1-Hoxb9* in mice led to the anteriorization of the axial skeleton, characterized by an increased number of thoracic vertebrae with ribs. Since *Nipbl^+/−^* mice underexpress *Hox* genes and show delayed regulation of anterior *Hox* genes, we were curious if similar anteriorizations could be detected in *Nipbl^+/−^* mice (Fig. 9). To explore this, we analyzed the vertebrae and ribs of 15 WT and 12 littermate *Nipbl^+/−^* embryos at E18.5, using Alizarin red and Alcian blue stains. In WT embryos, we confirmed the presence of the normal count of 13 ribs (Fig. 10A). In contrast, many *Nipbl^+/−^* embryos displayed 14 thoracic vertebrae, the 14th of which bore a range of rib growths, often displaying left-right asymmetry (Fig. 10A). We classified the growths of these 14th vertebrae based on severity, ranking them from low (ss1) to high (ss5), and documented left-right differences (Fig. 7B). Three of seven *Nipbl^+/−^* embryos displayed partial growth of a 14th rib on the left side only (S1 and S2). These growths were either cartilage only or disconnected formation of rib nub with cartilage but never any whole ribs. Four of seven *Nipbl^+/−^* embryos showed bilateral growth (S3 to S5). Partial growth occurred on both the left and right sides, but complete formation a whole rib only ever occurred, in one instance, on the right side (S5). In total, 58% of E18.5 *Nipbl^+/−^* embryos demonstrated some form of rib growth from a 14th thoracic vertebra (Fig. 10C). These findings suggest that anteriorization of the axial vertebrae does occur in *Nipbl^+/−^* embryos, possibly as a result of delayed activation (and inactivation) of anterior *Hox* gene expression.

## DISCUSSION

The *Nipbl^+/−^* mouse, a model of the multisystem birth defect syndrome, CdLS (*122*), offers a distinct lens through which to explore the genetic origins of birth defects. *Nipbl^+/−^* mice display many of the same birth defects observed in human CdLS, and a key feature of *Nipbl* deficiency across various organisms is quantitative alterations in gene expression, including both up-regulation and down-regulation, affecting at least hundreds of genes in every tissue (*18*, *24*, *123*). Previous research into animal models of *Nipbl* deficiency suggested that the root causes of birth defects in CdLS likely manifest during a period when progenitor cell populations are being formed for all major tissue and organ lineages (*22*, *24*). Therefore, to elucidate how early, cell type–specific changes in gene expression might contribute to the onset of birth defects, we used scRNA-seq to conduct a comparative analysis between *Nipbl^+/−^* embryos and their WT littermates at key developmental stages, focusing on the conclusion of gastrulation, LB stage (approximately E7.5) to early CC stage (approximately E7.75) (*25*).

### Changes in the sizes of different mesodermal cell populations in *Nipbl*^+/−^ embryos foreshadow pathological changes in tissue composition and patterning in CdLS

In Fig. 1, we showed that *Nipbl^+/−^* embryos do not lack any cell populations found in WT embryos. However, LB-stage *Nipbl^+/−^* embryos have fewer total mesoderm cells and, within the mesoderm, have fewer PEs and FHF cells and more PM cells (Fig. 2). These changes, while not always large, are likely to be of physiological importance. For example, the observation that PEs are reduced in number is consistent with defects in blood formation and circulation observed in *Nipbl*-morphant zebrafish (*22*) and thrombocytopenia in CdLS (*124*).

In addition, the reduction in FHF cell number could contribute to the presence of CHDs in *Nipbl^+/−^* mice and individuals with CdLS (*24*, *125*). In a prior study in *Nipbl*^+/−^ mice, most CHDs observed were in heart regions (right ventricle and atrial/ventricular septa) considered to be derivatives of the SHF (*24*). Although FHF cells are not thought to give rise directly to “SHF structures,” they likely exert indirect effects on the development of SHF derivatives. This echoes the conclusions of Santos *et al.* (*24*), who found that complex interactions between different cell types (even between noncardiogenic and cardiogenic cell types) influence the incidence of CHDs in *Nipbl*^+/−^ mice.

The increased number of PM cells at LB stage is intriguing, as it correlates with observed defects in PM derivatives, including the axial skeleton (Fig. 10) and limb digits in *Nipbl^+/−^* mice (*126*). Notably, both such deficits are features of CdLS (*127*). How an overabundance of PM cells could contribute to limb defects is not obvious. Nevertheless, these findings suggest that birth defects in *Nipbl*-deficient organisms arise not only from the absence of specific progenitor cell populations but also from progenitor cell misallocation events that alter the relative proportions of cell types.

### Progenitor cell misallocation in *Nipbl^+/−^* correlates with changes in the expression of cell fate driver genes

*Nipbl^+/−^* embryos at LB stage did not exhibit global changes in apoptotic activity, cell proliferation, developmental timing, or overall lineage trajectories (Figs. 3 and 4 and figs. S11 and S12). However, we produced evidence that nascent mesoderm cells in *Nipbl^+/−^* embryos differentiated more frequently into PM than FHF, compared to WT (Fig. 4, C to I). This led us to conclude that misallocation of mesoderm cells in *Nipbl^+/−^* embryos is driven by alterations in early cell fate choices that occur along the main pathways of otherwise unaltered lineage trajectories. These findings suggest that some types of structural birth defects, such as heart defects, arise from quantitative alterations to cell fate determination, rather than a complete disruption of an entire lineage pathway.

In Fig. 5, we presented evidence that reduced differentiation of mesoderm cells into FHF in *Nipbl^+/−^* embryos could be attributed to the underexpression of genes predicted to drive FHF differentiation. Underexpressed FHF driver genes show strong enrichment for genes associated with EMT (Fig. 5, J and K). EMT not only occurs during the emergence of mesenchymal mesoderm and FHF cells during gastrulation but also plays a role in the contribution of later cell lineages (SHF cells, endocardial cells, and neural crest cells) to various cardiac structures. SHF cells undergo EMT as they participate in the elongation of the heart tube and outflow tract (*128*), and endocardial cells undergo EMT as they form structures such as the cardiac cushion (*129*). Later in development, migratory neural crest cells, which arise from neural epithelium by EMT, contribute to the aorticopulmonary septum and parts of the outflow tract (*130*). Together, these observations suggest that dysregulation of EMT genes may play an important role in the development of heart defects in *Nipbl^+/−^* mice.

### Is *Nanog* overexpression responsible for gene expression changes in *Nipbl*^+/−^ embryos?

In contrast to our prior studies of *Nipbl^+/−^* mice, in which differences in gene expression were invariably found to be small (less than twofold) (*18*), the present study demonstrated that, in gastrulation stage embryos, some genes show much larger shifts in expression (as high as eightfold) (Figs. 6D and 8H). One of the largest of such changes was in *Nanog*, which displayed overexpression in LB-stage *Nipbl^+/−^* embryos and which persisted to CC stage (Figs. 7 and 8). Using data from Tiana *et al*. (*113*), we demonstrated that many of the gene expression changes in *Nipbl^+/−^* embryos mirror those seen in embryos with induced *Nanog* overexpression (Fig. 8, A to E, and fig. S15, A to E). Moreover, many *Nipbl^+/−^* DEGs are likely to be direct Nanog targets, as the corresponding genes display enrichment for Nanog binding sites (Fig. 8, F and H). Comparisons of our data with those of Tiana *et al.* (*113*) also suggest that misallocation of mesoderm cells to a PM fate at the expense of FHF, resulting from down-regulation of FHF drivers/PM antidrivers, may be a consequence of *Nanog* overexpression (Figs. 3 and 8K).

A question that arises from these observations is whether all gene expression alterations observed in *Nipbl*^+/−^ embryos might be due to *Nanog* overexpression. For a number of reasons, we do not think that this is the case. First, for the largest gene expression shifts (greater than twofold over- or underexpressed), only 39% at LB stage and 18% at CC stage were misexpressed in the same direction as DEGs in embryos in which *Nanog* was overexpressed (Fig. 8 and fig. S15) (*113*). Second, although *Nanog* continues to be overexpressed in *Nipbl^+/−^* embryos from LB to CC stages, the magnitude of its expression decreases substantially by CC stage (Fig. 7). Third, *Nipbl* haploinsufficiency itself causes significant, large expression changes in other genes not implicated as consequences of *Nanog* overexpression. For instance, we observed reduced expression of genes linked to the Notch signaling pathway in the mesoderm and increased expression of adult hemoglobin genes in the endoderm (Fig. 6). Therefore, we conclude that the extensive gene expression changes in *Nipbl^+/−^* embryos are unlikely to be due to *Nanog* overexpression alone.

### Skeletal anomalies in *Nipbl^+/−^* mice reflect earlier delays in anterior *Hox* gene expression

In Figs. 4D and 8G, we showed that large decreases in *Hox* gene expression occur across all germ layers of LB-stage *Nipbl^+/−^* embryos. While only a subset of *Hox* genes is underexpressed by more than twofold, many *Hox* genes across all germ layers show small (less than twofold) but significant decreases in expression (Fig. 9A). We also provide evidence for a temporal disturbance in anterior *Hox* gene expression in *Nipbl^+/−^* embryos, whereby *Nipbl* haploinsufficiency postpones the onset of anterior *Hox* gene expression at LB stage and delays their subsequent deactivation at CC stage (Fig. 9, C to F). These shifts in *Hox* gene expression dynamics mirror those in a previous study of *nipbl*-morphant zebrafish, in which genome location–specific misexpression of *hox* genes was also observed (*131*). In Fig. 10, we showed that anteriorization of thoracic vertebrae occurs in E18.5 *Nipbl^+/−^* mice (Fig. 9). These alterations in axial skeletal development parallel skeletal anomalies that have been identified in individuals with CdLS, including fused, absent, or misshapen ribs (*132*). Since *Nipbl* deficiency is the dominant form of CdLS, it is interesting to speculate that these skeletal anomalies might originate from underexpression of and delayed anterior *Hox* gene activity during gastrulation.

### Overexpression of Nodal signaling pathway genes may contribute to left-right patterning defects in CdLS

In Fig. 4D, we showed that *Nipbl^+/−^* embryos at LB stage show large overexpression of genes associated with the Nodal signaling pathway across all germ layers. Nodal signaling, pivotal for left-right patterning (*133*), ensures the appropriate positioning and morphogenesis of the musculoskeletal system and internal organs (*134*). In Fig. 10, we showed that growth of a 14th vertebra in E18.5 *Nipbl^+/−^* embryos displayed left-right asymmetry, with the growth of a whole 14th rib only occurring on the right side of the embryo. These patterning alterations are consistent with the heightened incidence of right-sided skeletal anomalies in CdLS (*127*). Changes in left-right patterning are also consistently associated with intestinal malrotation and some types of cardiac anomalies observed in *Nipbl^+/−^* mice, *nipbl*-morphant zebrafish, and CdLS (*18*, *24*, *135*). These data suggest that left-right patterning defects in CdLS might be due to overexpression of Nodal signaling genes as early as gastrulation.

### Gene expression changes observed in early *Nipbl^+/−^* embryos are likely driven by Nipbl’s effects on chromatin topology

The results of this study, together with prior studies of mouse and zebrafish models, reinforce the notion that the chromosomal location of genes is an important factor in their sensitivity to variations in *Nipbl* levels. For example, our prior studies of *Nipbl^+/−^* embryonic brain demonstrated that the largest expression changes among genes in the 22-gene protocadherin beta (*Pcdhb*) cluster were found in those genes situated at the 5′ and 3′ ends of the cluster (*18*), where CTCF sites are located (*136*). In developing pectoral fins of *nipbl*-morphant zebrafish, *hox* genes located near the 3′ end of three separate *hox* clusters (*hoxa*, *hoxc*, and *hoxd*) show a position-dependent pattern of overexpression (*131*). Similarly, in the present study, we showed that anterior *Hoxb* genes—located at the 3′ end of the *Hoxb* cluster—are preferentially underexpressed in the mesoderm of *Nipbl^+/−^* embryos at gastrulation (Fig. 8E). Thus, we consistently observe a strong influence of chromosomal location on gene sensitivity to *Nipbl* levels, particularly within gene clusters, and this influence is already apparent at gastrulation.

Recent studies suggest that changes in gene expression due to *Nipbl* deficiency may be the result of global alterations to chromosomal structure and organization. For instance, marked reduction of *Nipbl* expression in mouse hepatocytes resulted in genome-wide depletion of topologically associated domains (TADs) and Hi-C interaction peaks (*137*), both of which require chromatin looping (*138*). Recent studies have demonstrated that both the formation and rate of chromatin loop extrusion were reduced in vitro when a CdLS pathogenic mutation was introduced into *NIPBL* (*139*).

Chromatin looping within TADs is thought to bring distant cis-regulatory elements, such as enhancers, into proximity with target promoters. Nipbl has been reported to preferentially bind to enhancers and promoters (*140*), and ChIP-seq studies show reduced enhancer-promoter interactions in *Nipbl*^+/−^ mouse embryonic fibroblasts (*141*). Since chromatin looping is essential for gene activation, impaired loop extrusion as a consequence of reduced Nipbl levels may provide an explanation for why *Nipbl* haploinsufficiency causes more genes to be down-regulated than up-regulated in gastrula-stage mouse embryos (e.g., >80% of mesodermal genes; Fig. 6A), a trend that has been observed in studies of other tissues (*141*).

In light of these observations, it is interesting that overexpression of *Nanog* and its target genes emerged from the present study as particularly likely to play important roles in causing CdLS phenotypes. During normal gastrulation, *Nanog* expression falls to very low levels in most cell types, so what occurs in *Nipbl*^+/−^ embryos is perhaps best viewed as a failure of gene repression. Although it is possible that *Nipbl*^+/−^ haploinsufficiency leads to down-regulation of a gene encoding a *Nanog* repressor, no obvious candidate stands out within the gene expression changes identified in the present study. We speculate, instead, that *Nipbl*-sensitive loop formation is required for the silencing of *Nanog* gene expression during gastrulation and, thus, that changes in chromatin architecture play a key role in timing the termination of this critical embryonic event.

## MATERIALS AND METHODS

### Statement on care and use of animals

All animals were handled in accordance with approved procedures as defined by the National Institutes of Health, and all animal work was approved by the Institutional Animal Care and Use Committee of the University of California, Irvine. For collection of mouse tissues, pregnant dams were humanely killed by CO_2_ anesthesia, followed by cervical dislocation.

### Generation of WT and *Nipbl*^+/−^ mice

WT and *Nipbl*^+/−^ mouse littermates were generated by mating *Nanog*^Cre/+^ mice (*26*) and *Nipbl*^Flox/Flox^ mice (*24*). *Nipbl*^Flox/Flox^ mice have an inverted gene trap cassette encoding β-*geo* that is flanked by *Cre* recombinase target sites in intron 1 of *Nipbl* alleles (Fig. 1A) (*24*). In this inverted orientation, referred herein as Flox, there is no trapping of the *Nipbl* gene, and *Nipbl* is expressed normally. However, when this cassette is exposed to Cre recombinase, the gene trap cassette gets inverted into a noninverted orientation that we call FIN (Fig. 1A). In this noninverted orientation, trapping of the *Nipbl* gene occurs, and β-*geo* is expressed as a reporter of successful gene trapping. Therefore, the *Nipbl*^FIN^ allele is a null allele. *Nanog*^Cre/+^ mice carry a transgene encoding a *Cre* recombinase downstream of a promoter of the *Nanog* gene and initiate recombination in the earliest cells of the embryo (*26*). Consequently, mating *Nanog*^Cre/+^ mice with *Nipbl*^Flox/Flox^ mice results in littermates that are either *Nipbl*^Flox/+^ or *Nipbl*^FIN/+^, entirely. *Nipbl*^Flox/+^ mice express *Nipbl* at WT levels (Fig. 1A) and show no defects, making them essentially WT (*24*). *Nipbl*^FIN/+^ mice express *Nipbl* at levels ~50% lower than WT (fig. S3) and show defects similar to those observed in CdLS, making them essentially *Nipbl*^+/−^ (*24*).

### Timing of mouse pregnancies

To generate female mice that were pregnant on the same day, male mice were singly housed, and female mice were group-housed in groups of five for a minimum of 1 week to synchronize their estrous cycles, thus taking advantage of the Lee-Boot effect (*142*). At the beginning of the night cycle, the bedding from female cages were discarded, and the bedding from at least two male cages were transferred into each of them. At the beginning of the third night cycle after which the females were exposed to male bedding, all females were transferred into male cages, resulting in two females per male, thus taking advantage of the Whitten effect (*143*). At the end of the third night cycle, the females were inspected for vaginal plugs. Those that had vaginal plugs were considered potentially pregnant. To time the dissection of embryos from potentially pregnant females, we considered the end of the 12-hour night cycle after which the vaginal plug was discovered as E0 and dissected the embryos at the following times after E0: E7.5 (7 days +12 hours) for LB stage, E7.75 (7 days +18 hours) for CC stage, E7.41 (7 days +10 hours) for EB stage, E7.58 (7 days +14 hours) for EHF stage embryos, and E18.5 (18 days +12 hours).

### Dissection of mouse embryos

Pregnant female mice were euthanized by CO_2_ inhalation, followed by cervical dislocation. The uterine horns were dissected out of their abdomens with dissection forceps and scissors and placed in a petri dish with 1× diethyl pyrocarbonate (DEPC) phosphate-buffered saline (PBS) on ice. Individual deciduae were separated from one another and transferred into their own petri dishes with 1× DEPC PBS on ice. Embryos were dissected out of each deciduae under a dissection microscope as described in (*144*). The Reichert’s membrane was removed from each embryo. The ectoplacental cone was separated from embryos collected for scRNA-seq and tr4ansferred by forcep into microcentrifuge tubes, where they were kept on ice or stored at 20°C as tissue for PCR genotyping. The exocoelom was separated from embryos collected for scRNA-seq and transferred into a welled plate by wide bore pipette tip with fixative on ice as tissue for genotyping by X-galactosidase (X-gal) stain. Embryos for scRNA-seq were transferred by wide bore pipette tip with 1× DEPC PBS into microcentrifuge tubes and kept on ice until dissociation. Tails were separated from E18.5 embryos collected for Alcian blue–Alizarin red staining. Embryos for Alcian blue–Alizarin red staining were transferred by forcep into 10% neutral buffered formalin in scintillation vials and kept at 4°C for 24 hours.

### Genotyping of mouse embryos and ESCs

#### X-gal stain

Since *Nipbl*^FIN/+^ (*Nipbl*^+/−^) mouse embryos express β-*geo* (β-galactosidase), their tissues will turn blue when they are treated with X-gal, a substrate that releases a blue chromophore when enzymatically acted on by β-galactosidase. The exocoeloms from embryos were transferred into welled plates containing fixative [0.2% glutaraldehyde, 5 mM EGTA, and 2 mM MgCl_2_ in 0.1 M phosphate buffer (pH 7.5)] and kept on ice for a minimum of 15 min. After fixation, the exocoeloms were rinsed with a detergent rinse [0.02% Igepal, 0.01% sodium deoxycholate, and 2 mM MgCl_2_ in 0.1 M phosphate buffer (pH 7.5)] before they were treated with X-gal stain [0.02% Igepal, 0.01% sodium deoxycholate, 5 mM potassium ferricyanide, 5 mM potassium ferrocyanide, and 2 mM MgCl_2_ in 0.1 M phosphate buffer (pH 7.5)]. The exocoeloms were incubated in X-gal stain at 37°C in the dark for a minimum of 1 hour after which they were inspected for coloration under a dissection microscope. Those that turned blue were considered *Nipbl^+/−^* (*Nipbl*^FIN/+^), and those that did not were considered WT.

#### Polymerase chain reaction

Ectoplacental cones were treated with 50 μl proteinase K (60 μg/ml) in PBND [50 mM KCl, 10 mM tris-HCl, 2.5 mM MgCl_2_, gelatin (0.1 mg/ml), 0.45% NP-40, and 0.45% Tween 20], for 1 hour at 55°C to extract DNA for PCR. To deactivate proteinase K so that it does not interfere with DNA amplification, they were then incubated at 95°C for 10 min. To genotype mouse embryos as either *Nipbl*^Flox/+^ (WT) or *Nipbl*^FIN/+^ (*Nipbl*^+/−^) and ESCs as either *Nipbl*^Flrt/+^ (WT) or *Nipbl*^FLEX/+^ (*Nipbl*^+/−^), standard Taq PCR was performed on the extracted DNA using the primers and thermocyling protocol previously described in (*24*): Primer 1, 5′-CTCCGC CTCCTCTTCCTCCATC-3′; primer 2, 5′-CCTCCCCCGTGCCTTCCTTGAC-3′; primer 3, 5′-TTTGAGGGGACGACGACAGTCT-3′. Thermocycling conditions are 1 cycle a 95°C for 30 s; 30 cycles of 95°C for 1 min, 59°C for 30 s, and 68°C for 1 min; and 1 cycle at 68°C for 5 min and hold at 4°C. PCR products were treated with loading dye and electrophoresed in agarose gel stained with SYBER Safe DNA Gel Stain (Invitrogen, S33102) without cleanup and visualized under ultraviolet light. Flox conformation is 782 base pairs (bp), FIN conformation is 518 bp, Flrt conformation is 735 bp, and FLEX conformation is 652 bp.

### Single-cell RNA sequencing

Embryos were transferred by wide bore pipette tip in 20 μl of 1× DEPC PBS into microcentrifuge tubes. A total of 200 μl of 1× TrypLE Express Enzyme with phenol red (Gibco, 12605010) prewarmed to 37°C was added to each embryo. Embryos were triturated 4× with a wide bore pipette tip. Embryos were incubated at 37°C and triturated 4× with a wide bore pipette tip every minute (4 to 8 min) until no tissue aggregates were visible under a dissection microscope. TrypLE Expres Enzyme activity was inactivated with the addition of pre–ice-chilled 200 μl of 0.04% (m/v) nonacetylated bovine serum albumin in Dulbecco’s PBS (Sigma-Aldrich, B6917-100MG; Gibco, 14190144). The resulting cell suspension was underlayed with 200 μl of 1% nonacetylated bovine serum albumin in Hanks’ balanced salt solution (Sigma-Aldrich, 55021C-1000ML) using a gel-loading pipette tip. The suspension was centrifuged in a swing bucket centrifuge at 300 rcf for 5 min at 4°C. Supernatant (600 μl) was removed without disturbing the cell pellet. Hanks’ balanced salt solution (600 μl) was added to the cell pellet, and the cell pellet was resuspended in it using a wide bore pipette tip. The resulting cell suspension was centrifuged in a swing bucket centrifuge at 300 rcf for 5 min at 4°C. Supernatant (600 μl) was removed without disturbing the cell pellet. The cell pellet was resuspending in the remaining 20 μl of supernatant using a wide bore pipette tip.

Single-cell suspensions (20 μl) were submitted to the Genomics High-Throughput Facility (GHTF) at the University of California, Irvine (*145*) for scRNA-seq using 10x Genomics’ Chromium Next GEM Single Cell 3′ Kit v3.1 (10x Genomics, 1000268), Chromium Next GEM Chip G Single Cell Kit (10x Genomics, 1000120), and Chromium Controller. Passage through the Chromium Controller resulted in sample, cell, and transcript-barcoded cDNA, which GHTF-amplified by PCR. GHTF assessed the quality and quantity of the amplified cDNA by electrophoresis using Agilent’s Agilent High Sensitivity DNA Kit (Agilent, 5067-4626) and Bioanalyzer before library construction. Constructed libraries, representing embryonic samples, were multiplexed and sequenced by GHTF on the Illumina HiSeq 4000 to a minimum depth of 20 million read pairs per cell. GHTF demultiplexed Illumina’s raw binary base call (BCL) files and returned FASTQ files as deliverables.

### Read mapping and cell calling

Cell Ranger v3.0 was used to map reads onto the GRCm38/mm10 C57BL/6J *Mus musculus* genome/transcriptome assembly and call cells. Cell Ranger does this using a read mapper called STAR (*146*), which performs splicing-aware mapping of reads to the genome. Reads are considered confidentially mapped to the genome with a mapping quality (MAPQ) of 255. Exonic reads are further mapped to annotated transcripts. A read that is compatible with the exons of an annotated transcript and, aligned to the same strand, is considered mapped to the transcriptome. Cell Ranger called cells using the EmptyDrops method described in (*147*).

### Normalization of library depth

Cell Ranger v3.0 was used to normalize the read depth between libraries of the same stage. Cell Ranger does this by subsampling reads from higher-depth libraries until all libraries of the same stage had an equal number of reads.

### Removal of low-quality cells and doublets

Cells exceeding three median absolute deviations in any one of the following criteria among cells of the same stage were considered either low-quality cells or doublets (*148*) and removed (i) percentage of mitochondrial genes expressed,(ii) number of genes expressed, and/or (iii) number of transcripts detected (figs. S2 and S8).

### Normalization of cell depth

Seurat v3.0 was used to normalize the read depth between cells of the same stage using the SCtransform method, which is described in (*149*). SCtransform does this by modeling the read counts in a regularized negative binomial model to determine the variation due to read depth and then adjusting that variance according to genes of similar abundances. SCtransform was also used to normalize the read depth between cells when cells were subset from the whole embryo into germ layers and clusters.

### Batch effect correction

Seurat v3.0 was used to correct for batch effects among libraries of the same stage and genotype. Seurat does this by identifying a set of shared variable genes among the libraries being considered and, using these genes, identifies pairs of cells between any two libraries whose expression of these genes is similar to other. These pairs of cells act as anchors between libraries for batch effect correction and integration (*30*).

### Clustering of WT cells

Seurat v3.0 was used to cluster WT cells. Seurat does this by first performing principal components analysis on the shared variable genes identified during batch effect correction and integration. Principal components whose explained variances exceeded two median absolute deviations were used to calculate *k*-nearest neighbors and construct a shared nearest-neighbor graph. Clusters were determined by optimization of the modularity function using the Louvain algorithm. The number of clusters was controlled by modulating the resolution function.

The optimal number of clusters was determined by clustering cells at increasing consecutive numbers of clusters and generating a clustering tree (fig. S5) visualizing how cell cluster identities change as the number of clusters consecutively increase. Clusters are stable when a large proportion of cells are derived from a single preceding cluster rather than multiple preceding clusters. We adopted Shannon entropy as a measure of these proportions as a measure of intracluster stability. A low Shannon entropy represents high intracluster stability. We visually inspected the clustering tree to determine which number of clusters maximized the number of clusters while, at the same time, minimizing the total Shannon entropy across all clusters at that number of clusters.

We further performed DGEA between clusters and visualized the expression of the top DEGs in a heatmap. We visually inspected the heatmap to confirm that the number of clusters that was selected for intracluster stability also displayed intercluster differences in gene expression.

### DGEA between clusters

Seurat v3.0 was used to perform DGEA between clusters. For each cluster, Seurat performs the Mann-Whitney *U* test (a nonparametric test) between cells in that cluster and all other cells using normalized read counts. *P* values were corrected for false discovery using the Bonferroni correction method. Genes with *Q* values less than 0.05 were considered statistically significant and differentially expressed.

### Projection of *Nipbl*^+/−^ cells onto WT clusters

Seurat v3.0 was used to project *Nipbl*^+/−^ cells onto WT clusters. Seurat does this by identifying a set of shared variable genes among WT samples and, using these genes, identifies pairs of cells between WT cells and *Nipbl*^+/−^ cells whose expression of these genes are similar to other. These pairs of cells act as anchors between WT cells and *Nipbl*^+/−^ cells for projecting and sorting *Nipbl*^+/−^ cells into WT clusters (*30*).

### Reverse projection

Using the same method that was used to cluster WT cells (see the “Clustering of WT cells” section), cells from LB-stage *Nipbl^+/−^* embryos were first clustered and annotated (fig. S6A). Cells from LB-stage WT embryos were then projected onto the *Nipbl^+/−^* clusters (fig. S6, B to D) (see the “Projection of *Nipbl*^+/−^ cells onto WT clusters” section).

### DGEA between genotypes

Seurat v3.0 was used to perform DGEA between WT and *Nipbl*^+/−^ cells. For each cluster, Seurat performs the Mann-Whitney *U* test between WT and *Nipbl*^+/−^ cells using normalized read counts. *P* values were corrected for false discovery using the Bonferroni correction method. Genes with *Q* values less than 0.05 were considered statistically significant and differentially expressed.

### Pseudo-time

URD was used to calculate the pseudo-time of cells from EB-, LB-, and EHF-stage embryos (*83*). URD does this by constructing a diffusion map of transition probabilities and, starting with an assigned group of root cells, performs a probabilistic breadth-first graph search using the transition probabilities. This moves step-wise outward from the root cells, until the entire graph is visited. Several simulations are run, and then pseudo-time is calculated as the average iteration that visited each cell.

### Construction of lineage trajectories

Velocyto (*150*) was used to count the numbers of spliced and unspliced transcripts per gene, using default parameters. Reads aligning to exonic regions were counted as spliced. Reads aligning to intronic regions were counted as unspliced. Reads aligning to exon-intron boundaries were considered ambiguous and excluded from downstream analyses. scVelo (*84*) was used estimate RNA velocities. Counts were normalized using the *pp.filter_and_normalize()* function, moments of unspliced versus spliced abundances were computed using the *pp.moments()* function, and velocities were computed using the *tl.velocity()* function, all using default parameters. Lineage trajectories were visually inferred from stream plots of computed RNA velocities.

### Calculating fate probabilities

CellRank was used to calculate the fate probabilities of mesoderm cells (*86*). CellRank does this by performing RNA velocity–directed random walks from initial cell states to terminal cell states. Fate probabilities correspond to the fraction of walks in which a cell was a part of that that terminated in a particular terminal cell state. Terminal states were set with the *set_terminal_states()* function, absorption probabilities were computed with the *compute_absorption_probabilities()* function, and driver genes were computed with the *compute_lineage_drivers()* function, all with default parameters.

### Cell cycle phase assignment

Seurat was used to assign cells into G_1_, S, or G_2_-M phase based on the expression of markers of S phase and G_2_-M phase provided by Seurat. Using the *CellCycleScoring()* function, Seurat calculated scores for the expression of S phase and G_2_-M phase markers, while considering the expression of these marker genes to be anticorrelated to one another. When cells express neither, they are considered to be in G_1_ phase.

### Identification of drivers and antidrivers

CellRank was used to identify the drivers and antidrivers of FHF and PM fates. CellRank does this by calculating a correlation coefficient between the fate probabilities of cells toward their lineage fate and the expression of their genes. Those genes with positive correlation coefficients are considered drivers, since their expression increases as absorption probabilities increase, and those with negative correlation coefficients are considered antidrivers, since their expression decreases as absorption probabilities decrease. To reduce the likelihood of false discovery, we considered those genes with correlation coefficients greater than 0.25 as drivers and those less than −0.25 as antidrivers.

### Gene set overrepresentation analysis

clusterProfiler (*151*) was used to perform gene set overrepresentation analysis. It does this by performing a Fisher’s exact test on a contingency table of the genes in a gene set that match or do not match the genes of interest. *P* values were corrected for false discovery using the Bonferroni correction method. Gene sets with *Q* values less than 0.05 were considered statistically significant and over represented by the genes of interest.

### Gene set enrichment analysis

Fast Gene Set Enrichment Analysis (FGSEA) (*152*) was used to perform GSEA.

### Generation of *Nipbl^FLEX/+^* and *Nipbl^Flrt/+^* ESCs

ESCs were grown in Glasgow’s minimum essential medium, 15% heat-inactivated fetal bovine serum (ESC-qualified; HyClone, SH30071.03E), 1× glutamine, 1× penicillin-streptomycin, 5 mM mercaptoethanol, and leukemia inhibitory factor (1000 U/ml; ESGRO Millipore). Following limited dilution single-cell cloning, a clone of EUC313f02 *Nipbl^FLEX/+^* ESCs (European Conditional Mouse Mutagenesis Program) was transfected with pCAG-Cre:GFP (Addgene, #13776) using Lipofectamine 2000 (Invitrogen) to convert the *Nipbl^FLEX^* allele to the *Nipbl^Flrt^* conformation in vitro. Forty-eight hours after transfection, cells were plated at 1 cell per well into several 96-well plates. Clonal colonies were isolated, and the clones were stained for β-galactosidase (*lacZ*) activity using X-gal. For LacZ staining, the ESCs were fixed for 5 min in 2 mM MgCl_2_ and 0.5% glutaraldehyde in 1× PBS, followed by three washes with 1× PBS at room temperature. X-gal staining [5 mM K_3_Fe(CN)_6_, 5 mM K_4_Fe(CN)_6_, 2 mM MgCl_2_, and X-gal (1 mg/ml) in 1× PBS] was performed at 37°C until the blue precipitate was detected. Colonies positive for X-gal staining, *Nipbl^FLEX/+^* ESCs, and negative for X-gal staining, *Nipbl^Flrt/+^*, were verified by PCR genotyping, described above.

### Reverse transcription quantitative polymerase chain reaction

Clones for both *Nipbl^FLEX/+^* and *Nipbl^Flrt/+^* were expanded, nine each, and RNA was extracted using the Monarch Total RNA Miniprep Kit (New England Biolabs). cDNA was made using iSCRIPT reverse transcriptase (Bio-Rad). RT-qPCR for *Nipbl*, *Nanog*, and *Pouf5f1* was performed using iTaq SYBR green (Bio-Rad) as per the manufacturer’s instructions, *Rpl4* was used as the housekeeping gene: *Rpl4* primer 1, 5′-ATCTGGACGGAGAGTGCTTT-3′; *Rpl4* primer 2, 5′-GGTCGGTGTTCATCATCTTG-3′; *Nipbl* primer 1, 5′-AGTCCATATGCCCCACAGAG-3′; *Nipbl* primer 2, 5′-ACCGGCAACAATAGGACTTG-3′; *Nanog* primer 1, 5′-AAATCCCTTCCCTCGCCATC-3′; *Nanog* primer 2, 5′-GCCCTGACTTTAAGCCCAGA-3′; *Pou5f1* primer 1, 5′-CACCCTGGGCGTTCTCTTT-3′; *Pou5f1* primer 2, 5′-GTCTCCGATTTGCATATCTCCTG-3′.

### Identification of genes with Nanog binding sites

In Avsec *et al.* (*119*), ChIP-seq was performed for Nanog in mESCs, and 56,459 peaks were called (Fig. 8F). We considered the called peaks from that study as representing Nanog binding sites. Using the narrowPeak file from that study (which contains the called peaks), the GRCm38/mm10 C57BL/6J *M. musculus* genome annotation, and ChIPseeker v3, we identified which genes had a peak of ±250 nt of their TSS. We identified a total of 3646 genes with a Nanog binding site at their TSS (Fig. 8F).

### Alcian blue–Alizarin red staining

E18.5 embryos were fixed in 10% neutral buffered formalin in scintillation vials at 4°C overnight. Embryos were washed with H_2_O twice over 2 days at room temperature. Embryos were immersed in 95% ethanol (EtOH) for 1 week. The skin was removed from embryos with forceps under dissection microscope. Embryos were stained with 0.02% Alcian blue (10 mg of Alcian blue + 80 ml of EtOH + 20 ml of glacial acetic acid) over 3 days. Embryos were washed with 70% EtOH twice in 1 day, 40% EtOH overnight, 15% EtOH in 1 day, and H_2_O overnight. Embryos were washed with 1% KOH twice over 3 days until they became translucent. Embryos were stained with 0.015% Alizarin red (15 mg of Alizarin red + 100 ml of 1% KOH) over 3 days. Embryos were washed three times with 1% KOH in 1 day. Fat pads and internal organs were removed from embryos with forceps under dissection microscope. Embryos were washed with 20% glycerol + 1% KOH overnight, 50% glycerol + 1% KOH overnight, and 80% glycerol + 1% KOH overnight. Embryos were stored in 100% glycerol at room temperature.

## References

[1] Centers for Disease Control and Prevention, Update on overall prevalence of major birth defects–Atlanta, Georgia, 1978–2005. MMWR Morb. Mortal. Wkly. Rep. 57, 1–5 (2008). 18185492

[2] C. T. Mai, J. L. Isenburg, M. A. Canfield, R. E. Meyer, A. Correa, C. J. Alverson, P. J. Lupo, T. Riehle-Colarusso, S. J. Cho, D. Aggarwal, R. S. Kirby, National Birth Defects Prevention Network, National population-based estimates for major birth defects, 2010-2014. Birth Defects Res 111, 1420–1435 (2019). 31580536 10.1002/bdr2.1589 PMC7203968

[3] R. Sun, M. Liu, L. Lu, Y. Zheng, P. Zhang, Congenital heart disease: Causes, diagnosis, symptoms, and treatments. Cell Biochem. Biophys. 72, 857–860 (2015). 25638345 10.1007/s12013-015-0551-6

[4] D. M. Ely, A. K. Driscoll, Infant mortality in the United States, 2020: Data from the period linked birth/infant death file. Natl. Vital Stat. Rep. 71, 1–18 (2022). 36190428

[5] L. Mannini, F. Cucco, V. Quarantotti, I. D. Krantz, A. Musio, Mutation spectrum and genotype-phenotype correlation in Cornelia de Lange syndrome. Hum. Mutat. 34, 1589–1596 (2013). 24038889 10.1002/humu.22430 PMC3880228

[6] A. D. Kline, I. D. Krantz, A. Sommer, M. Kliewer, L. G. Jackson, D. R. FitzPatrick, A. V. Levin, A. Selicorni, Cornelia de Lange syndrome: Clinical review, diagnostic and scoring systems, and anticipatory guidance. Am. J. Med. Genet. A 143A, 1287–1296 (2007). 17508425 10.1002/ajmg.a.31757

[7] Z. A. Bhuiyan, M. Klein, P. Hammond, A. van Haeringen, M. M. Mannens, I. van Berckelaer-Onnes, R. C. Hennekam, Genotype-phenotype correlations of 39 patients with Cornelia de Lange syndrome: The Dutch experience. J. Med. Genet. 43, 568–575 (2006). 16236812 10.1136/jmg.2005.038240 PMC2564552

[8] L. A. Gillis, J. McCallum, M. Kaur, C. DeScipio, D. Yaeger, A. Mariani, A. D. Kline, H. H. Li, M. Devoto, L. G. Jackson, I. D. Krantz, NIPBL mutational analysis in 120 individuals with Cornelia de Lange syndrome and evaluation of genotype-phenotype correlations. Am. J. Hum. Genet. 75, 610–623 (2004). 15318302 10.1086/424698 PMC1182048

[9] G. Borck, M. Zarhrate, C. Cluzeau, E. Bal, J. P. Bonnefont, A. Munnich, V. Cormier-Daire, L. Colleaux, Father-to-daughter transmission of Cornelia de Lange syndrome caused by a mutation in the 5′ untranslated region of the NIPBL Gene. Hum. Mutat. 27, 731–735 (2006). 16799922 10.1002/humu.20380

[10] R. Ciosk, M. Shirayama, A. Shevchenko, T. Tanaka, A. Toth, A. Shevchenko, K. Nasmyth, Cohesin’s binding to chromosomes depends on a separate complex consisting of Scc2 and Scc4 proteins. Mol. Cell 5, 243–254 (2000). 10882066 10.1016/s1097-2765(00)80420-7

[11] K. Nasmyth, C. H. Haering, Cohesin: Its roles and mechanisms. Annu. Rev. Genet. 43, 525–558 (2009). 19886810 10.1146/annurev-genet-102108-134233

[12] A. Musio, A. Selicorni, M. L. Focarelli, C. Gervasini, D. Milani, S. Russo, P. Vezzoni, L. Larizza, X-linked Cornelia de Lange syndrome owing to SMC1L1 mutations. Nat. Genet. 38, 528–530 (2006). 16604071 10.1038/ng1779

[13] M. A. Deardorff, M. Kaur, D. Yaeger, A. Rampuria, S. Korolev, J. Pie, C. Gil-Rodríguez, M. Arnedo, B. Loeys, A. D. Kline, M. Wilson, K. Lillquist, V. Siu, F. J. Ramos, A. Musio, L. S. Jackson, D. Dorsett, I. D. Krantz, Mutations in cohesin complex members SMC3 and SMC1A cause a mild variant of cornelia de Lange syndrome with predominant mental retardation. Am. J. Hum. Genet. 80, 485–494 (2007). 17273969 10.1086/511888 PMC1821101

[14] L. Mannini, S. Menga, A. Tonelli, S. Zanotti, M. T. Bassi, C. Magnani, A. Musio, SMC1A codon 496 mutations affect the cellular response to genotoxic treatments. Am. J. Med. Genet. A 158A, 224–228 (2012). 22140011 10.1002/ajmg.a.34384

[15] M. A. Deardorff, M. Bando, R. Nakato, E. Watrin, T. Itoh, M. Minamino, K. Saitoh, M. Komata, Y. Katou, D. Clark, K. E. Cole, E. de Baere, C. Decroos, N. di Donato, S. Ernst, L. J. Francey, Y. Gyftodimou, K. Hirashima, M. Hullings, Y. Ishikawa, C. Jaulin, M. Kaur, T. Kiyono, P. M. Lombardi, L. Magnaghi-Jaulin, G. R. Mortier, N. Nozaki, M. B. Petersen, H. Seimiya, V. M. Siu, Y. Suzuki, K. Takagaki, J. J. Wilde, P. J. Willems, C. Prigent, G. Gillessen-Kaesbach, D. W. Christianson, F. J. Kaiser, L. G. Jackson, T. Hirota, I. D. Krantz, K. Shirahige, HDAC8 mutations in Cornelia de Lange syndrome affect the cohesin acetylation cycle. Nature 489, 313–317 (2012). 22885700 10.1038/nature11316 PMC3443318

[16] M. A. Deardorff, J. J. Wilde, M. Albrecht, E. Dickinson, S. Tennstedt, D. Braunholz, M. Mönnich, Y. Yan, W. Xu, M. C. Gil-Rodríguez, D. Clark, H. Hakonarson, S. Halbach, L. D. Michelis, A. Rampuria, E. Rossier, S. Spranger, L. van Maldergem, S. A. Lynch, G. Gillessen-Kaesbach, H. J. Lüdecke, R. G. Ramsay, M. J. McKay, I. D. Krantz, H. Xu, J. A. Horsfield, F. J. Kaiser, RAD21 mutations cause a human cohesinopathy. Am. J. Hum. Genet. 90, 1014–1027 (2012). 22633399 10.1016/j.ajhg.2012.04.019 PMC3370273

[17] M. Kaur, C. DeScipio, J. McCallum, D. Yaeger, M. Devoto, L. G. Jackson, N. B. Spinner, I. D. Krantz, Precocious sister chromatid separation (PSCS) in Cornelia de Lange syndrome. Am. J. Med. Genet. A 138, 27–31 (2005). 16100726 10.1002/ajmg.a.30919 PMC2766539

[18] S. Kawauchi, A. L. Calof, R. Santos, M. E. Lopez-Burks, C. M. Young, M. P. Hoang, A. Chua, T. Lao, M. S. Lechner, J. A. Daniel, A. Nussenzweig, L. Kitzes, K. Yokomori, B. Hallgrimsson, A. D. Lander, Multiple organ system defects and transcriptional dysregulation in the *Nipbl^+/−^* mouse, a model of Cornelia de Lange syndrome. PLOS Genet. 5, e1000650 (2009). 19763162 10.1371/journal.pgen.1000650 PMC2730539

[19] D. Dorsett, I. D. Krantz, On the molecular etiology of Cornelia de Lange syndrome. Ann. N. Y. Acad. Sci. 1151, 22–37 (2009). 19154515 10.1111/j.1749-6632.2008.03450.x PMC2733214

[20] R. Chien, W. Zeng, S. Kawauchi, M. A. Bender, R. Santos, H. C. Gregson, J. A. Schmiesing, D. A. Newkirk, X. Kong, A. R. Ball Jr., A. L. Calof, A. D. Lander, M. T. Groudine, K. Yokomori, Cohesin mediates chromatin interactions that regulate mammalian β-globin expression. J. Biol. Chem. 286, 17870–17878 (2011). 21454523 10.1074/jbc.M110.207365 PMC3093862

[21] Y. Guo, K. Monahan, H. Wu, J. Gertz, K. E. Varley, W. Li, R. M. Myers, T. Maniatis, Q. Wu, CTCF/cohesin-mediated DNA looping is required for protocadherin α promoter choice. Proc. Natl. Acad. Sci. U.S.A. 109, 21081–21086 (2012). 23204437 10.1073/pnas.1219280110 PMC3529044

[22] A. Muto, A. L. Calof, A. D. Lander, T. F. Schilling, Multifactorial origins of heart and gut defects in nipbl-deficient zebrafish, a model of Cornelia de Lange syndrome. PLoS Biol. 9, e1001181 (2011). 22039349 10.1371/journal.pbio.1001181 PMC3201921

[23] B. Yuan, D. Pehlivan, E. Karaca, N. Patel, W. L. Charng, T. Gambin, C. Gonzaga-Jauregui, V. R. Sutton, G. Yesil, S. T. Bozdogan, T. Tos, A. Koparir, E. Koparir, C. R. Beck, S. Gu, H. Aslan, O. O. Yuregir, K. al Rubeaan, D. Alnaqeb, M. J. Alshammari, Y. Bayram, M. M. Atik, H. Aydin, B. B. Geckinli, M. Seven, H. Ulucan, E. Fenercioglu, M. Ozen, S. Jhangiani, D. M. Muzny, E. Boerwinkle, B. Tuysuz, F. S. Alkuraya, R. A. Gibbs, J. R. Lupski, Global transcriptional disturbances underlie Cornelia de Lange syndrome and related phenotypes. J. Clin. Invest. 125, 636–651 (2015). 25574841 10.1172/JCI77435 PMC4319410

[24] R. Santos, S. Kawauchi, R. E. Jacobs, M. E. Lopez-Burks, H. Choi, J. Wikenheiser, B. Hallgrimsson, H. A. Jamniczky, S. E. Fraser, A. D. Lander, A. L. Calof, Conditional creation and rescue of Nipbl-deficiency in mice reveals multiple determinants of risk for congenital heart defects. PLoS Biol. 14, e2000197 (2016). 27606604 10.1371/journal.pbio.2000197 PMC5016002

[25] M. A. Mole, A. Weberling, M. Zernicka-Goetz, Comparative analysis of human and mouse development: From zygote to pre-gastrulation. Curr. Top. Dev. Biol. 136, 113–138 (2020). 31959285 10.1016/bs.ctdb.2019.10.002

[26] A. N. Economides, D. Frendewey, P. Yang, M. G. Dominguez, A. T. Dore, I. B. Lobov, T. Persaud, J. Rojas, J. McClain, P. Lengyel, G. Droguett, R. Chernomorsky, S. Stevens, W. Auerbach, T. M. Dechiara, W. Pouyemirou, Cruz JM Jr, K. Feeley, I. A. Mellis, J. Yasenchack, S. J. Hatsell, L. Xie, E. Latres, L. Huang, Y. Zhang, E. Pefanis, D. Skokos, R. A. Deckelbaum, S. D. Croll, S. Davis, D. M. Valenzuela, N. W. Gale, A. J. Murphy, G. D. Yancopoulos, Conditionals by inversion provide a universal method for the generation of conditional alleles. Proc. Natl. Acad. Sci. U.S.A. 110, E3179–E3188 (2013). 23918385 10.1073/pnas.1217812110 PMC3752204

[27] K. M. Downs, T. Davies, Staging of gastrulating mouse embryos by morphological landmarks in the dissecting microscope. Development 118, 1255–1266 (1993). 8269852 10.1242/dev.118.4.1255

[28] G. X. Zheng, J. M. Terry, P. Belgrader, P. Ryvkin, Z. W. Bent, R. Wilson, S. B. Ziraldo, T. D. Wheeler, G. P. McDermott, J. Zhu, M. T. Gregory, J. Shuga, L. Montesclaros, J. G. Underwood, D. A. Masquelier, S. Y. Nishimura, M. Schnall-Levin, P. W. Wyatt, C. M. Hindson, R. Bharadwaj, A. Wong, K. D. Ness, L. W. Beppu, H. J. Deeg, C. McFarland, K. R. Loeb, W. J. Valente, N. G. Ericson, E. A. Stevens, J. P. Radich, T. S. Mikkelsen, B. J. Hindson, J. H. Bielas, Massively parallel digital transcriptional profiling of single cells. Nat. Commun. 8, 14049 (2017). 28091601 10.1038/ncomms14049 PMC5241818

[29] M. Ginsburg, M. H. Snow, A. McLaren, Primordial germ cells in the mouse embryo during gastrulation. Development 110, 521–528 (1990). 2133553 10.1242/dev.110.2.521

[30] T. Stuart, A. Butler, P. Hoffman, C. Hafemeister, E. Papalexi, W. M. Mauck III, Y. Hao, M. Stoeckius, P. Smibert, R. Satija, Comprehensive integration of single-cell data. Cell 177, 1888–1902.e21 (2019). 31178118 10.1016/j.cell.2019.05.031 PMC6687398

[31] A. Okuda, A. Fukushima, M. Nishimoto, A. Orimo, T. Yamagishi, Y. Nabeshima, M. Kuro-o, Y. I. Nabeshima, K. Boon, M. Keaveney, H. G. Stunnenberg, M. Muramatsu, UTF1, a novel transcriptional coactivator expressed in pluripotent embryonic stem cells and extra-embryonic cells. EMBO J. 17, 2019–2032 (1998). 9524124 10.1093/emboj/17.7.2019 PMC1170547

[32] S. Zhang, W. Cui, Sox2, a key factor in the regulation of pluripotency and neural differentiation. World J. Stem. Cells 6, 305–311 (2014). 25126380 10.4252/wjsc.v6.i3.305 PMC4131272

[33] Q. Zhu, L. Song, G. Peng, N. Sun, J. Chen, T. Zhang, N. Sheng, W. Tang, C. Qian, Y. Qiao, K. Tang, J. D. J. Han, J. Li, N. Jing, The transcription factor Pou3f1 promotes neural fate commitment via activation of neural lineage genes and inhibition of external signaling pathways. eLife 3, (2014). 10.7554/eLife.02224 PMC4095939 24929964

[34] V. Wilson, L. Manson, W. C. Skarnes, R. S. Beddington, The T gene is necessary for normal mesodermal morphogenetic cell movements during gastrulation. Development 121, 877–886 (1995). 7720590 10.1242/dev.121.3.877

[35] R. M. Barnes, B. A. Firulli, S. J. Conway, J. W. Vincentz, A. B. Firulli, Analysis of the Hand1 cell lineage reveals novel contributions to cardiovascular, neural crest, extra-embryonic, and lateral mesoderm derivatives. Dev. Dyn. 239, 3086–3097 (2010). 20882677 10.1002/dvdy.22428 PMC2965316

[36] B. Saykali, N. Mathiah, W. Nahaboo, M. L. Racu, L. Hammou, M. Defrance, I. Migeotte, Distinct mesoderm migration phenotypes in extra-embryonic and embryonic regions of the early mouse embryo. eLife 8, e42434 (2019). 30950395 10.7554/eLife.42434 PMC6450669

[37] H. Bildsoe, X. Fan, E. E. Wilkie, A. Ashoti, V. J. Jones, M. Power, J. Qin, J. Wang, P. P. L. Tam, D. A. F. Loebel, Transcriptional targets of TWIST1 in the cranial mesoderm regulate cell-matrix interactions and mesenchyme maintenance. Dev. Biol. 418, 189–203 (2016). 27546376 10.1016/j.ydbio.2016.08.016

[38] B. Leussink, A. Brouwer, M. el Khattabi, R. E. Poelmann, A. C. Gittenberger-de Groot, F. Meijlink, Expression patterns of the paired-related homeobox genes MHox/Prx1 and S8/Prx2 suggest roles in development of the heart and the forebrain. Mech. Dev. 52, 51–64 (1995). 7577675 10.1016/0925-4773(95)00389-i

[39] G. S. Kwon, A. K. Hadjantonakis, Transthyretin mouse transgenes direct RFP expression or Cre-mediated recombination throughout the visceral endoderm. Genesis 47, 447–455 (2009). 19415627 10.1002/dvg.20522 PMC2878311

[40] L. F. Chu, N. Leng, J. Zhang, Z. Hou, D. Mamott, D. T. Vereide, J. Choi, C. Kendziorski, R. Stewart, J. A. Thomson, Single-cell RNA-seq reveals novel regulators of human embryonic stem cell differentiation to definitive endoderm. Genome Biol. 17, 173 (2016). 27534536 10.1186/s13059-016-1033-x PMC4989499

[41] I. Burtscher, H. Lickert, Foxa2 regulates polarity and epithelialization in the endoderm germ layer of the mouse embryo. Development 136, 1029–1038 (2009). 19234065 10.1242/dev.028415

[42] W. S. Chen, K. Manova, D. C. Weinstein, S. A. Duncan, A. S. Plump, V. R. Prezioso, R. F. Bachvarova, J. E. Darnell, Disruption of the HNF-4 gene, expressed in visceral endoderm, leads to cell death in embryonic ectoderm and impaired gastrulation of mouse embryos. Genes Dev. 8, 2466–2477 (1994). 7958910 10.1101/gad.8.20.2466

[43] S. F. Khan, V. Damerell, R. Omar, M. du Toit, M. Khan, H. M. Maranyane, M. Mlaza, J. Bleloch, C. Bellis, B. D. B. Sahm, J. Peres, K. N. ArulJothi, S. Prince, The roles and regulation of TBX3 in development and disease. Gene 726, 144223 (2020). 31669645 10.1016/j.gene.2019.144223 PMC7108957

[44] Y. Li, P. Lemaire, R. R. Behringer, Esx1, a novel X chromosome-linked homeobox gene expressed in mouse extraembryonic tissues and male germ cells. Dev. Biol. 188, 85–95 (1997). 9245514 10.1006/dbio.1997.8640

[45] A. Simeone, M. Gulisano, D. Acampora, A. Stornaiuolo, M. Rambaldi, E. Boncinelli, Two vertebrate homeobox genes related to the Drosophila empty spiracles gene are expressed in the embryonic cerebral cortex. EMBO J. 11, 2541–2550 (1992). 1352754 10.1002/j.1460-2075.1992.tb05319.x PMC556729

[46] B. I. Meyer, P. Gruss, MouseCdx-1expression during gastrulation. Development 117, 191–203 (1993). 7900985 10.1242/dev.117.1.191

[47] A. Rodrigo Albors, P. A. Halley, K. G. Storey, Lineage tracing axial progenitors using Nkx1-2CreERT2 mice defines their trunk and tail contributions. Development 145, (2018). 10.1242/dev.164319 PMC6198475 30201686

[48] Y. Qu, B. Zhou, W. Yang, B. Han, Y. Yu-Rice, B. Gao, J. Johnson, C. N. Svendsen, M. R. Freeman, A. E. Giuliano, D. Sareen, X. Cui, Transcriptome and proteome characterization of surface ectoderm cells differentiated from human iPSCs. Sci. Rep. 6, 32007 (2016). 27550649 10.1038/srep32007 PMC4994084

[49] M. Donnison, A. Beaton, H. W. Davey, R. Broadhurst, P. L'Huillier, P. L. Pfeffer, Loss of the extraembryonic ectoderm in Elf5 mutants leads to defects in embryonic patterning. Development 132, 2299–2308 (2005). 15829518 10.1242/dev.01819

[50] N. Nishioka, S. Yamamoto, H. Kiyonari, H. Sato, A. Sawada, M. Ota, K. Nakao, H. Sasaki, Tead4 is required for specification of trophectoderm in pre-implantation mouse embryos. Mech. Dev. 125, 270–283 (2008). 18083014 10.1016/j.mod.2007.11.002

[51] B. Pijuan-Sala, J. A. Griffiths, C. Guibentif, T. W. Hiscock, W. Jawaid, F. J. Calero-Nieto, C. Mulas, X. Ibarra-Soria, R. C. V. Tyser, D. L. L. Ho, W. Reik, S. Srinivas, B. D. Simons, J. Nichols, J. C. Marioni, B. Göttgens, A single-cell molecular map of mouse gastrulation and early organogenesis. Nature 566, 490–495 (2019). 30787436 10.1038/s41586-019-0933-9 PMC6522369

[52] G. Gestri, M. Carl, I. Appolloni, S. W. Wilson, G. Barsacchi, M. Andreazzoli, Six3 functions in anterior neural plate specification by promoting cell proliferation and inhibiting Bmp4 expression. Development 132, 2401–2413 (2005). 15843413 10.1242/dev.01814 PMC2789257

[53] C. L. Andoniadou, M. Signore, E. Sajedi, C. Gaston-Massuet, D. Kelberman, A. J. Burns, N. Itasaki, M. Dattani, J. P. Martinez-Barbera, Lack of the murine homeobox gene Hesx1 leads to a posterior transformation of the anterior forebrain. Development 134, 1499–1508 (2007). 17360769 10.1242/dev.02829 PMC2233881

[54] M. T. Alves dos Santos, M. P. Smidt, En1 and Wnt signaling in midbrain dopaminergic neuronal development. Neural Dev. 6, 23 (2011). 21569278 10.1186/1749-8104-6-23 PMC3104484

[55] D. H. Rowitch, A. P. McMahon, Pax-2 expression in the murine neural plate precedes and encompasses the expression domains of Wnt-1 and En-1. Mech. Dev. 52, 3–8 (1995). 7577673 10.1016/0925-4773(95)00380-j

[56] A. Gavalas, C. Ruhrberg, J. Livet, C. E. Henderson, R. Krumlauf, Neuronal defects in the hindbrain of Hoxa1, Hoxb1 and Hoxb2 mutants reflect regulatory interactions among these Hox genes. Development 130, 5663–5679 (2003). 14522873 10.1242/dev.00802

[57] C. S. Hong, J. P. Saint-Jeannet, The b-HLH transcription factor Hes3 participates in neural plate border formation by interfering with Wnt/β-catenin signaling. Dev. Biol. 442, 162–172 (2018). 30016640 10.1016/j.ydbio.2018.07.011 PMC6138557

[58] G. Cho, Y. Lim, I. T. Cho, J. C. Simonet, J. A. Golden, Arx together with FoxA2, regulates Shh floor plate expression. Dev. Biol. 393, 137–148 (2014). 24968361 10.1016/j.ydbio.2014.06.012 PMC4681535

[59] A. Holz, H. Kollmus, J. Ryge, V. Niederkofler, J. Dias, J. Ericson, E. T. Stoeckli, O. Kiehn, H. H. Arnold, The transcription factors Nkx2.2 and Nkx2.9 play a novel role in floor plate development and commissural axon guidance. Development 137, 4249–4260 (2010). 21068056 10.1242/dev.053819 PMC2990212

[60] C. Papanayotou, A. Mey, A. M. Birot, Y. Saka, S. Boast, J. C. Smith, J. Samarut, C. D. Stern, A mechanism regulating the onset of Sox2 expression in the embryonic neural plate. PLOS Biol. 6, e2 (2008). 10.1371/journal.pbio.0060002 PMC2174969 18184035

[61] J. D. Lee, N. F. Silva-Gagliardi, U. Tepass, C. J. McGlade, K. V. Anderson, The FERM protein Epb4.1l5 is required for organization of the neural plate and for the epithelial-mesenchymal transition at the primitive streak of the mouse embryo. Development 134, 2007–2016 (2007). 17507402 10.1242/dev.000885

[62] C. Milet, F. Maczkowiak, D. D. Roche, A. H. Monsoro-Burq, Pax3 and Zic1 drive induction and differentiation of multipotent, migratory, and functional neural crest in Xenopus embryos. Proc. Natl. Acad. Sci. U.S.A. 110, 5528–5533 (2013). 23509273 10.1073/pnas.1219124110 PMC3619367

[63] L. Teng, N. A. Mundell, A. Y. Frist, Q. Wang, P. A. Labosky, Requirement for Foxd3 in the maintenance of neural crest progenitors. Development 135, 1615–1624 (2008). 18367558 10.1242/dev.012179 PMC2562748

[64] S. S. Chan, X. Shi, A. Toyama, R. W. Arpke, A. Dandapat, M. Iacovino, J. Kang, G. Le, H. R. Hagen, D. J. Garry, M. Kyba, Mesp1 patterns mesoderm into cardiac, hematopoietic, or skeletal myogenic progenitors in a context-dependent manner. Cell Stem Cell 12, 587–601 (2013). 23642367 10.1016/j.stem.2013.03.004 PMC3646300

[65] M. R. Jongbloed, R. Vicente-Steijn, Y. L. Douglas, L. J. Wisse, K. Mori, Y. Yokota, M. M. Bartelings, M. J. Schalij, E. A. Mahtab, R. E. Poelmann, A. C. G.-D. Groot, Expression of Id2 in the second heart field and cardiac defects in Id2 knock-out mice. Dev. Dyn. 240, 2561–2577 (2011). 22012595 10.1002/dvdy.22762

[66] E. Dodou, M. P. Verzi, J. P. Anderson, S. M. Xu, B. L. Black, Mef2c is a direct transcriptional target of ISL1 and GATA factors in the anterior heart field during mouse embryonic development. Development 131, 3931–3942 (2004). 15253934 10.1242/dev.01256

[67] T. Sadahiro, M. Isomi, N. Muraoka, H. Kojima, S. Haginiwa, S. Kurotsu, F. Tamura, H. Tani, S. Tohyama, J. Fujita, H. Miyoshi, Y. Kawamura, N. Goshima, Y. W. Iwasaki, K. Murano, K. Saito, M. Oda, P. Andersen, C. Kwon, H. Uosaki, H. Nishizono, K. Fukuda, M. Ieda, Tbx6 induces nascent mesoderm from pluripotent stem cells and temporally controls cardiac versus somite lineage diversification. Cell Stem Cell 23, 382–395.e5 (2018). 30100166 10.1016/j.stem.2018.07.001 PMC6190602

[68] F. Cecconi, G. Proetzel, G. Alvarez-Bolado, D. Jay, P. Gruss, Expression of Meis2, a Knotted-related murine homeobox gene, indicates a role in the differentiation of the forebrain and the somitic mesoderm. Dev. Dyn. 210, 184–190 (1997). 9337138 10.1002/(SICI)1097-0177(199710)210:2<184::AID-AJA10>3.0.CO;2-E

[69] B. Wilm, R. G. James, T. M. Schultheiss, B. L. Hogan, The forkhead genes, Foxc1 and Foxc2, regulate paraxial versus intermediate mesoderm cell fate. Dev. Biol. 271, 176–189 (2004). 15196959 10.1016/j.ydbio.2004.03.034

[70] O. R. Davies, C. Y. Lin, A. Radzisheuskaya, X. Zhou, J. Taube, G. Blin, A. Waterhouse, A. J. H. Smith, S. Lowell, Tcf15 primes pluripotent cells for differentiation. Cell Rep. 3, 472–484 (2013). 23395635 10.1016/j.celrep.2013.01.017 PMC3607254

[71] J. M. Khor, C. A. Ettensohn, Transcription factors of the Alx family: Evolutionarily conserved regulators of deuterostome skeletogenesis. Front. Genet. 11, 569314 (2020). 33329706 10.3389/fgene.2020.569314 PMC7719703

[72] M. Gering, Y. Yamada, T. H. Rabbitts, R. K. Patient, Lmo2 and Scl/Tal1 convert non-axial mesoderm into haemangioblasts which differentiate into endothelial cells in the absence of Gata1. Development 130, 6187–6199 (2003). 14602685 10.1242/dev.00875

[73] N. Koyano-Nakagawa, D. J. Garry, Etv2 as an essential regulator of mesodermal lineage development. Cardiovasc. Res. 113, 1294–1306 (2017). 28859300 10.1093/cvr/cvx133 PMC5852632

[74] K. Briegel, P. Bartunek, G. Stengl, K. C. Lim, H. Beug, J. D. Engel, M. Zenke, Regulation and function of transcription factor GATA-1 during red blood cell differentiation. Development 122, 3839–3850 (1996). 9012505 10.1242/dev.122.12.3839

[75] A. Beckers, L. Alten, C. Viebahn, P. Andre, A. Gossler, The mouse homeobox gene Noto regulates node morphogenesis, notochordal ciliogenesis, and left right patterning. Proc. Natl. Acad. Sci. U.S.A. 104, 15765–15770 (2007). 17884984 10.1073/pnas.0704344104 PMC2000419

[76] J. L. Stubbs, I. Oishi, J. C. Izpisua Belmonte, C. Kintner, The forkhead protein Foxj1 specifies node-like cilia in Xenopus and zebrafish embryos. Nat. Genet. 40, 1454–1460 (2008). 19011629 10.1038/ng.267 PMC4648715

[77] J. Schemmer, M. J. Araúzo-Bravo, N. Haas, S. Schäfer, S. N. Weber, A. Becker, D. Eckert, A. Zimmer, D. Nettersheim, H. Schorle, Transcription factor TFAP2C regulates major programs required for murine fetal germ cell maintenance and haploinsufficiency predisposes to teratomas in male mice. PLOS ONE 8, e71113 (2013). 23967156 10.1371/journal.pone.0071113 PMC3742748

[78] J. Sun, M. C. Ting, M. Ishii, R. Maxson, Msx1 and Msx2 function together in the regulation of primordial germ cell migration in the mouse. Dev. Biol. 417, 11–24 (2016). 27435625 10.1016/j.ydbio.2016.07.013 PMC5407493

[79] E. L. Maska, P. Cserjesi, L. L. Hua, M. E. Garstka, H. M. Brody, Y. Morikawa, A Tlx2-Cre mouse line uncovers essential roles for hand1 in extraembryonic and lateral mesoderm. Genesis 48, 479–484 (2010). 20506548 10.1002/dvg.20644 PMC2955361

[80] C. A. Desjardins, F. J. Naya, The Function of the MEF2 Family of Transcription Factors in Cardiac Development, Cardiogenomics, and Direct Reprogramming. J. Cardiovasc. Dev. Dis. 3, 26 (2016). 27630998 10.3390/jcdd3030026 PMC5019174

[81] V. Serpooshan, Y. H. Liu, J. W. Buikema, F. X. Galdos, O. Chirikian, S. Paige, S. Venkatraman, A. Kumar, D. R. Rawnsley, X. Huang, D. A. Pijnappels, S. M. Wu, Nkx2.5^+^ cardiomyoblasts contribute to cardiomyogenesis in the neonatal heart. Sci. Rep. 7, 12590 (2017). 28974782 10.1038/s41598-017-12869-4 PMC5626718

[82] T. Jukkola, R. Trokovic, P. Maj, A. Lamberg, B. Mankoo, V. Pachnis, H. Savilahti, J. Partanen, Meox1Cre: A mouse line expressing Cre recombinase in somitic mesoderm. Genesis 43, 148–153 (2005). 16267823 10.1002/gene.20163

[83] J. A. Farrell, Y. Wang, S. J. Riesenfeld, K. Shekhar, A. Regev, A. F. Schier, Single-cell reconstruction of developmental trajectories during zebrafish embryogenesis. Science 360, eaar3131 (2018). 29700225 10.1126/science.aar3131 PMC6247916

[84] V. Bergen, M. Lange, S. Peidli, F. A. Wolf, F. J. Theis, Generalizing RNA velocity to transient cell states through dynamical modeling. Nat. Biotechnol. 38, 1408–1414 (2020). 32747759 10.1038/s41587-020-0591-3

[85] Y. Saga, S. Miyagawa-Tomita, A. Takagi, S. Kitajima, J. I. Miyazaki, T. Inoue, MesP1 is expressed in the heart precursor cells and required for the formation of a single heart tube. Development 126, 3437–3447 (1999). 10393122 10.1242/dev.126.15.3437

[86] M. Lange, V. Bergen, M. Klein, M. Setty, B. Reuter, M. Bakhti, H. Lickert, M. Ansari, J. Schniering, H. B. Schiller, D. Pe’er, F. J. Theis, CellRank for directed single-cell fate mapping. Nat. Methods 19, 159–170 (2022). 35027767 10.1038/s41592-021-01346-6 PMC8828480

[87] A. Liberzon, C. Birger, H. Thorvaldsdóttir, M. Ghandi, J. P. Mesirov, P. Tamayo, The Molecular Signatures Database (MSigDB) hallmark gene set collection. Cell Syst 1, 417–425 (2015). 26771021 10.1016/j.cels.2015.12.004 PMC4707969

[88] M. Milacic, D. Beavers, P. Conley, C. Gong, M. Gillespie, J. Griss, R. Haw, B. Jassal, L. Matthews, B. May, R. Petryszak, E. Ragueneau, K. Rothfels, C. Sevilla, V. Shamovsky, R. Stephan, K. Tiwari, T. Varusai, J. Weiser, A. Wright, G. Wu, L. Stein, H. Hermjakob, P. D’Eustachio, The Reactome Pathway Knowledgebase 2024. Nucleic Acids Res. 52, D672–D678 (2024). 37941124 10.1093/nar/gkad1025 PMC10767911

[89] S. M. Langemeijer, N. Mariani, R. Knops, C. Gilissen, R. Woestenenk, T. de Witte, G. Huls, B. A. van der Reijden, J. H. Jansen, Apoptosis-related gene expression profiling in hematopoietic cell fractions of MDS patients. PLOS ONE 11, e0165582 (2016). 27902785 10.1371/journal.pone.0165582 PMC5130187

[90] D. Venet, J. E. Dumont, V. Detours, Most random gene expression signatures are significantly associated with breast cancer outcome. PLoS Comput. Biol. 7, e1002240 (2011). 22028643 10.1371/journal.pcbi.1002240 PMC3197658

[91] S. Nestorowa, F. K. Hamey, B. Pijuan Sala, E. Diamanti, M. Shepherd, E. Laurenti, N. K. Wilson, D. G. Kent, B. Göttgens, A single-cell resolution map of mouse hematopoietic stem and progenitor cell differentiation. Blood 128, e20–e31 (2016). 27365425 10.1182/blood-2016-05-716480 PMC5305050

[92] Y. Chen, D. Xiao, L. Zhang, C. L. Cai, B. Y. Li, Y. Liu, The role of Tbx20 in cardiovascular development and function. Front. Cell Dev. Biol. 9, 638542 (2021). 33585493 10.3389/fcell.2021.638542 PMC7876368

[93] D. W. Sorensen, J. H. van Berlo, The role of TGF-β signaling in cardiomyocyte proliferation. Curr. Heart Fail. Rep. 17, 225–233 (2020). 32686010 10.1007/s11897-020-00470-2 PMC7486245

[94] C. Guibentif, J. A. Griffiths, I. Imaz-Rosshandler, S. Ghazanfar, J. Nichols, V. Wilson, B. Göttgens, J. C. Marioni, Diverse routes toward early somites in the mouse embryo. Dev. Cell 56, 141–153.e6 (2021). 33308481 10.1016/j.devcel.2020.11.013 PMC7808755

[95] D. T. Farmer, P. Patel, R. Choi, C. Y. Liu, J. G. Crump, A comprehensive series of Irx cluster mutants reveals diverse roles in facial cartilage development. Development 148, dev197244 (2021). 34338288 10.1242/dev.197244 PMC8406535

[96] H. Preiss, A. C. Kögler, D. Mörsdorf, D. Čapek, G. H. Soh, K. W. Rogers, H. Morales-Navarrete, M. Almuedo-Castillo, P. Müller, Regulation of Nodal signaling propagation by receptor interactions and positive feedback. eLife 11, e66397 (2022). 36149406 10.7554/eLife.66397 PMC9612913

[97] S. Nowotschin, I. Costello, A. Piliszek, G. S. Kwon, C. A. Mao, W. H. Klein, E. J. Robertson, A. K. Hadjantonakis, The T-box transcription factor Eomesodermin is essential for AVE induction in the mouse embryo. Genes Dev. 27, 997–1002 (2013). 23651855 10.1101/gad.215152.113 PMC3656330

[98] C. Meno, J. Takeuchi, R. Sakuma, K. Koshiba-Takeuchi, S. Ohishi, Y. Saijoh, J. I. Miyazaki, P. ten Dijke, T. Ogura, H. Hamada, Diffusion of nodal signaling activity in the absence of the feedback inhibitor Lefty2. Dev. Cell 1, 127–138 (2001). 11703930 10.1016/s1534-5807(01)00006-5

[99] I. Costello, S. Nowotschin, X. Sun, A. W. Mould, A. K. Hadjantonakis, E. K. Bikoff, E. J. Robertson, Lhx1 functions together with Otx2, Foxa2, and Ldb1 to govern anterior mesendoderm, node, and midline development. Genes Dev. 29, 2108–2122 (2015). 26494787 10.1101/gad.268979.115 PMC4617976

[100] M. Siatecka, J. J. Bieker, The multifunctional role of EKLF/KLF1 during erythropoiesis. Blood 118, 2044–2054 (2011). 21613252 10.1182/blood-2011-03-331371 PMC3292426

[101] W. K. Shen, S. Y. Chen, Z. Q. Gan, Y. Z. Zhang, T. Yue, M. M. Chen, Y. Xue, H. Hu, A. Y. Guo, AnimalTFDB 4.0: A comprehensive animal transcription factor database updated with variation and expression annotations. Nucleic Acids Res. 51, D39–D45 (2023). 36268869 10.1093/nar/gkac907 PMC9825474

[102] S. J. Bray, Notch signalling: A simple pathway becomes complex. Nat. Rev. Mol. Cell Biol. 7, 678–689 (2006). 16921404 10.1038/nrm2009

[103] R. Macdonald, K. A. Barth, Q. Xu, N. Holder, I. Mikkola, S. W. Wilson, Midline signalling is required for Pax gene regulation and patterning of the eyes. Development 121, 3267–3278 (1995). 7588061 10.1242/dev.121.10.3267

[104] J. Park, J. J. R. Zhang, A. Moro, M. Kushida, M. Wegner, P. C. W. Kim, Regulation of Sox9 by Sonic Hedgehog (Shh) is essential for patterning and formation of tracheal cartilage. Dev. Dyn. 239, 514–526 (2010). 20034104 10.1002/dvdy.22192

[105] P. H. Su, R. L. Huang, H. C. Lai, L. Y. Chen, Y. C. Weng, C. C. Wang, C. C. Wu, NKX6-1 mediates cancer stem-like properties and regulates sonic hedgehog signaling in leiomyosarcoma. J. Biomed. Sci. 28, 32 (2021). 33906647 10.1186/s12929-021-00726-6 PMC8077933

[106] C. Cai, J. Thorne, L. Grabel, Hedgehog serves as a mitogen and survival factor during embryonic stem cell neurogenesis. Stem Cells 26, 1097–1108 (2008). 18308949 10.1634/stemcells.2007-0684

[107] S. W. Choy, C. W. Cheng, S. T. Lee, V. W. T. Li, M. N. Y. Hui, C. C. Hui, D. Liu, S. H. Cheng, A cascade of irx1a and irx2a controls shh expression during retinogenesis. Dev. Dyn. 239, 3204–3214 (2010). 21046643 10.1002/dvdy.22462

[108] D. Szklarczyk, R. Kirsch, M. Koutrouli, K. Nastou, F. Mehryary, R. Hachilif, A. L. Gable, T. Fang, N. T. Doncheva, S. Pyysalo, P. Bork, L. J. Jensen, C. von Mering, The STRING database in 2023: Protein-protein association networks and functional enrichment analyses for any sequenced genome of interest. Nucleic Acids Res. 51, D638–D646 (2023). 36370105 10.1093/nar/gkac1000 PMC9825434

[109] K. Mitsui, Y. Tokuzawa, H. Itoh, K. Segawa, M. Murakami, K. Takahashi, M. Maruyama, M. Maeda, S. Yamanaka, The homeoprotein Nanog is required for maintenance of pluripotency in mouse epiblast and ES cells. Cell 113, 631–642 (2003). 12787504 10.1016/s0092-8674(03)00393-3

[110] A. H. Hart, L. Hartley, M. Ibrahim, L. Robb, Identification, cloning and expression analysis of the pluripotency promoting Nanog genes in mouse and human. Dev. Dyn. 230, 187–198 (2004). 15108323 10.1002/dvdy.20034

[111] M. Zhang, H. G. Leitch, W. W. C. Tang, N. Festuccia, E. Hall-Ponsele, J. Nichols, M. A. Surani, A. Smith, I. Chambers, Esrrb complementation rescues development of Nanog-null germ cells. Cell Rep. 22, 332–339 (2018). 29320730 10.1016/j.celrep.2017.12.060 PMC5775501

[112] G. Shi, Y. Jin, Role of Oct4 in maintaining and regaining stem cell pluripotency. Stem Cell Res. Ther. 1, 39 (2010). 21156086 10.1186/scrt39 PMC3025441

[113] M. Tiana, E. Lopez-Jimenez, J. S. de Aja, A. Barral, J. Victorino, C. Badia-Careaga, I. Rollan, R. Rouco, E. Santos, H. Sanchez-Iranzo, R. D. Acemel, C. Torroja, J. Adan, E. Andres-Leon, J. L. Gomez-Skarmeta, G. Giovinazzo, F. Sanchez-Cabo, M. Manzanares, Pluripotency factors regulate the onset of Hox cluster activation in the early embryo. Sci. Adv. 8, eabo3583 (2022). 35857513 10.1126/sciadv.abo3583 PMC9286507

[114] M. Mossahebi-Mohammadi, M. Quan, J. S. Zhang, X. Li, FGF signaling pathway: A key regulator of stem cell pluripotency. Front. Cell Dev. Biol. 8, 79 (2020). 32133359 10.3389/fcell.2020.00079 PMC7040165

[115] L. L. Chang, D. S. Kessler, Foxd3 is an essential Nodal-dependent regulator of zebrafish dorsal mesoderm development. Dev. Biol. 342, 39–50 (2010). 20346935 10.1016/j.ydbio.2010.03.017 PMC2866760

[116] M. M. Shen, Nodal signaling: Developmental roles and regulation. Development 134, 1023–1034 (2007). 17287255 10.1242/dev.000166

[117] C. Porcher, H. Chagraoui, M. S. Kristiansen, SCL/TAL1: A multifaceted regulator from blood development to disease. Blood 129, 2051–2060 (2017). 28179281 10.1182/blood-2016-12-754051

[118] V. S. Stanulovic, P. Cauchy, S. A. Assi, M. Hoogenkamp, LMO2 is required for TAL1 DNA binding activity and initiation of definitive haematopoiesis at the haemangioblast stage. Nucleic Acids Res. 45, 9874–9888 (2017). 28973433 10.1093/nar/gkx573 PMC5622341

[119] Z. Avsec, M. Weilert, A. Shrikumar, S. Krueger, A. Alexandari, K. Dalal, R. Fropf, C. McAnany, J. Gagneur, A. Kundaje, J. Zeitlinger, Base-resolution models of transcription-factor binding reveal soft motif syntax. Nat. Genet. 53, 354–366 (2021). 33603233 10.1038/s41588-021-00782-6 PMC8812996

[120] K. A. Hubert, D. M. Wellik, Hox genes in development and beyond. Development 150, dev192476 (2023). 36645372 10.1242/dev.192476 PMC10216783

[121] O. Medina-Martinez, A. Bradley, R. Ramirez-Solis, A large targeted deletion of Hoxb1-Hoxb9 produces a series of single-segment anterior homeotic transformations. Dev. Biol. 222, 71–83 (2000). 10885747 10.1006/dbio.2000.9683

[122] M. I. Boyle, C. Jespersgaard, K. Brondum-Nielsen, A. M. Bisgaard, Z. Tumer, Cornelia de Lange syndrome. Clin. Genet. 88, 1–12 (2015). 25209348 10.1111/cge.12499

[123] S. Kawauchi, R. Santos, A. Muto, M. E. Lopez-Burks, T. F. Schilling, A. D. Lander, A. L. Calof, Using mouse and zebrafish models to understand the etiology of developmental defects in Cornelia de Lange syndrome. Am. J. Med. Genet. C Semin. Med. Genet. 172, 138–145 (2016). 27120001 10.1002/ajmg.c.31484 PMC4924516

[124] M. P. Lambert, L. G. Jackson, D. Clark, M. Kaur, I. D. Krantz, M. A. Deardorff, The incidence of thrombocytopenia in children with Cornelia de Lange syndrome. Am. J. Med. Genet. A 155A, 33–37 (2011). 21204208 10.1002/ajmg.a.33631 PMC3058755

[125] A. Ayerza Casas, B. Puisac Uriol, M. E. Teresa Rodrigo, M. Hernández Marcos, F. J. Ramos Fuentes, J. Pie Juste, Cornelia de Lange syndrome: Congenital heart disease in 149 patients. Med. Clin. (Barc) 149, 300–302 (2017). 28629661 10.1016/j.medcli.2017.03.051

[126] M. E. Lopez-Burks, R. Santos, S. Kawauchi, A. L. Calof, A. D. Lander, Genetic enhancement of limb defects in a mouse model of Cornelia de Lange syndrome. Am. J. Med. Genet. C Semin. Med. Genet. 172, 146–154 (2016). 27120109 10.1002/ajmg.c.31491 PMC4921392

[127] D. Mehta, S. A. S. Vergano, M. Deardorff, S. Aggarwal, A. Barot, D. M. Johnson, N. F. Miller, S. E. Noon, M. Kaur, L. Jackson, I. D. Krantz, Characterization of limb differences in children with Cornelia de Lange syndrome. Am. J. Med. Genet. C Semin. Med. Genet. 172, 155–162 (2016). 27120260 10.1002/ajmg.c.31498

[128] C. Cortes, A. Francou, C. De Bono, R. G. Kelly, Epithelial properties of the second heart field. Circ. Res. 122, 142–154 (2018). 29301846 10.1161/CIRCRESAHA.117.310838

[129] A. von Gise, W. T. Pu, Endocardial and epicardial epithelial to mesenchymal transitions in heart development and disease. Circ. Res. 110, 1628–1645 (2012). 22679138 10.1161/CIRCRESAHA.111.259960 PMC3427736

[130] R. M. George, G. Maldonado-Velez, A. B. Firulli, The heart of the neural crest: Cardiac neural crest cells in development and regeneration. Development 147, (2020). 10.1242/dev.188706 PMC7578353 33060096

[131] A. Muto, S. Ikeda, M. E. Lopez-Burks, Y. Kikuchi, A. L. Calof, A. D. Lander, T. F. Schilling, Nipbl and mediator cooperatively regulate gene expression to control limb development. PLOS Genet. 10, e1004671 (2014). 25255084 10.1371/journal.pgen.1004671 PMC4177752

[132] F. A. Lee, F. M. Kenny, Skeletal changes in the Cornelia de Lange syndrome. Am. J. Roentgenol. Radium Ther. Nucl. Med. 100, 27–39 (1967). 10.2214/ajr.100.1.27 6023899

[133] J. Brennan, D. P. Norris, E. J. Robertson, Nodal activity in the node governs left-right asymmetry. Genes Dev. 16, 2339–2344 (2002). 12231623 10.1101/gad.1016202 PMC187443

[134] B. Mohapatra, B. Casey, H. Li, T. Ho-Dawson, L. Smith, S. D. Fernbach, L. Molinari, S. R. Niesh, J. L. Jefferies, W. J. Craigen, J. A. Towbin, J. W. Belmont, S. M. Ware, Identification and functional characterization of NODAL rare variants in heterotaxy and isolated cardiovascular malformations. Hum. Mol. Genet. 18, 861–871 (2009). 19064609 10.1093/hmg/ddn411 PMC2722226

[135] K. C. Chatfield, S. A. Schrier, J. Li, D. Clark, M. Kaur, A. D. Kline, M. A. Deardorff, L. S. Jackson, E. Goldmuntz, I. D. Krantz, Congenital heart disease in Cornelia de Lange syndrome: Phenotype and genotype analysis. Am. J. Med. Genet. A 158A, 2499–2505 (2012). 22965847 10.1002/ajmg.a.35582 PMC3551981

[136] T. H. Kim, Z. K. Abdullaev, A. D. Smith, K. A. Ching, D. I. Loukinov, R. D. Green, M. Q. Zhang, V. V. Lobanenkov, B. Ren, Analysis of the vertebrate insulator protein CTCF-binding sites in the human genome. Cell 128, 1231–1245 (2007). 17382889 10.1016/j.cell.2006.12.048 PMC2572726

[137] W. Schwarzer, N. Abdennur, A. Goloborodko, A. Pekowska, G. Fudenberg, Y. Loe-Mie, N. A. Fonseca, W. Huber, C. H. Haering, L. Mirny, F. Spitz, Two independent modes of chromatin organization revealed by cohesin removal. Nature 551, 51–56 (2017). 29094699 10.1038/nature24281 PMC5687303

[138] A. S. Hansen, C. Cattoglio, X. Darzacq, R. Tjian, Recent evidence that TADs and chromatin loops are dynamic structures. Nucleus 9, 20–32 (2018). 29077530 10.1080/19491034.2017.1389365 PMC5990973

[139] M. Panarotto, I. F. Davidson, G. Litos, A. Schleiffer, J. M. Peters, Cornelia de Lange syndrome mutations in NIPBL can impair cohesin-mediated DNA loop extrusion. Proc. Natl. Acad. Sci. U.S.A. 119, e2201029119 (2022). 35476527 10.1073/pnas.2201029119 PMC9170158

[140] D. Hnisz, B. J. Abraham, T. I. Lee, A. Lau, V. Saint-André, A. A. Sigova, H. A. Hoke, R. A. Young, Super-enhancers in the control of cell identity and disease. Cell 155, 934–947 (2013). 24119843 10.1016/j.cell.2013.09.053 PMC3841062

[141] D. A. Newkirk, Y. Y. Chen, R. Chien, W. Zeng, J. Biesinger, E. Flowers, S. Kawauchi, R. Santos, A. L. Calof, A. D. Lander, X. Xie, K. Yokomori, The effect of Nipped-B-like (Nipbl) haploinsufficiency on genome-wide cohesin binding and target gene expression: Modeling Cornelia de Lange syndrome. Clin. Epigenetics 9, 89 (2017). 28855971 10.1186/s13148-017-0391-x PMC5574093

[142] S. Van Der Lee, L. M. Boot, Spontaneous pseudopregnancy in mice. Acta Physiol. Pharmacol. Neerl. 4, 442–444 (1955). 13301816

[143] W. K. Whitten, Modification of the oestrous cycle of the mouse by external stimuli associated with the male. J. Endocrinol. 13, 399–404 (1956). 13345955 10.1677/joe.0.0130399

[144] K. Shea, N. Geijsen, Dissection of 6.5 dpc mouse embryos. J. Vis. Exp. 160, (2007). 10.3791/160 PMC2532933 18830421

[145] S. Das, Q. B. Chen, J. D. Saucier, B. Drescher, Y. Zong, S. Morgan, J. Forstall, A. Meriwether, R. Toranzo, S. M. Leal, The *Drosophila* T-box transcription factor Midline functions within the Notch-Delta signaling pathway to specify sensory organ precursor cell fates and regulates cell survival within the eye imaginal disc. Mech. Dev. 130, 577–601 (2013). 23962751 10.1016/j.mod.2013.08.001 PMC4500660

[146] A. Dobin, C. A. Davis, F. Schlesinger, J. Drenkow, C. Zaleski, S. Jha, P. Batut, M. Chaisson, T. R. Gingeras, STAR: Ultrafast universal RNA-seq aligner. Bioinformatics 29, 15–21 (2013). 23104886 10.1093/bioinformatics/bts635 PMC3530905

[147] A. T. L. Lun, S. Riesenfeld, T. Andrews, T. P. Dao, T. Gomes, participants in the 1st Human Cell Atlas Jamboree, J. C. Marioni, EmptyDrops: Distinguishing cells from empty droplets in droplet-based single-cell RNA sequencing data. Genome Biol. 20, 63 (2019). 30902100 10.1186/s13059-019-1662-y PMC6431044

[148] T. Ilicic, J. K. Kim, A. A. Kolodziejczyk, F. O. Bagger, D. J. McCarthy, J. C. Marioni, S. A. Teichmann, Classification of low quality cells from single-cell RNA-seq data. Genome Biol. 17, 29 (2016). 26887813 10.1186/s13059-016-0888-1 PMC4758103

[149] C. Hafemeister, R. Satija, Normalization and variance stabilization of single-cell RNA-seq data using regularized negative binomial regression. Genome Biol. 20, 296 (2019). 31870423 10.1186/s13059-019-1874-1 PMC6927181

[150] G. La Manno, R. Soldatov, A. Zeisel, E. Braun, H. Hochgerner, V. Petukhov, K. Lidschreiber, M. E. Kastriti, P. Lönnerberg, A. Furlan, J. Fan, L. E. Borm, Z. Liu, D. van Bruggen, J. Guo, X. He, R. Barker, E. Sundström, G. Castelo-Branco, P. Cramer, I. Adameyko, S. Linnarsson, P. V. Kharchenko, RNA velocity of single cells. Nature 560, 494–498 (2018). 30089906 10.1038/s41586-018-0414-6 PMC6130801

[151] T. Wu, E. Hu, S. Xu, M. Chen, P. Guo, Z. Dai, T. Feng, L. Zhou, W. Tang, L. Zhan, X. Fu, S. Liu, X. Bo, G. Yu, clusterProfiler 4.0: A universal enrichment tool for interpreting omics data. Innovation (Camb) 2, 100141 (2021). 34557778 10.1016/j.xinn.2021.100141 PMC8454663

[152] G. Korotkevich, Fast gene set enrichment analysis. bioRxiv, 060012 (2021).

