# Supplementary material for: Gastrulation-stage gene expression in Nipbl+/− mouse embryos foreshadows the development of syndromic birth defects
Source: Sci Adv. 2024 Mar 20;10(12):eadl4239. doi: 10.1126/sciadv.adl4239 (PMC10954218; doi:10.1126/sciadv.adl4239)
Supplement: Supplementary file 1 — Figs. S1 to S15 Tables S1 to S11 Legends for data S1 to S45 [file sciadv.adl4239_sm.pdf]

Science Advances
AAAS

Supplementary Materials for

Gastrulation-stage gene expression in *Nipbl*$^{+/-}$ mouse embryos foreshadows the development of syndromic birth defects

Stephenson Chea *et al.*

Corresponding author: Stephenson Chea, schea2@uci.edu; Anne L. Calof, alcalof@uci.edu

Sci. Adv. **10**, eadl4239 (2024)
DOI: 10.1126/sciadv.adl4239

The PDF file includes:

Figs. S1 to S15
Tables S1 to S11
Legends for data S1 to S45

Other Supplementary Material for this manuscript includes the following:

Data S1 to S45

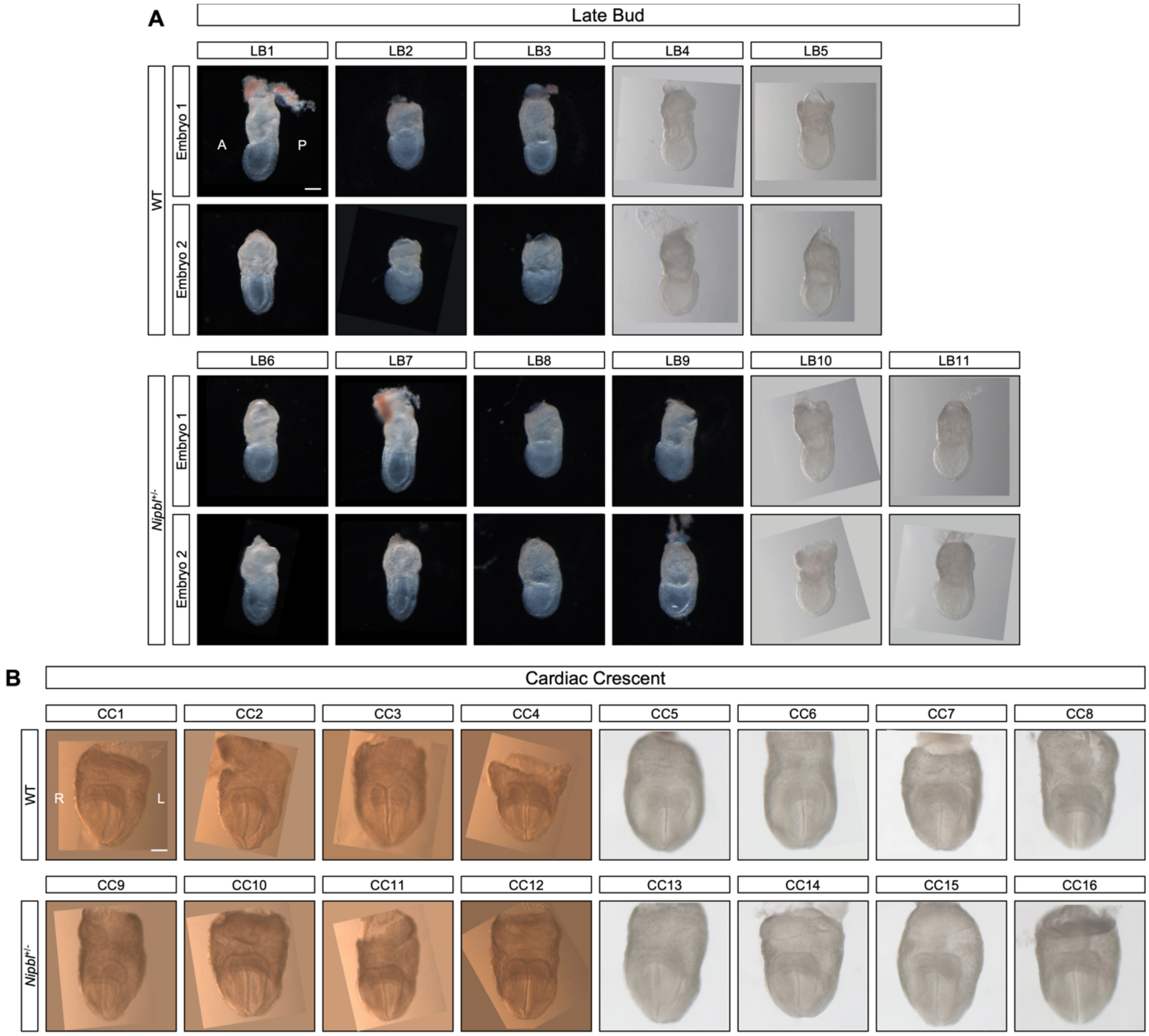

Figure S1. Images of LB- and CC-stage WT and *Nipbl*$^{+/-}$ mouse embryos used for scRNAseq.

Lateral view of (**A**) LB-stage and (**B**) anterior view of CC-stage WT and *Nipbl*$^{+/-}$ mouse embryos. A = anterior, P = posterior, L = left, R = right, and scale bar = 100 um.

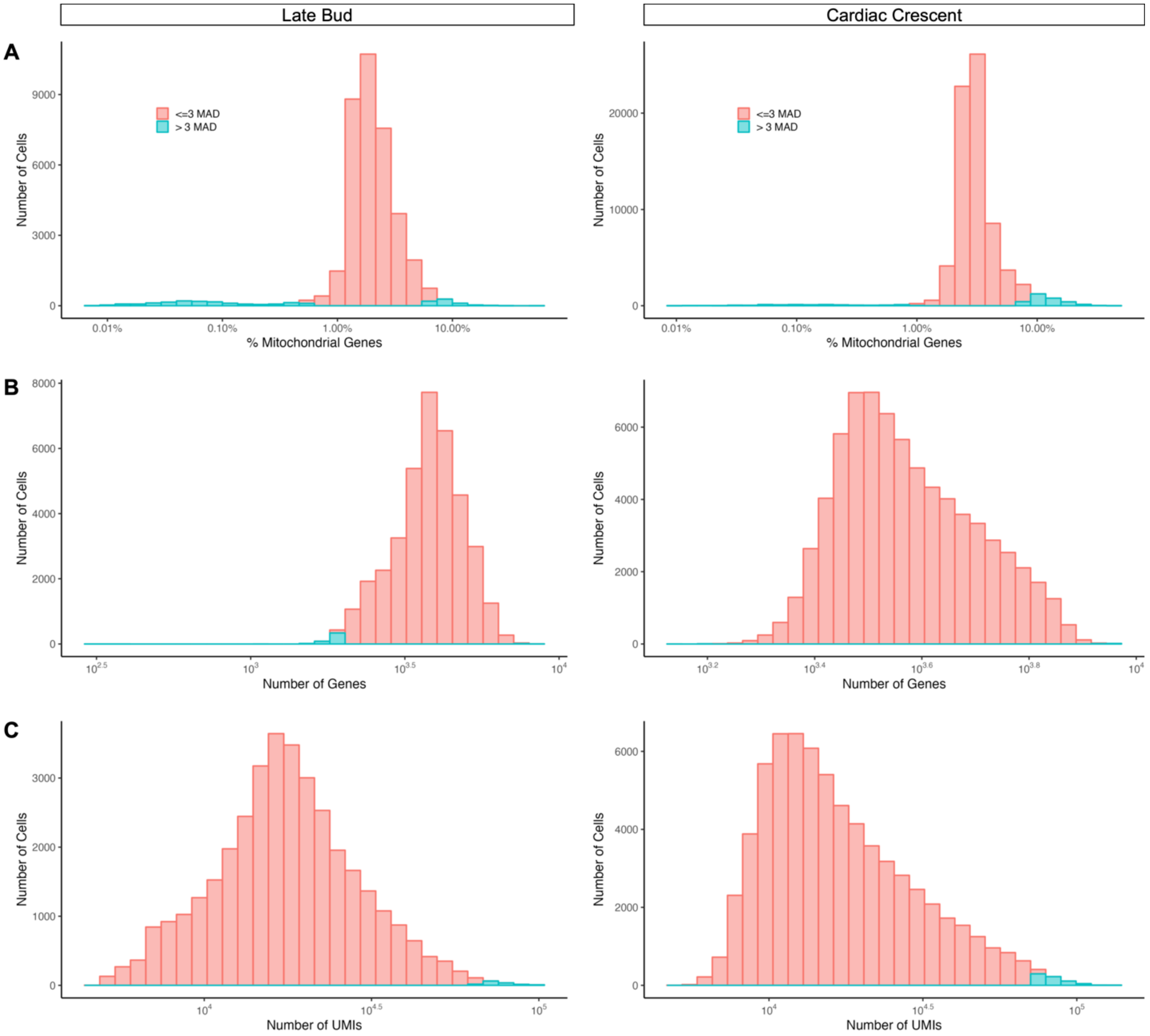

Figure S2. Metrics used to identify low-quality cells and doublets among LB- and CC-stage embryos.

At each stage, we considered cells that exceeded three median absolute deviations from percent mitochondrial genes expressed, number of genes expressed, and number of transcripts detected per cell, low-quality cells and/or doublets and removed them. Histograms of (**A**) percent mitochondrial genes expressed, (**B**) number of genes expressed, and (**C**) number of transcripts detected per cell at LB- and CC-stage colored by whether cells were less than or equal to 3 median absolute deviations (red) or greater than 3 median absolute deviations (blue).

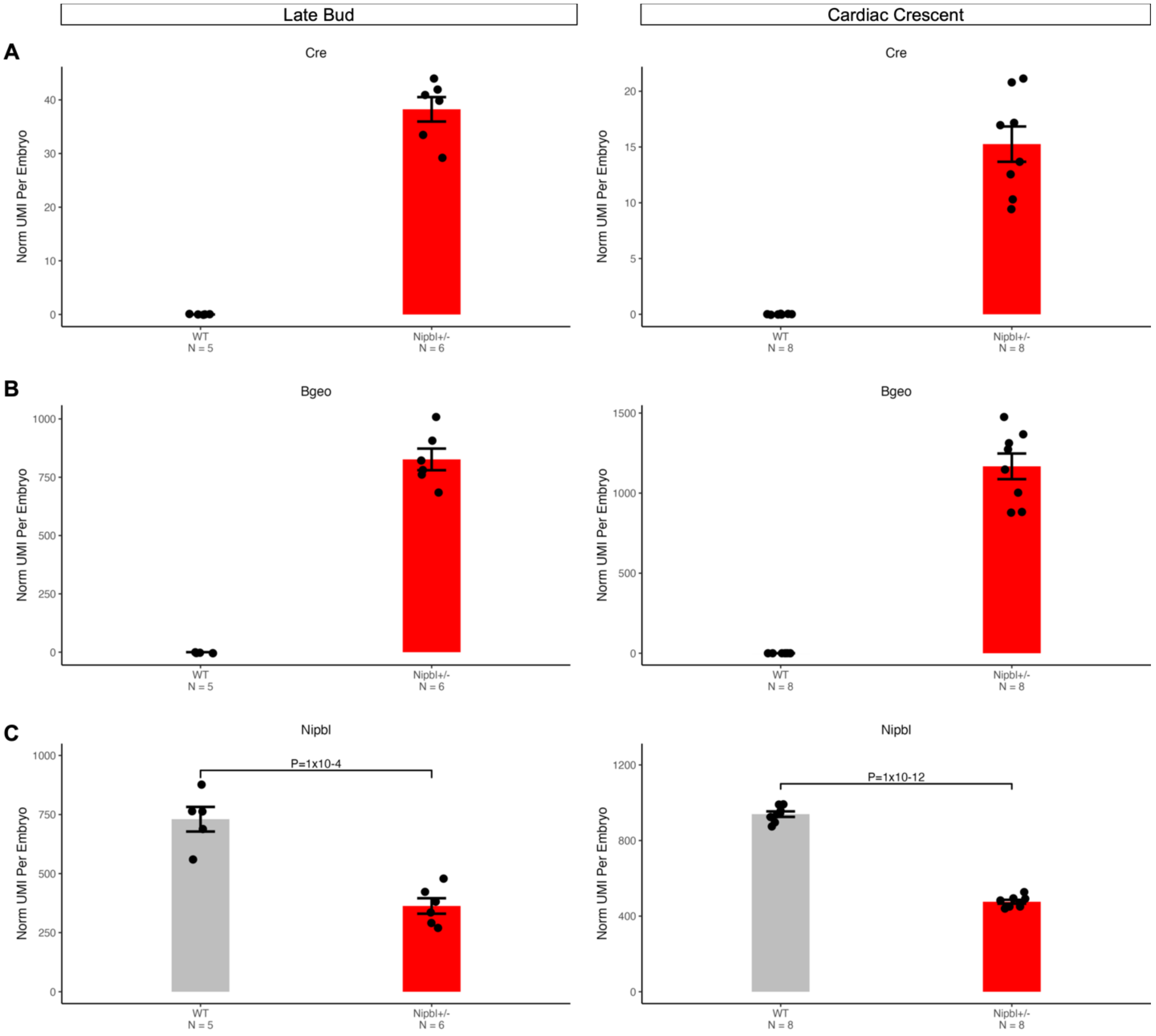

Figure S3. Expression of Cre, Bgeo, and Nipbl in LB- and CC-stage WT and *Nipbl*$^{+/-}$ embryos.

Nipbl$^{+/-}$ embryos at LB- and CC-stage express Cre and Bgeo, while WT embryos do not. *Nipbl*$^{+/-}$ embryos also express *Nipbl* at levels lower than that of WT embryos. Normalized transcripts of (A) *Cre*, (B) *Bgeo*, and (C) *Nipbl* in LB- and CC-stage embryos. Each dot indicates an individual sample. Error bars show standard error of the mean. *P*-value from T-Test.

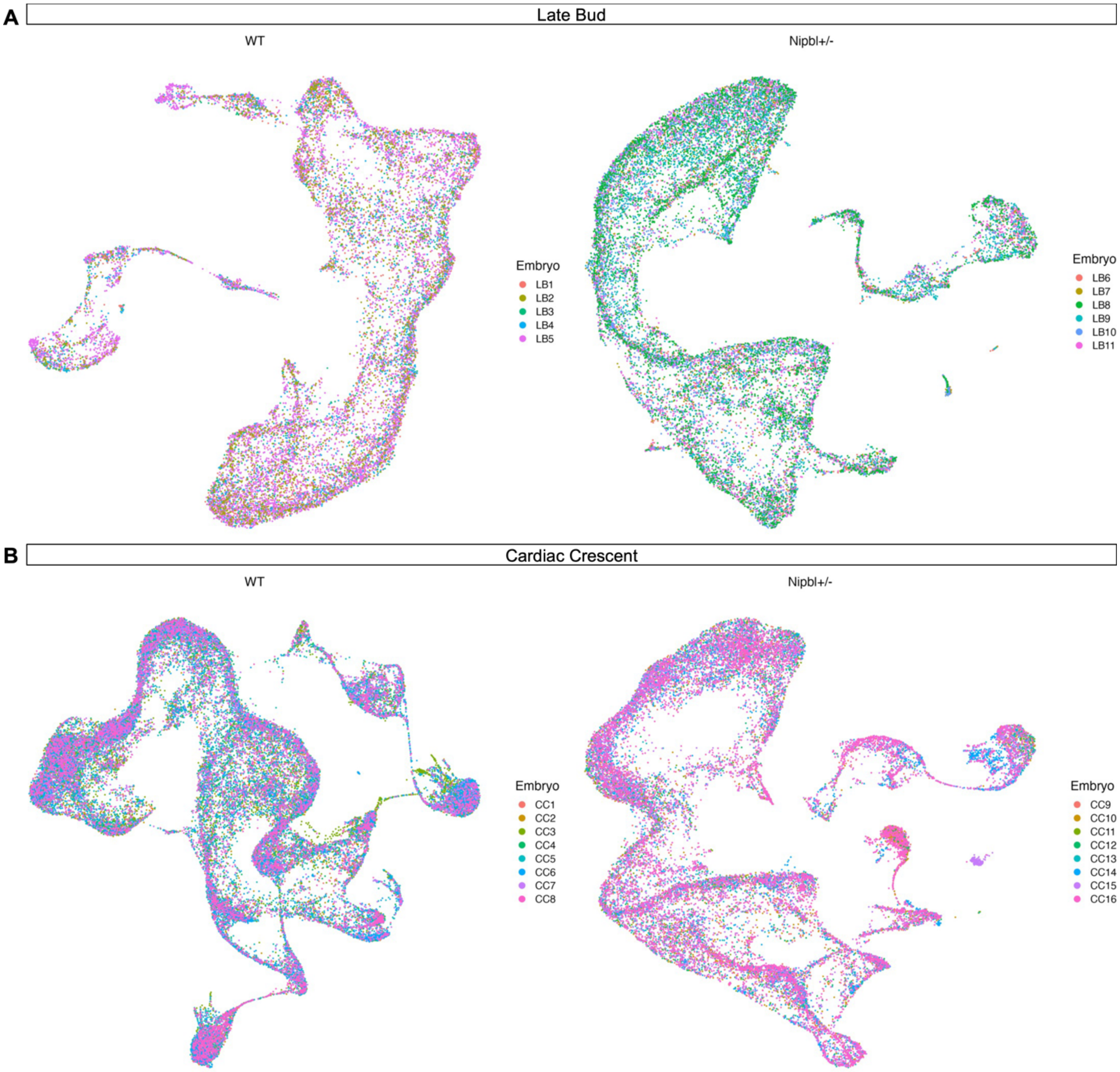

Fig. S4. Batch effect correction and integration of cells of the same stage (LB- or CC-stage) and genotype (WT or *Nipbl*$^{+/-}$).

We corrected batch effects among embryos by integrating cells from the same stage and genotype together. UMAP of WT and *Nipbl*$^{+/-}$ cells colored by embryo at (A) LB- and (B) CC-stage.

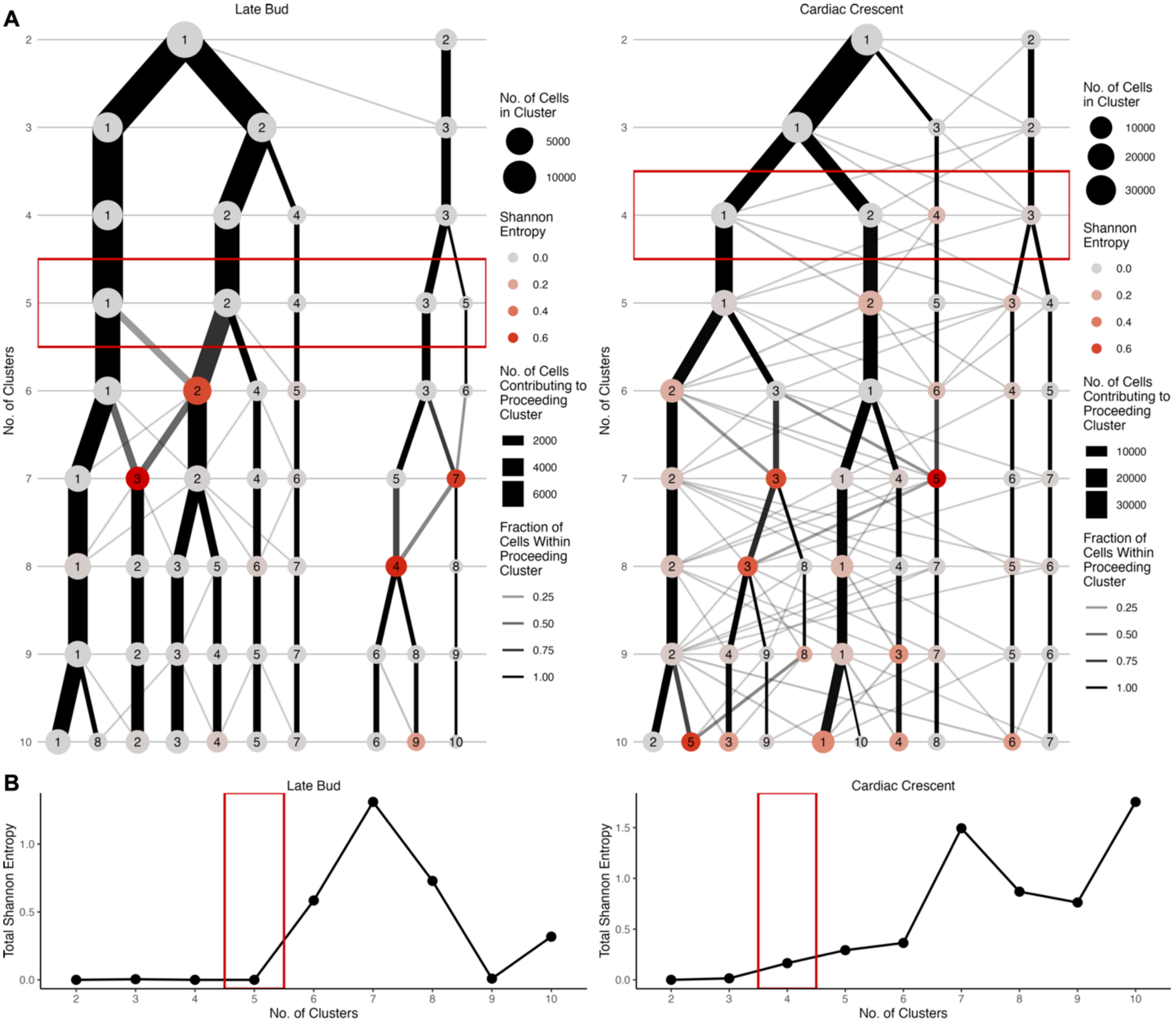

Fig. S5. Example of optimized clustering using clustering tree and Shannon Entropy.

(**A**) Following consecutive clustering of WT cells from, we generated a clustering tree to visualize how cell cluster identities change as the number of clusters consecutively increase. Nodes represent clusters and edges represent cells from preceding clusters. Clusters are stable when a large proportion of cells are derived from a single preceding cluster rather than multiple preceding clusters. We adopted Shannon Entropy as a measure of these proportions as a measure of intra-cluster stability. A low Shannon Entropy represents high intra-cluster stability. Here, WT cells from LB-stage embryos can only be clustered as high as 5 clusters before they can be subclustered into further subclusters. WT cells from CC-stage embryos can only be clustered as high as 4 clusters before they can be subclustered into further clusters. (**B**) Total Shannon Entropy of all clusters among WT cells from LB- or CC-stage embryos at consecutively increasing number of clusters.

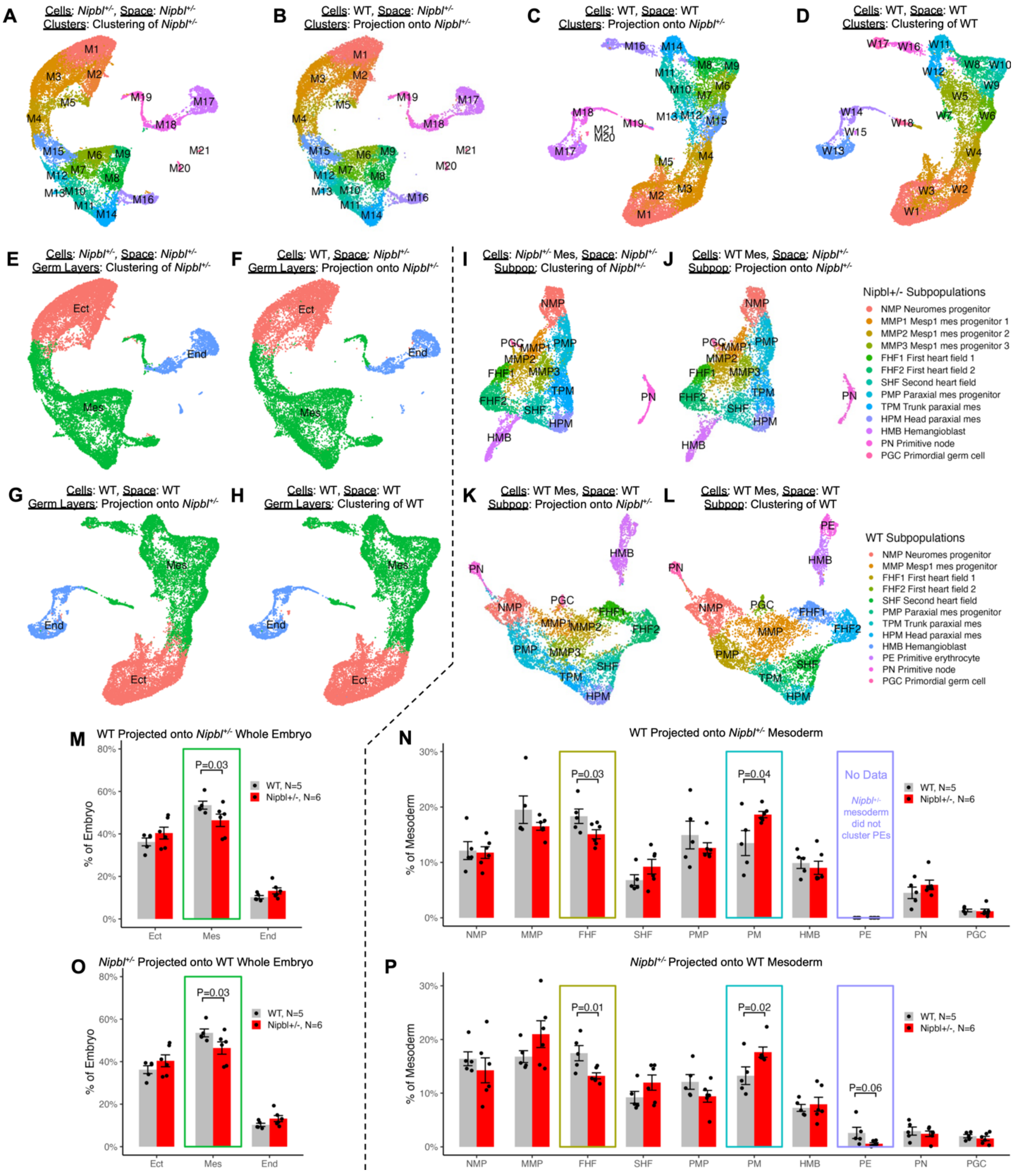
A
Cells: Nipbl+/-, Space: Nipbl+/-
Clusters: Clustering of Nipbl+/-
B
Cells: WT, Space: Nipbl+/-
Clusters: Projection onto Nipbl+/-
C
Cells: WT, Space: WT
Clusters: Projection onto Nipbl+/-
D
Cells: WT, Space: WT
Clusters: Clustering of WT
E
Cells: Nipbl+/-, Space: Nipbl+/-
Germ Layers: Clustering of Nipbl+/-
F
Cells: WT, Space: Nipbl+/-
Germ Layers: Projection onto Nipbl+/-
G
Cells: WT, Space: WT
Germ Layers: Projection onto Nipbl+/-
H
Cells: WT, Space: WT
Germ Layers: Clustering of WT
Ect
Mes
End
I
Cells: Nipbl+/- Mes, Space: Nipbl+/-
Subpop: Clustering of Nipbl+/-
J
Cells: WT Mes, Space: Nipbl+/-
Subpop: Projection onto Nipbl+/-
Nipbl+/- Subpopulations
NMP Neuromes progenitor
MMP1 Mesp1 mes progenitor 1
MMP2 Mesp1 mes progenitor 2
MMP3 Mesp1 mes progenitor 3
FHF1 First heart field 1
FHF2 First heart field 2
SHF Second heart field
PMP Paraxial mes progenitor
TPM Trunk paraxial mes
HPM Head paraxial mes
HMB Hemangioblast
PN Primitive node
PGC Primordial germ cell
K
Cells: WT Mes, Space: WT
Subpop: Projection onto Nipbl+/-
L
Cells: WT Mes, Space: WT
Subpop: Clustering of WT
WT Subpopulations
NMP Neuromes progenitor
MMP Mesp1 mes progenitor
FHF1 First heart field 1
FHF2 First heart field 2
SHF Second heart field
PMP Paraxial mes progenitor
TPM Trunk paraxial mes
HPM Head paraxial mes
HMB Hemangioblast
PE Primitive erythrocyte
PN Primitive node
PGC Primordial germ cell
M
WT Projected onto Nipbl+/- Whole Embryo
% of Embryo
P=0.03
WT, N=5
Nipbl+/-, N=6
N
WT Projected onto Nipbl+/- Mesoderm
% of Mesoderm
P=0.03
P=0.04
No Data
Nipbl+/- mesoderm did not cluster PEs
WT, N=5
Nipbl+/-, N=6
NMP MMP FHF SHF PMP PM HMB PE PN PGC
O
Nipbl+/- Projected onto WT Whole Embryo
% of Embryo
P=0.03
WT, N=5
Nipbl+/-, N=6
Ect Mes End
P
Nipbl+/- Projected onto WT Mesoderm
% of Mesoderm
P=0.01
P=0.02
P=0.06
WT, N=5
Nipbl+/-, N=6
NMP MMP FHF SHF PMP PM HMB PE PN PGC

Fig. S6. Percentage of cells in germ layers and mesodermal subpopulations of LB-stage embryos following projection of WT cells onto clustered *Nipbl*$^{+/-}$ cells.

(**A**) LB-stage *Nipbl*$^{+/-}$ cells in UMAP of *Nipbl*$^{+/-}$ cells colored by cluster after clustering *Nipbl*$^{+/-}$ cells separately from WT. (**B**) LB-stage WT cells in UMAP of *Nipbl*$^{+/-}$ cells colored by clusters found in *Nipbl*$^{+/-}$ cells after projecting WT cells onto *Nipbl*$^{+/-}$ cells. (**C**) LB-stage WT cells in UMAP of WT cells colored by clusters found in *Nipbl*$^{+/-}$ cells after projecting WT cells onto *Nipbl*$^{+/-}$ cells. (**D**) LB-stage WT cells in UMAP of WT cells colored by clusters after clustering WT cells separately from *Nipbl*$^{+/-}$. (**E**) LB-stage *Nipbl*$^{+/-}$ cells in UMAP of *Nipbl*$^{+/-}$ cells colored by germ layer after clustering *Nipbl*$^{+/-}$ cells separately from WT. (**F**) LB-stage WT cells in UMAP of *Nipbl*$^{+/-}$ cells colored by germ layers found in *Nipbl*$^{+/-}$ cells after projecting WT cells onto *Nipbl*$^{+/-}$ cells. (**G**) LB-stage WT cells in UMAP of WT cells colored by germ layers found in *Nipbl*$^{+/-}$ cells after projecting WT cells onto *Nipbl*$^{+/-}$ cells. (**H**) LB-stage WT cells in UMAP of WT cells colored by germ layer after clustering WT cells separately from *Nipbl*$^{+/-}$. (**I**) LB-stage *Nipbl*$^{+/-}$ mesoderm cells in UMAP of *Nipbl*$^{+/-}$ mesoderm colored by cell population after clustering *Nipbl*$^{+/-}$ mesoderm separately from WT. (**J**) LB-stage WT mesoderm cells in UMAP of *Nipbl*$^{+/-}$ mesoderm colored by cell populations found in *Nipbl*$^{+/-}$ mesoderm after projecting WT mesoderm onto *Nipbl*$^{+/-}$ mesoderm. (**K**) LB-stage WT mesoderm cells in UMAP of WT mesoderm colored by cell populations found in *Nipbl*$^{+/-}$ mesoderm after projecting WT mesoderm onto *Nipbl*$^{+/-}$ mesoderm. (**L**) LB-stage WT mesoderm cells in UMAP of WT mesoderm colored by cell population after clustering WT mesoderm separately from *Nipbl*$^{+/-}$. (**M**) Percentage of cells in germ layers from all cells in LB-stage WT and *Nipbl*$^{+/-}$ embryos after projecting WT cells onto *Nipbl*$^{+/-}$ cells. Error bars show standard error of the mean. *P*-values from T-Test. (**N**) Percentage of cells in mesodermal cell populations from all mesoderm cells in LB-stage WT and *Nipbl*$^{+/-}$ embryos after projecting WT mesoderm onto *Nipbl*$^{+/-}$ mesoderm. Error bars show SEM. *P*-values from T-Test. (**O**) Percentage of cells in germ layers from all cells in LB-stage WT and *Nipbl*$^{+/-}$ embryos after projecting *Nipbl*$^{+/-}$ cells onto WT cells. Same as This figure is from Fig. 3B. Error bars show standard error of the mean. *P*-values from T-Test. (**P**) Percentage of cells in mesodermal cell populations from all mesoderm cells in LB-stage WT and *Nipbl*$^{+/-}$ embryos after projecting *Nipbl*$^{+/-}$ mesoderm onto WT mesoderm. This figure is from Fig. 3D. Error bars show SEM. *P*-values from T-Test.

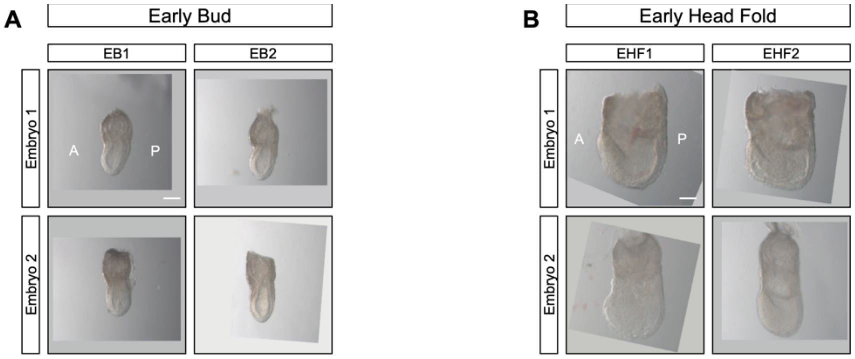

Fig. S7. Images of EB- and EHF-stage WT mouse embryos used for scRNAseq.

Lateral view of WT mouse embryos at (**A**) EB- and (**B**) EHF- stage. A = anterior, P = posterior. Dashed line represents where embryonic tissue was separated from extraembryonic tissue. Scale bar = 100 um.

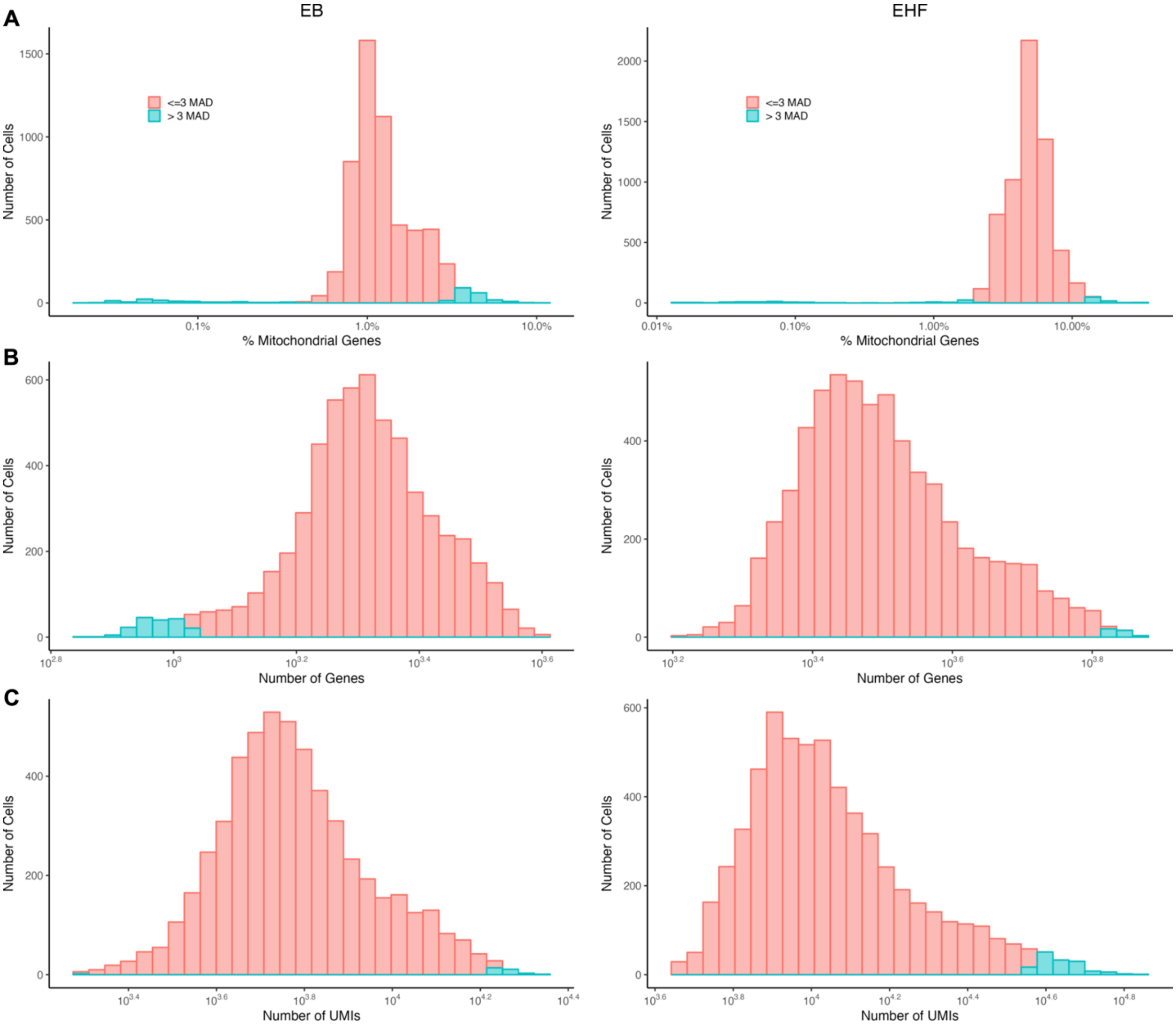

Figure S8. Metrics used to identify low-quality cells and doublets among EB- and EHF-stage embryos.

At each stage, we considered cells that exceeded three median absolute deviations from percent mitochondrial genes expressed, number of genes expressed, and number of transcripts detected per cell low-quality cells and/or doublets and removed them. Histograms of (**A**) percent mitochondrial genes expressed, (**B**) number of genes expressed, and (**C**) number of transcripts detected per cell at EB- and EHF-stage colored by whether cells were less than or equal to 3 median absolute deviations (red) or greater than 3 median absolute deviations (blue). Cells colored in blue were removed.

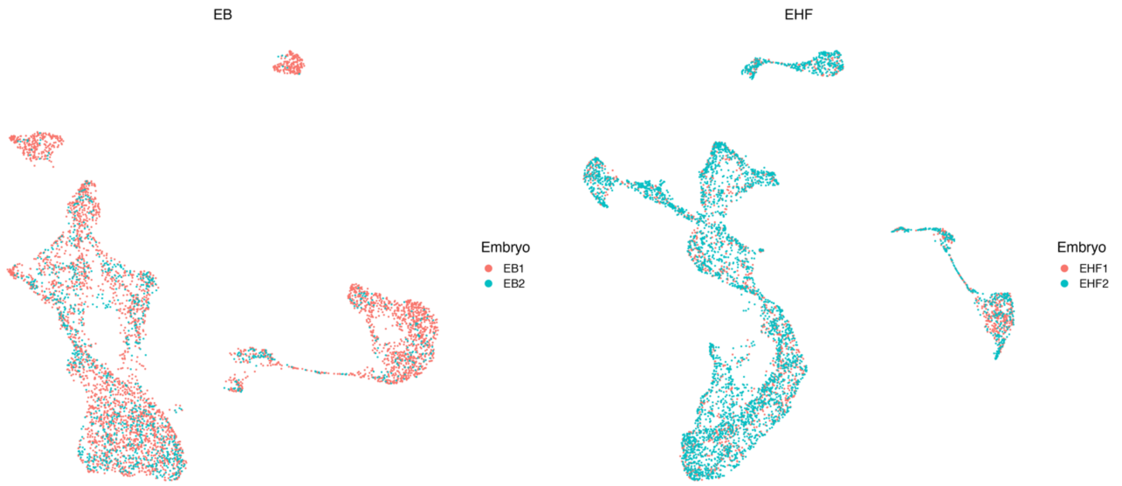

Fig. S9. Batch effect correction and integration of cells of the same stage (EB- or EHF-stage).

We removed batch effects among EB- and EHF-stage embryos of the same stage and integrated embryos of the same stage together. UMAP of EB- and EHF-stage cells colored by embryo.

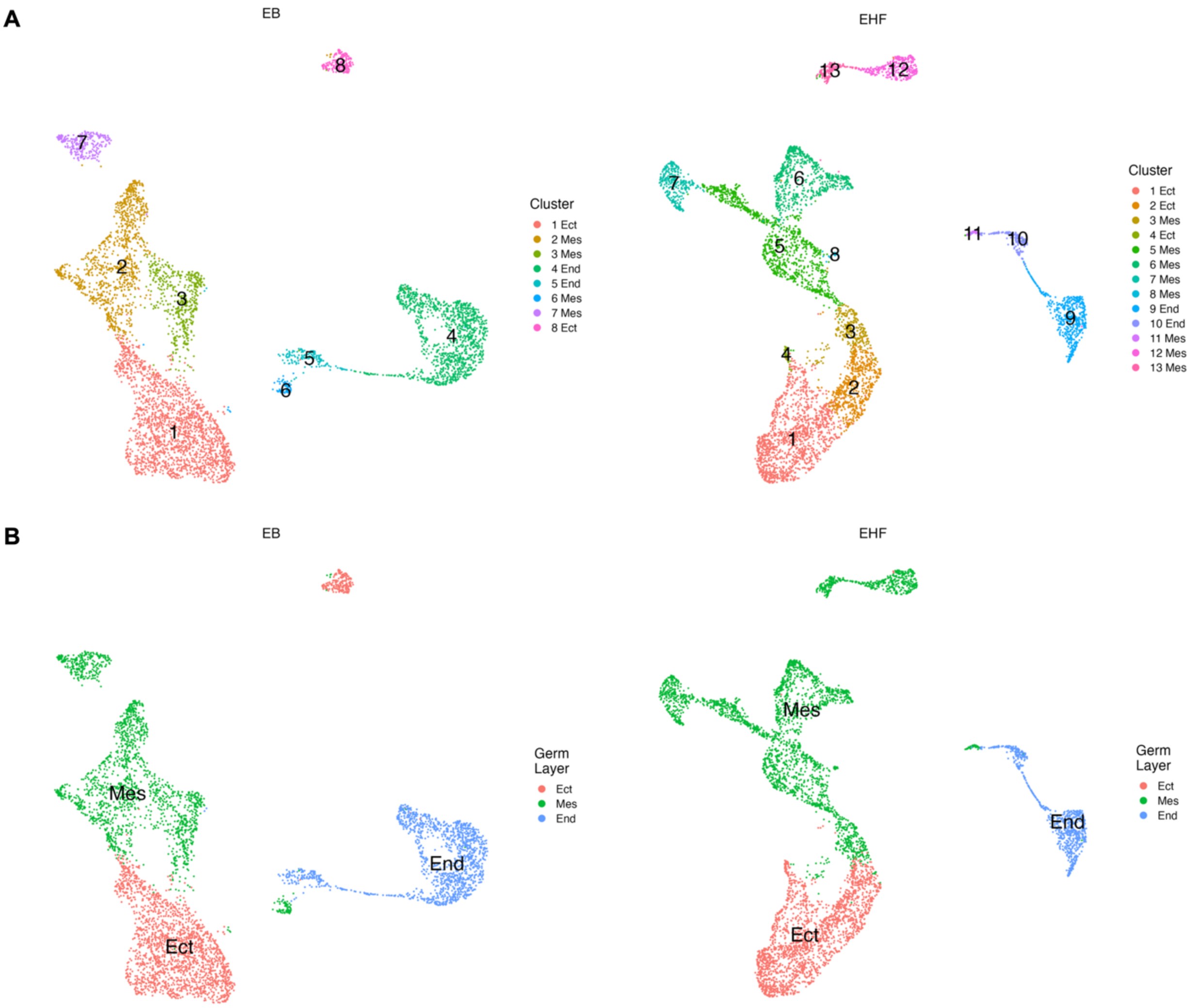

Fig. S10. Clustering of EB- and EHF-stage cells from WT embryos and annotation thereof as either ectoderm, mesoderm, or endoderm.

UMAP of EB- and EHF-stage cells colored by (**A**) cell population or (**B**) germ layer.

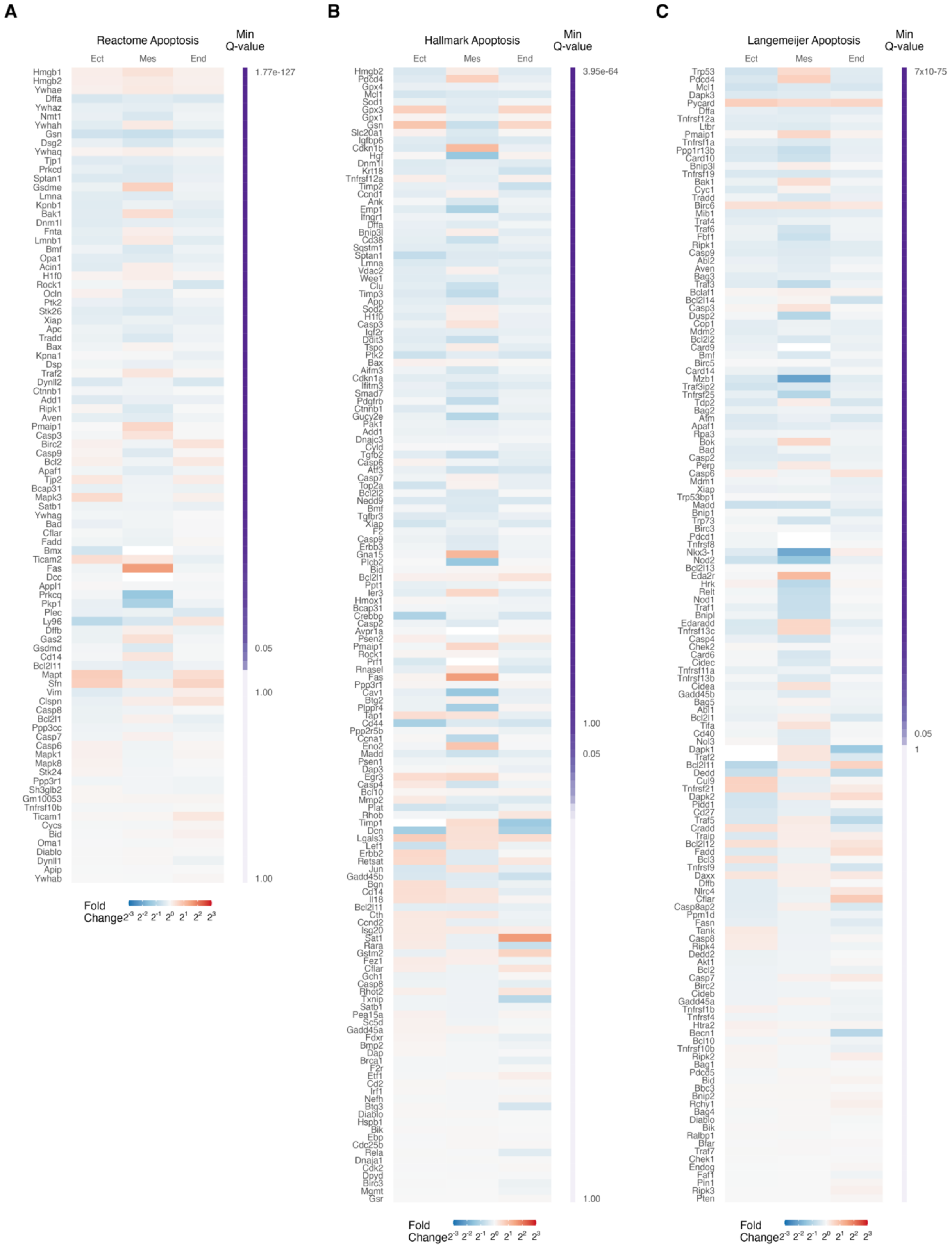
A
Reactome Apoptosis
Min Q-value
Ect
Mes
End
Hmgb1 Hmgb2 Ywhae Dffa Ywhaz Nmt1 Ywhah Gsn Dsg2 Ywhaq Tjp1 Prkcd Sptan1 Gsdme Lmna Kpnb1 Bak1 Dnm1l Fnta Lmnb1 Bmf Opa1 Acin1 H1f0 Rock1 Ocln Ptk2 Stk26 Xiap Apc Tradd Bax Kpna1 Dsp Traf2 Dynll2 Ctnnb1 Add1 Ripk1 Aven Pmaip1 Casp3 Birc2 Casp9 Bcl2 Apaf1 Tjp2 Bcap31 Mapk3 Satb1 Ywhag Bad Cflar Fadd Bmx Ticam2 Fas Dcc Appl1 Prkcq Pkp1 Plec Ly96 Dffb Gas2 Gsdmd Cd14 Bcl2l11 Mapt Sfn Vim Clspn Casp8 Bcl2l1 Ppp3cc Casp7 Casp6 Mapk1 Mapk8 Stk24 Ppp3r1 Sh3glb2 Gm10053 Tnfrsf10b Ticam1 Cycs Bid Oma1 Diablo Dynll1 Apip Ywhab
1.77e-127
0.05
1.00
1.00
Fold Change
2-3 2-2 2-1 20 21 22 23
B
Hallmark Apoptosis
Min Q-value
Ect
Mes
End
Hmgb2 Pdcd4 Gpx4 Mcl1 Sod1 Gpx3 Gpx1 Gsn Slc20a1 Igfbp6 Cdkn1b Hgf Dnm1l Krt18 Tnfrsf12a Timp2 Ccnd1 Ank Emp1 Ifngr1 Dffa Bnip3l Cd38 Sqstm1 Sptan1 Lmna Vdac2 Wee1 Clu Timp3 App Sod2 H1f0 Casp3 Igf2r Ddit3 Tspo Ptk2 Bax Aifm3 Cdkn1a Ifitm3 Smad7 Pdgfrb Ctnnb1 Gucy2e Pak1 Add1 Dnajc3 Cyld Tgfb2 Casp6 Atf3 Casp7 Top2a Bcl2l2 Nedd9 Bmf Tgfbr3 Xiap F2 Casp9 Erbb3 Gna15 Plcb2 Bid Bcl2l1 Ppt1 Ier3 Hmox1 Bcap31 Crebbp Casp2 Avpr1a Psen2 Pmaip1 Rock1 Prf1 Rnasel Fas Ppp3r1 Cav1 Btg2 Plppr4 Tap1 Cd44 Ppp2r5b Ccna1 Eno2 Madd Psen1 Dap3 Egr3 Casp4 Bcl10 Mmp2 Plat Rhob Timp1 Dcn Lgals3 Lef1 Erbb2 Retsat Jun Gadd45b Bgn Cd14 Il18 Bcl2l11 Cth Ccnd2 Isg20 Sat1 Rara Gstm2 Fez1 Cflar Gch1 Casp8 Rhot2 Txnip Satb1 Pea15a Sc5d Gadd45a Fdxr Bmp2 Dap Brca1 F2r Etf1 Cd2 Irf1 Nefh Btg3 Diablo Hspb1 Bik Ebp Cdc25b Rela Dnaja1 Cdk2 Dpyd Birc3 Mgmt Gsr
3.95e-64
1.00
0.05
1.00
Fold Change
2-3 2-2 2-1 20 21 22 23
C
Langemeijer Apoptosis
Min Q-value
Ect
Mes
End
Trp53 Pdcd4 Mcl1 Dapk3 Pycard Dffa Tnfrsf12a Ltbr Pmaip1 Tnfrsf1a Ppp1r13b Card10 Bnip3l Tnfrsf19 Bak1 Cyc1 Tradd Birc6 Mib1 Traf4 Traf6 Fbf1 Ripk1 Casp9 Abl2 Aven Bag3 Traf3 Bclaf1 Bcl2l14 Casp3 Dusp2 Cop1 Mdm2 Bcl2l2 Card9 Bmf Birc5 Card14 Mzb1 Traf3ip2 Tnfrsf25 Tdp2 Bag2 Aifm Apaf1 Rpa3 Bok Bad Casp2 Perp Casp6 Mdm1 Xiap Trp53bp1 Madd Bnip1 Trp73 Birc3 Pdcd1 Tnfrsf8 Nkx3-1 Nod2 Bcl2l13 Eda2r Hrk Relt Nod1 Traf1 Bnipl Edaradd Tnfrsf13c Casp4 Chek2 Card6 Cidec Tnfrsf11a Tnfrsf13b Cidea Gadd45b Bag5 Abl1 Bcl2l1 Tifa Cd40 Nol3 Dapk1 Traf2 Bcl2l11 Dedd Cul9 Tnfrsf21 Dapk2 Pidd1 Cd27 Traf5 Cradd Traip Bcl2l12 Fadd Bcl3 Tnfrsf9 Daxx Dffb Nlrc4 Cflar Casp8ap2 Ppm1d Fasn Tank Casp8 Ripk4 Dedd2 Akt1 Bcl2 Casp7 Birc2 Cideb Gadd45a Tnfrsf1b Tnfrsf4 Htra2 Becn1 Bcl10 Tnfrsf10b Ripk2 Bag1 Pdcd5 Bid Bbc3 Bnip2 Rchy1 Bag4 Diablo Bik Ralbp1 Bfar Traf7 Chek1 Endog Faf1 Pin1 Ripk3 Pten
7x10-75
0.05
1
Fold Change
2-3 2-2 2-1 20 21 22 23

Fig. S11. Expression of genes associated with apoptosis in *Nipbl*$^{+/-}$ embryos relative to WT.

The germ layers of *Nipbl*$^{+/-}$ embryos do not substantially overexpress or underexpress genes associated with apoptosis. Heatmap of fold change in expression in genes from the (**A**) Reactome Apoptosis, (**B**) Hallmark Apoptosis, and (**C**) Langemeijer Apoptosis gene sets in germ layers of *Nipbl*$^{+/-}$ embryos from that of WT embryos. Genes are ordered from top to bottom by minimum *Q*-value. *Q*-value from Bonferroni corrected *P*-value from Mann-Whitney U test.

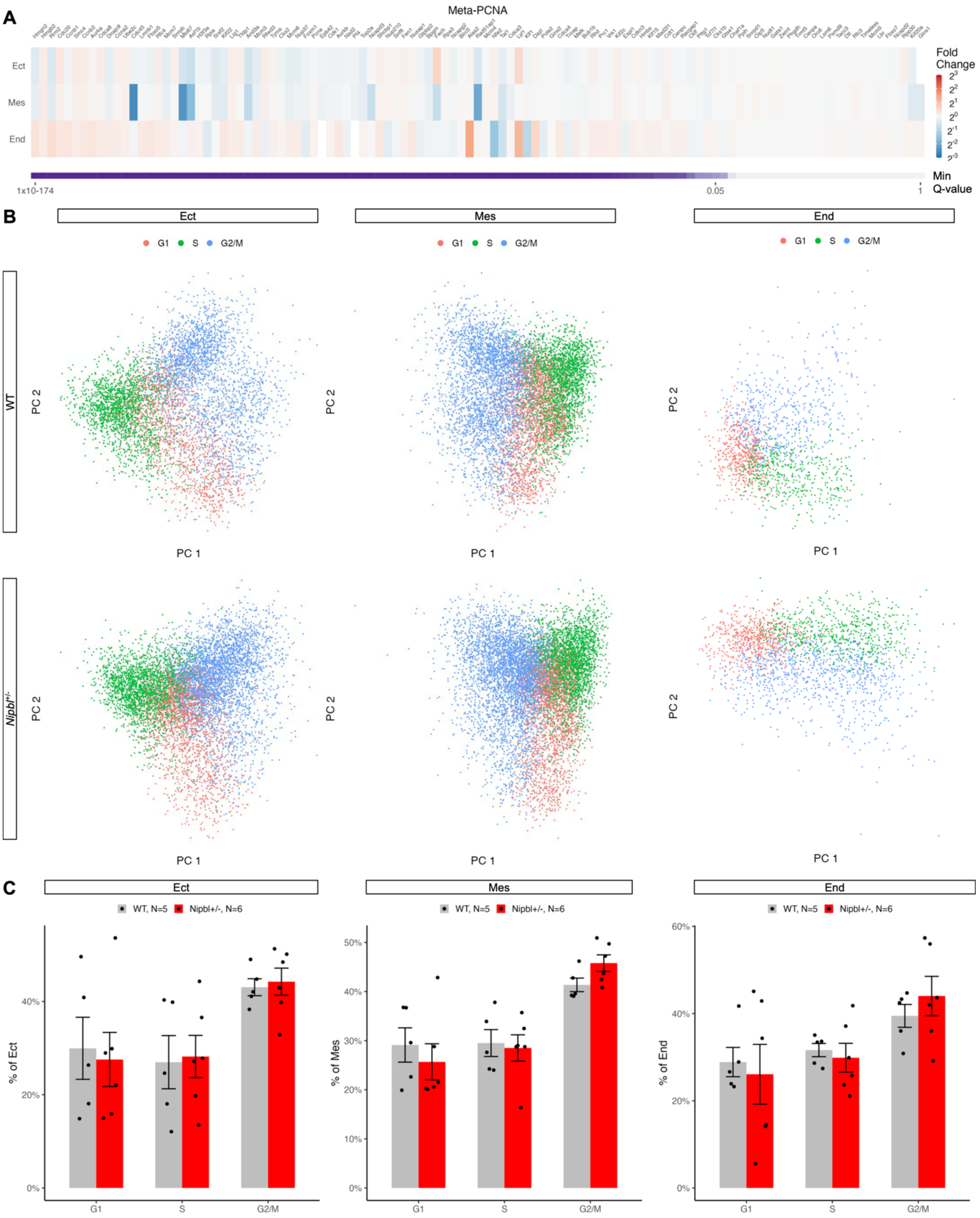
A
Meta-PCNA
Ect
Mes
End
Fold Change
Min Q-value
1x10-174
0.05
1
B
Ect
Mes
End
G1
S
G2/M
WT
Nipbl+/-
PC 2
PC 1
C
Ect
Mes
End
WT, N=5
Nipbl+/-, N=6
% of Ect
% of Mes
% of End
0%
10%
20%
30%
40%
50%
60%
G1
S
G2/M

Fig. S12. Expression of meta-PCNA genes and cell cycle phase composition of *Nipbl*$^{+/-}$ embryos relative to WT.

The germ layers of *Nipbl*$^{+/-}$ embryos do not substantially overexpress or underexpress genes associated with cell proliferation. (**A**) Heatmap of fold change in expression in genes from the meta-PCNA gene set in germ layers of *Nipbl*$^{+/-}$ embryos from that of WT embryos. Genes are ordered from top to bottom by minimum *Q*-value. *Q*-value from Bonferroni corrected *P*-value from Mann-Whitney U test. (**B**) Principal component analysis was performed cells from each germ layer of WT and *Nipbl*$^{+/-}$ embryos using cell cycle phase genes marking S phase and G2/M phase. Seurat was used to assign cells into G1, S, or G2/M phase based on the expression of these markers. Expression of these marker sets are considered anticorrelated. When cells express neither, they are considered to be in G1 phase. In all germ layers, cells from WT and *Nipbl*$^{+/-}$ embryos assigned into all three phases. (**C**) Percentage of cells in each cell cycle phase across all germ layers of WT and *Nipbl*$^{+/-}$ embryos. There was no statistically significant difference in the percentage of cells in each cell cycle phase between that of WT and *Nipbl*$^{+/-}$ embryos. Error bars show standard error of the mean.

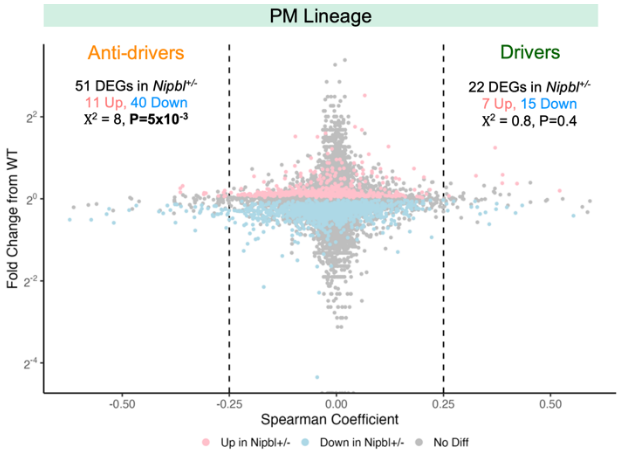

Fig. S13. Paraxial mesoderm lineage of LB-stage *Nipbl*$^{+/-}$ embryos, showing underexpression of paraxial mesoderm anti-drivers. Fold change in expression of genes in mesoderm cells of PM lineage of LB-stage *Nipbl*$^{+/-}$ embryos differentially expressed ($Q < 0.05$, Mann-Whitney U Test) from that of WT embryos along Spearman's Rank Correlation coefficient from Fig. 5E.

Mes

A

		WT Projected onto *Nipbl*$^{+/-}$ **(Reverse Projection)**		
		Up in *Nipbl*$^{+/-}$ (613)	Down in *Nipbl*$^{+/-}$ (2,900)	No Diff in *Nipbl*$^{+/-}$ (12,851)
Nipbl$^{+/-}$ Projected onto WT **(Forward Projection)**	Up in *Nipbl*$^{+/-}$ (604)	**588 (95.9%)**	0 (0.0%)	16 (0.1%)
	Down in *Nipbl*$^{+/-}$ (2,840)	2 (0.3%)	**2,743 (94.6%)**	95 (0.7%)
	No Diff in *Nipbl*$^{+/-}$ (12,920)	23 (3.8%)	157 (5.4%)	**12,740 (99.1%)**

D

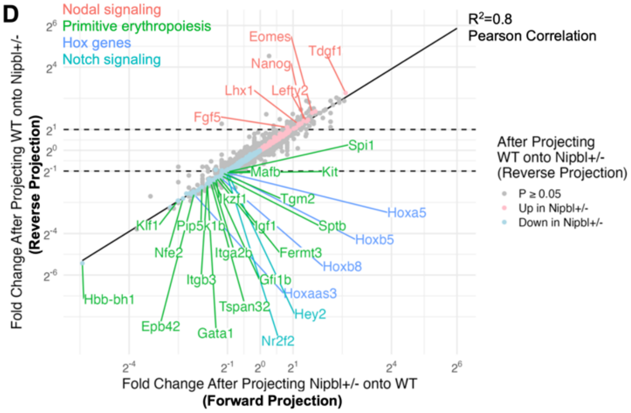

Ect

B

		WT Projected onto *Nipbl*$^{+/-}$ **(Reverse Projection)**		
		Up in *Nipbl*$^{+/-}$ (683)	Down in *Nipbl*$^{+/-}$ (3,655)	No Diff in *Nipbl*$^{+/-}$ (11,947)
Nipbl$^{+/-}$ Projected onto WT **(Forward Projection)**	Up in *Nipbl*$^{+/-}$ (678)	**642 (94.0%)**	6 (0.2%)	30 (0.3%)
	Down in *Nipbl*$^{+/-}$ (3,625)	0 (0%)	**3,463 (94.7%)**	162 (1.4%)
	No Diff in *Nipbl*$^{+/-}$ (11,982)	41 (6.0%)	186 (5.1%)	**11,755 (98.4%)**

E

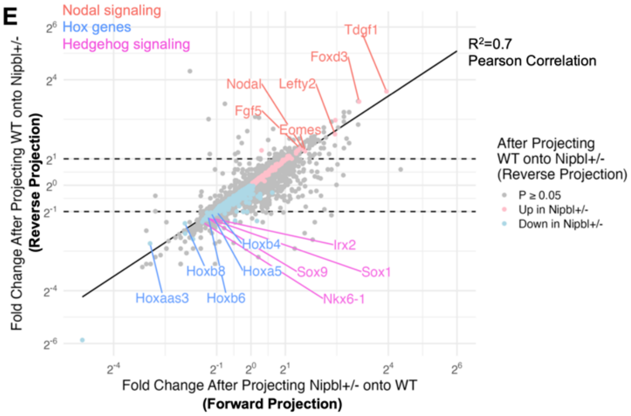

End

C

		WT Projected onto *Nipbl*$^{+/-}$ **(Reverse Projection)**		
		Up in *Nipbl*$^{+/-}$ (488)	Down in *Nipbl*$^{+/-}$ (1,323)	No Diff in *Nipbl*$^{+/-}$ (14,027)
Nipbl$^{+/-}$ Projected onto WT **(Forward Projection)**	Up in *Nipbl*$^{+/-}$ (412)	**369 (75.6%)**	1 (0.1%)	42 (0.3%)
	Down in *Nipbl*$^{+/-}$ (1,116)	3 (0.6%)	**1,019 (77.0%)**	94 (0.7%)
	No Diff in *Nipbl*$^{+/-}$ (14,310)	116 (23.8%)	303 (22.9%)	**13,891 (99.0%)**

F

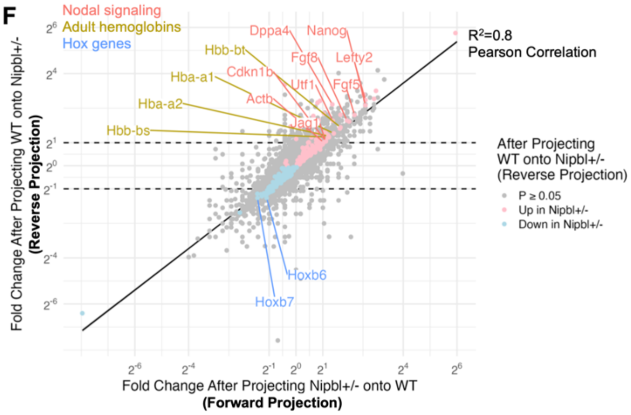

Fig. S14. Differentially expressed genes between germ layers of WT and *Nipbl*$^{+/-}$ embryos, determined following projection of WT cells onto *Nipbl*$^{+/-}$ germ layers.

Percentage of differentially expressed genes ($Q < 0.05$, Mann-Whitney U Test) in (**A**) mesoderm, (**B**) ectoderm, and (**C**) endoderm of LB-stage *Nipbl*$^{+/-}$ embryos resulting from the projection of WT cells onto *Nipbl*$^{+/-}$ germ layers (reverse projection) matching those differentially expressed in the germ layers of *Nipbl*$^{+/-}$ embryos resulting from the projection of *Nipbl*$^{+/-}$ cells onto WT germ layers (forward projection). Fold change in expression of differentially expressed genes ($Q < 0.05$, Mann-Whitney U Test) in the (**D**) mesoderm, (**E**) ectoderm, and (**F**) endoderm of LB-stage *Nipbl*$^{+/-}$ embryos following projection of WT cells onto *Nipbl*$^{+/-}$ germ layers (reverse projection) versus the fold change in expression of the same genes in the germ layers of WT embryos following projection of *Nipbl*$^{+/-}$ cells onto WT germ layers (forward projection).

DEGs in CC-stage *Nipbl*$^{+/-}$ Embryos that are also
DEGs in E9.5 *Nanog* Dox+ Embryos (cf. Ref #*113*)

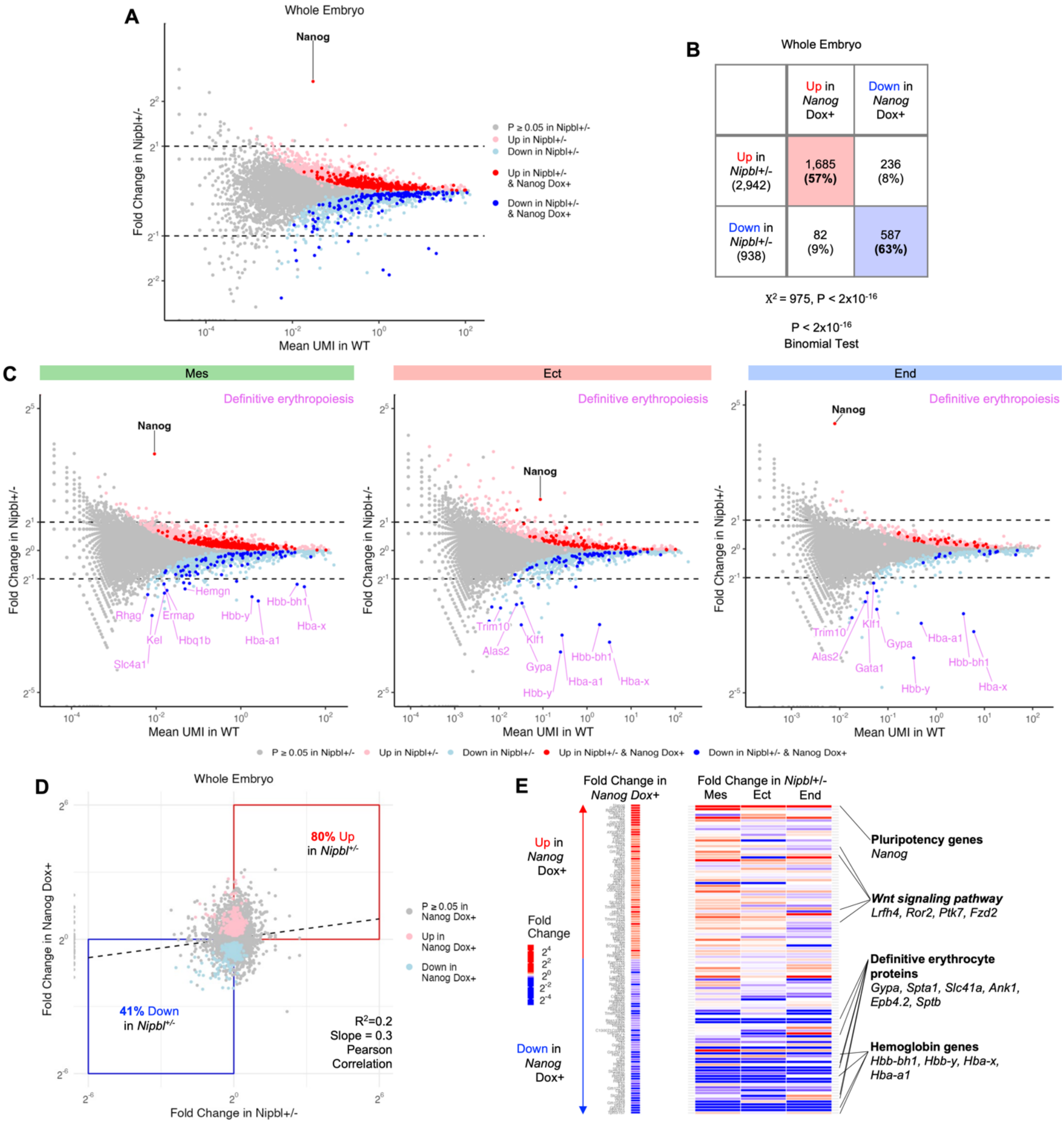

Fig. S15. CC-stage *Nipbl*$^{+/-}$ mice replicate the gene expression changes of *Nanog* overexpression.

(**A**) Fold change in expression of genes in CC-stage *Nipbl*$^{+/-}$ embryos that were differentially expressed ($Q < 0.05$, Mann-Whitney U Test) from WT embryos along with their average expression in WT embryos. Genes that are were also differentially expressed ($Q < 0.05$, T-Test) in E9.5 mouse embryos transgenic for doxycycline inducible expression of *Nanog* (*Nanog* Dox+) from that of WT embryos (*Nanog* Dox-) from *(113)* are colored dark red (upregulated) and dark blue (downregulated). (**B**) Percentage of genes in CC-stage *Nipbl*$^{+/-}$ embryos that were differentially expressed ($Q < 0.05$, Mann-Whitney U Test) from WT embryos, and that were also differentially expressed in the same direction in E9.5 *Nanog* Dox+ embryos. At CC-stage, 57% of overexpressed and 63% of underexpressed genes in *Nipbl*$^{+/-}$ embryos were also overexpressed or underexpressed, respectively, in E9.5 *Nanog* Dox+ embryos. (**C**) Fold change in expression of genes in the germ layers of CC-stage *Nipbl*$^{+/-}$ embryos that were differentially expressed ($Q <$ 0.05, Mann-Whitney U Test) from WT embryos along with their average expression in WT embryos. Genes that are were also differentially expressed ($Q < 0.05$, T-Test) in E9.5 *Nanog* Dox+ embryos are colored dark red and dark blue. At CC-stage, all germ layers of *Nipbl*$^{+/-}$ embryos showed downregulation of genes associated with definitive erythropoiesis, encompassing adult hemoglobins including *Hbb-y, Hba-a1,* and *Hba-x* (data S41, data S42, and data S43). (**D**) Fold change in expression of genes in CC-stage *Nipbl*$^{+/-}$ embryos versus their fold change in E9.5 *Nanog* Dox+ embryos. Genes differentially expressed ($Q < 0.05$, T-Test) in E9.5 *Nanog* Dox+ embryos are colored dark red and dark blue. At CC-stage, the slope of the regression line comparing misexpression in *Nipbl*$^{+/-}$ and E9.5 *Nanog* Dox+ embryos was found to be 0.3 (panel D). Additionally, at CC-stage, *Nipbl*$^{+/-}$ embryos upregulated 80% of the same genes that were overexpressed in E7.5 *Nanog* Dox+ embryos and downregulated 41% of the same genes underexpressed in E7.5 *Nanog* Dox+ embryos. (**E**) Heatmap of fold change in expression in germ layers of CC-stage *Nipbl*$^{+/-}$ embryos from that of WT embryos of the most differentially expressed genes from E9.5 *Nanog* Dox+ embryos from that of Nanog Dox- embryos (lowest Q-values from T-Test) from *(113)*. Genes are ordered from top to middle (overexpressed in *Nanog* Dox+ embryos) and bottom to middle (underexpressed in *Nanog* Dox+ embryos) by lowest to higher Q-value. Underexpressed genes in *Nanog* Dox+ embryos were strongly mirrored in the germ layers of CC-stage *Nipbl*$^{+/-}$ embryos (panel E). In CC-stage *Nipbl*$^{+/-}$ embryos, only a few overexpressed genes observed in *Nanog* Dox+ embryos were replicated, the most prominent of which was *Nanog* itself.

Table S1. Number of cells, transcripts, and genes captured per LB- and CC-stage WT and *Nipbl*$^{+/-}$ embryos.

scRNAseq of LB- and CC-stage WT and *Nipbl*$^{+/-}$ embryos captured thousands of cells per embryo, thousands of transcripts per cell, and detected thousands of genes per cell. Table shows stage of each embryo, genotype of each embryo, number of cells captured per embryo, median number of UMIs captured per cell, and median number of genes detected per cell.

Stage	Genotype	Sample	Cells	Median UMIs/Cell	Median Genes/Cell
LB	WT	LB1	2,445	17,998	4,242
LB	WT	LB2	1,247	17,011	3,947
LB	WT	LB3	2,537	16,476	3,830
LB	WT	LB4	7,119	18,624	3,940
LB	WT	LB5	2,321	18,413	4,076
LB	WT	LB1-LB5	15,669	18,078	4,001
LB	*Nipbl*$^{+/-}$	LB6	1,700	9,953.5	2,997
LB	*Nipbl*$^{+/-}$	LB7	1,411	18,111	4,110
LB	*Nipbl*$^{+/-}$	LB8	4,218	11,875	3,168
LB	*Nipbl*$^{+/-}$	LB9	3,969	15,752	3,702
LB	*Nipbl*$^{+/-}$	LB10	3,431	17,123	3,847
LB	*Nipbl*$^{+/-}$	LB11	4,840	18,910.5	4,000.5
LB	*Nipbl*$^{+/-}$	LB6-LB11	19,569	16,424	3,780
LB	*WT & Nipbl*$^{+/-}$	LB1-LB11	35,238	17,300	3,894
CC	WT	CC1	1858	15,682	3,796
CC	WT	CC2	3537	14,967	3,711
CC	WT	CC3	4550	13,788	3,609
CC	WT	CC4	5202	14,782	3,549
CC	WT	CC5	7448	14,882	3,453
CC	WT	CC6	7759	14,547	3,438
CC	WT	CC7	4767	15,386	3,716
CC	WT	CC8	6573	15,797	3,652
CC	WT	CC1-CC8	41,694	14,916	3,580
CC	*Nipbl*$^{+/-}$	CC9	2104	15,861	3,837
CC	*Nipbl*$^{+/-}$	CC10	2197	14,968	3,643
CC	*Nipbl*$^{+/-}$	CC11	669	16,370	3,994
CC	*Nipbl*$^{+/-}$	CC12	1614	15,140	3,750
CC	*Nipbl*$^{+/-}$	CC13	2025	15,525	3,797
CC	*Nipbl*$^{+/-}$	CC14	3683	13,302	3,493
CC	*Nipbl*$^{+/-}$	CC15	4919	14,986	3,656
CC	*Nipbl*$^{+/-}$	CC16	8236	15,888	3,699
CC	*Nipbl*$^{+/-}$	CC9-16	25,447	15,122	3,677
CC	*WT & Nipbl*$^{+/-}$	CC1-CC16	67,141	14,997	3,618
LB & CC	*WT & Nipbl*$^{+/-}$	LB1-LB11 & CC1-CC16	102,379	15,429	3,676

Table S2. Differential overexpression of markers of germ layer identity by cell populations in LB- and CC-stage embryos.

Stage	Cell Population	Markers	Germ Layer
LB	1	*Dnmt3b, Utf1 (31), Pou3f1 (33), Eras, Sox2 (32), Epcam*	Ectoderm
LB	2	*Gbx2, Pou5f1, Cdx2, Cdkn1a*	Ectoderm
LB	3	*Tfap2a, Gata3, Tfap2c*	Ectoderm
LB	4	*T (34), Mixl1, Wnt3a, Nkx1-2, Pou5f1, Nkx2-1*	Mesoderm
LB	5	*Mesp1, Hand1 (35), Foxf1, Fn1, Snai1, Isl1, Bmp4, Vim (36)*	Mesoderm
LB	6	*Hoxb1, Tbx6, Dll3, Dll1, Lef1, Frzb*	Mesoderm
LB	7	*Dppa3, Psmb8, Ifitm3, Tapbp, Irf1*	Mesoderm
LB	8	*Smarcd3, Myl7, Gata6, Cdkn1c, Mef2c, Meis1*	Mesoderm
LB	9	*Foxc2, Twist1 (37), Fn1, Hmga2*	Mesoderm
LB	10	*Foxc2, Twist1 (37), Pitx2, Foxc1, Cd24a, Hmga2, Prrx2 (38)*	Mesoderm
LB	11	*Hand1 (35), Bmp4, Foxf1, Msx2*	Mesoderm
LB	12	*Hand1 (35), Pitx1, Bambi, Foxf1, Bmp4*	Mesoderm
LB	13	*Cubn, Apob, Amn, Apom, Apoa4, Dab2, Pla2g12b*	Endoderm
LB	14	*Cldn6, Krt18, Krt8, Foxa2, Sox17, Cer1, Cldn7*	Endoderm
LB	15	*Tex19.1, Dppa4, Zfp4*	Ectoderm
LB	16	*Tal1, Kdr, Lmo2, Etv2*	Mesoderm
LB	17	*Gata1, Tal1, Lmo2, Hbb-bh1, Gfi1b*	Mesoderm
LB	18	*1110017D15Rik, Fam183b, Tekt1, Dynlrb2*	Mesoderm
CC	1	*Lef1, Tbx6, Dll3, Hes7, Hoxb1, Dll1, Cdx1*	Mesoderm
CC	2	*Hoxd9, Lef1, Msx1, Hoxb6, Hoxb8, Nkd1, Msx2, Hoxb9*	Mesoderm
CC	3	*Mdk, Vim (36), Hand2, Arg1, Fn1, Nrp1, Pdgfra, Ppp3ca*	Mesoderm
CC	4	*Dppa3, Psmb8, Ifitm3, Gdf3, Psme1, Irf1*	Mesoderm
CC	5	*Twist1 (37), Prrx2 (38), Foxc2, Foxd1, Prrx1*	Mesoderm
CC	6	*Meis1, Gata6, Isl1, Arg1, Mef2c, Meis2, Smarcd3, Hand2, Nr2f2, Irx3*	Mesoderm
CC	7	*Foxc2, Foxc1, Prrx2 (38), Twist1 (37)*	Mesoderm
CC	8	*Arg1, Pkm, Tubb5, Trim28, Eef1a1, Sox2 (32), Actb, Igf2bp1, Pou3f1 (33)*	Ectoderm
CC	9	*Acta2, Krt8, Krt18, Tagln*	Mesoderm
CC	10	*Phlda2, Cdkn1c, Peg10, Slc38a4*	Mesoderm
CC	11	*Etv2, Egfl7, Ramp2, Kdr, Fli1, Hhex, Tal1, Lmo2, Flt1, Rasip1, Esam*	Mesoderm
CC	12	*Myl7, Actc1, Tnnt2, Myl4*	Mesoderm
CC	13	*Pou3f1 (33), Sox2 (32), En1, Pax2, Dnmt3b*	Ectoderm
CC	14	*Hoxa1, Pax6, Sox2 (32), Meis2, Sox3*	Ectoderm
CC	15	*Six3, Hesx1, Pou3f1 (33), Otx2, Sox2 (32)*	Ectoderm
CC	16	*Pax3, Sox9, Foxd3*	Ectoderm
CC	17	*Ptch1, Foxa2, Sox2 (32), Nkx6-1, Pou3f1 (33), Sfrp1*	Ectoderm
CC	18	*Cdx2, Nr6a1, Pou5f1, Ccnd1*	Mesoderm
CC	19	*Fst, Fgf8, T (34), Etv4*	Mesoderm
CC	20	*Msx1, Cdx4, Cdx2, Fgf8, Etv4, T (34), Evx1, Klf5, Nkx1-2*	Mesoderm
CC	21	*Epcam, Cldn7, Rab25, Cldn6, Krt18, Krt8*	Ectoderm
CC	22	*Krt18, Epcam, Krt8*	Endoderm
CC	23	*Shh, T (34), Nog, Foxa2*	Mesoderm
CC	24	*Apob, Amn, Afp, Apom, Rbp4, Ttr (39), Apoa4, Apoa1, Cubn*	Endoderm
CC	25	*Ttr (39), Rbp4, Apoa1, Apom*	Endoderm
CC	26	*Hba-x, Hbb-bh1, Hba-a1, Hba-a2*	Mesoderm

Table S3. Differential overexpression of markers of biological identity by endodermal cell populations in LB- and CC-stage embryos.

Stage	Cell Population	Markers	Biological Identity
LB	13	*Hnf4a (42), Tbx3 (43), S100a1, Rbp4, Cubn, Mt1, Slc2a2, Apoa2, Ctsb, Aldob, Apom, Lrp2, Cltc, Aprt*	Extraembryonic endoderm
LB	14	*Sox4 (40) Mdk, Tead2, Cer1, Ccnd2, Dkk1, Sfrp1, Foxa2 (41), Bambi, Fzd2, Ccnd1*	Definitive endoderm progenitor
CC	22	*Krt18, Slc2a1, Cd24a, Epcam, Ptma, Hmgn1, Tpt1, Rps2, Bsg, Rps23, Cfl1, Ppia,*	Definitive endoderm progenitor
CC	24	*Esx1 (44), Ldha, Fam162a, Eno1, Slc16a3, Pgk1, Tubb5, Pkm, Aldoa, Hspa8*	Visceral endoderm
CC	25	*Rbp4, Apoa1, Ttr, Apom*	Extraembryonic endoderm

Table S4. Differential overexpression of markers of biological identity by ectodermal cell populations in LB- and CC-stage embryos.

Stage	Cell Population	Markers	Biological Identity
LB	1	*Dnmt3b, Pou3f1 (33), Otx2 (45), Utf1, Eras, Irx3, Hesx1, Sox2*	Ectoderm progenitor
LB	2	*Cdx1 (46), Nkx1-2 (47), Cdx2, Fst, Gbx2, Pou5f1, Cdkn1a, Fgf8*	Neural ectoderm progenitor
LB	3	*Id1 (45) Msx2 (45), Msx1 (45), Tfap2a*	Surface ectoderm
LB	15	*Elf5 (49), Dppa4, Tex19.1, Grem2, Bmp8b, Sox7, Tead4 (50), Esrrb, Dppa2, Gata6, Nog, Zfp42, Sox17*	Extraembryonic ectoderm
CC	8	*Sox2 (60), Ezr (61), Arg1, Fn1, Twist1, Foxc2, Prrx2, Hmga2*	Spinal neural plate
CC	13	*En1 (54), Pax2 (55), Pou3f1, Zic3, Zeb2, Wnt1, Sox2, Pax5, Sox3*	Mesencephalic neural plate
CC	14	*Hoxa1 (56), Hes3 (57), Mdk, Meis2, Tshz1, Pax6, Pou5f1, Ptn*	Rhombencephalic neural plate
CC	15	*Six3 (52), Hesx1 (53), Pou3f1, Otx2, Lhx5, Fezf1, Rax*	Prosencephalic neural plate
CC	16	*Pax3 (62), Foxd3 (63), Sox9*	Neural crest
CC	17	*Ptch1, Foxa2 (58), Nkx2-9 (59), Nkx6-1, Cfc1, Sox2, Gli1, Lefty1, Ccnd1, Sfrp1*	Midline neural plate
CC	21	*Krt8, Krt18, Epcam*	Surface ectoderm

Table S5. Differential overexpression of markers of biological identity by mesodermal cell populations in LB- and CC-stage embryos.

Stage	Cell Population	Markers	Biological Identity
LB	4	*Pou5f1, T (34), Nkx1-2 (47), Epcam, Cdh1, Wnt3a, Mixl1, Cdx2, Mgst1, Dnmt3b, Cdkn1a*	Neuromesodermal progenitor
LB	5	*Mesp1 (64), Hand1, Isl1, Cdx2, Evx1*	*Mesp1*-expressing mesoderm progenitor
LB	6	*Tbx6 (67), Meis2 (68), Hoxb1, Dll1, Dll3, Lef1, Frzb*	Paraxial mesoderm progenitor
LB	7	*Dppa3, Ifitm3, Psmb8, Tfap2c (77), Msx1 (78), Psme2, Psme1, Irf1, Tapbp*	Primordial germ cell
LB	8	*Id2 (65), Smarcd3, Myl7, Cdkn1c, Mef2c (66), Gata6*	Second heart field
LB	9	*Foxc2 (69), Tcf15 (70), Twist1, Cd24a, Tcf7l1, Lef1*	Trunk paraxial mesoderm
LB	10	*Alx1 (70), Tcf15 (70), Cd24a, Pitx2, Twist1, Foxc2, Foxc1, Prrx2, Six1, Bmp7, Jag1*	Head paraxial mesoderm
LB	11	*Hand1 (35), Bmp4, Msx2, Foxf1*	First heart field 2
LB	12	*Pitx1, Hand1 (35), Bambi, Krt8, Msx1, Krt18, Foxf1, Bmp4, Col1a1, Msx2, Cdx2*	First heart field 1
LB	16	*Kdr, Tal1 (72), Lmo2, Etv2 (73)*	Hemangioblast
LB	17	*Gata1 (72), Hbb-bh1, Tal1 (72), Runx1*	Primitive erythrocyte
LB	18	*Noto (75), Foxj1 (76), 1110017D15Rik, Tekt1, Pifo, Enkur, Fam183b*	Primitive node
CC	1	*Tbx6, Dll3, Lef1, Hes7, Dll1, Hoxb1, Cdx1*	Trunk paraxial mesoderm
CC	2	*Mesp1 (64), Hand1, Msx1, Lef1, Msx2, Cdx2, Nkd1, Sall4, Tbx3*	*Mesp1*-expressing mesoderm progenitor 1
CC	3	*Mesp1 (64), Meis1, Meis2, Hoxa1, Tshz1, Hoxa5, Foxp1, Pbx3*	*Mesp1*-expressing mesoderm progenitor 2
CC	4	*Dppa3, Psmb8, Ifitm3, Irf1, Dnd1, Gdf3, Psme1, Parp12, Psme2, Nanos3, Ifitm2, Tapbp*	Primordial germ cell
CC	5	*Twist1, Prrx2, Foxc2, Foxd1, Prrx1*	Head paraxial mesoderm
CC	6	*Meis1, Gata6, Isl1, Irx3, Meis2, Irx5*	Second heart field
CC	7	*Tcf15 (70), Meox1 (82), Foxc2, Ptn, Pax3, Foxc1, Cadm1, Mdk, H3f3a, Epb41l3, Foxd1, Fbn2, Six1, Ncam1, Notch1, Prrx2, Efnb2, Pdgfra, Twist1, Zic2*	Presomitic mesoderm
CC	9	*Krt18, Krt8, Tagln, Hand1, Acta2, Sparc, Cnn2, Vim, Bmp4, Csrp2, Nrp1, Serpinh1, Myl6*	First heart field 2
CC	10	*Hand1, Bambi, Bmp4, Twist1*	First heart field 1
CC	11	*Etv2, Ramp2, Egfl7, Kdr, Fli1, Hhex, Rasip1, Flt1, Tal1, Esam, Lmo2*	Hemangioblast
CC	12	*Mef2c (80), Nkx2-5 (81), Myl7, Actc1, Tnnt2, Myl4*	Cardiomyocyte
CC	18	*Cdx2, Sox2, Nr6a1, Pou5f1*	Neuromesodermal progenitor
CC	19	*T, Fgf8, Fst, Etv4, Fgf17*	Paraxial mesoderm progenitor
CC	20	*Tlx2 (79), Msx1, Cdx4, Cdx2, Evx1os, Etv4, Fgf8, T (34)*	Lateral plate mesoderm progenitor
CC	23	*Shh, Foxa2, Nog, Krt18*	Primitive node
CC	26	*Hba-x, Hbb-bh1, Hba-a1, Hba-a2*	Primitive erythrocyte

Table S6. Differential overexpression of markers of germ layer identity by cell populations in LB-stage *Nipbl*$^{+/-}$ embryos.

Stage	Cell Population	Markers	Germ Layer
LB	1	*Dnmt3b, Utf1 (31)*	Ectoderm
LB	2	*Dlx5, Tfap2a*	Ectoderm
LB	3	*Pou5f1, Dnmt3b, Hes3*	Ectoderm
LB	4	*Cdx1, Mixl1, Pou5f1*	Mesoderm
LB	5	*Msx1, Tfap2a*	Ectoderm
LB	6	*Foxc2, Lefty2*	Mesoderm
LB	7	*Mesp1, Rspo3, Isl1, Wnt2*	Mesoderm
LB	8	*Myl7, Smarc3d, Cdkn1c, Gata6, Phlda2, Mef2c*	Mesoderm
LB	9	*Tcf15, Foxc2*	Mesoderm
LB	10	*Hand1 (35), Bmp4*	Mesoderm
LB	11	*Hoxb6, Hand1 (35), Hoxd9, Pitx1, Foxf1*	Mesoderm
LB	12	*Mesp1, Mixl1*	Mesoderm
LB	13	*Dppa3, Dnd1*	Mesoderm
LB	14	*Hand1 (35), Krt18, Krt8, Foxf1, Bmp4,*	Mesoderm
LB	15	*Tbx6, Hoxb1*	Mesoderm
LB	16	*Tal1, Lmo2*	Mesoderm
LB	17	*Ttr, Apoa1*	Endoderm
LB	18	*Slc116a1, Emb*	Endoderm
LB	19	*Pifo, Cfap126*	Mesoderm
LB	20	*Tex19.1, Dppa4*	Endoderm
LB	21	*Hs3st1, Srgn*	Endoderm

Table S7. Differential overexpression of markers of biological identity by mesodermal cell populations in LB-stage *Nipbl*$^{+/-}$ embryos.

Stage	Cell Population	Markers	Biological Identity
LB	4	*Pou5f1, T (34)*	Neuromesodermal progenitor
LB	6	*Foxc2 (69), Tcf15 (70)*	Trunk paraxial mesoderm
LB	7	*Mesp1 (64), Rspo3, Hoxb1, Wnt2, Isl1, Meis2 (68)*	Mesp1-expressing mesoderm progenitor 3
LB	8	*Myl7, Smarcd3, Gata6, Cdkn1c, Mef2c (66)*	Second heart field
LB	9	*Otx2, Cer1*	Head paraxial mesoderm
LB	10	*Bmp4, Hand1 (35)*	Mesp1-expressing mesoderm progenitor 2
LB	11	*Hoxb6, Hoxd9*	First heart field 2
LB	12	*Mixl1, Mesp1 (64)*	Mesp1-expressing mesoderm progenitor 1
LB	13	*Dppa3, Psmb8, Dnd1, Nanos3, Ifitm3, Prdm14*	Primordial germ cell
LB	14	*Krt18, Krt8*	First heart field 2
LB	15	*Tbx6 (67), Rbp1, Hoxb1, Aldh1a2*	Paraxial mesoderm progenitor
LB	16	*Tal1 (72), Lmo2*	Hemangioblast
LB	19	*Pifo, Cfap126, Foxj1 (76)*	Primitive node

Table S8. Number of cells, transcripts, and genes captured by scRNAseq of EB- and EHF-stage WT embryos.

scRNA-seq of EB- and EHF-stage of WT mouse embryos captured thousands of cells per embryo, thousands of transcripts per cell, and detected thousands of genes per cell. Table shows genotype of each embryo, number of cells captured per embryo, median number of UMIs captured per cell, and median number of genes detected per cell.

Embryo(s)	Genotype	Cells	Median UMIs/Cell	Median Genes/Cell
EB1	WT	4109	5693	2064
EB2	WT	1185	5943	2120
EB1-EB2	WT	5294	5740	2073.5
EHF1	WT	1969	11208	3271
EHF2	WT	3839	9587	2891
EHF1-EHF2	WT	5808	10162	3030

Table S9. Differential overexpression of markers of germ layer identity by cell populations in EB- and EHF-stage embryos.

Stage	Cell Population	Markers	Germ Layer
EB	1	*Pou5f1, Dnmt3b, Epcam, Utf1, Pkm*	Ectoderm
EB	2	*Vim, Hand1, Hmga2, Fn1, Actb, Fgf3, Tpm4, Myl7, Cald1*	Mesoderm
EB	3	*Lhx1, Eomes, Fgf3, Snai1, Fn1, Mixl1*	Mesoderm
EB	4	*Apom, Apoa1, Dab2, Ttr, Apob, Amn*	Endoderm
EB	5	*Cer1, Gsc, Lhx1, Sox17, Fgf5, Foxa2*	Endoderm
EB	6	*Foxj1, 1110017D15Rik, Tekt1, Pifo, Cfap126*	Mesoderm
EB	7	*Lmo2, Fgf3, Tal1, Runx1, Fli1, Kdr*	Mesoderm
EB	8	*Elf5, S100a6, Nr0b1, Anxa5, Rhox5, Trap1a, Tex19.1, Hspb1, Tfap2c, Dnmt3l, Dppa4, Cd9, Lgals1, Zfp42*	Ectoderm
EHF	1	*Dnmt3b, Gapdh, Epcam, Sox2, Pou3f1*	Ectoderm
EHF	2	*Pou5f1, Gbx2, Sox3, Cdx2*	Ectoderm
EHF	3	*T, Fst, Nkx1-2, Cdx2, Pou5f1, Wnt3a, Fgf8*	Mesoderm
EHF	4	*Tacstd2, Wnt6, Wnt4, Lrp2, Lrpap1, Krt18, Krt8, Wt1*	Ectoderm
EHF	5	*Lef1, Dll1, Pcdh19, Dll3, Tbx6, Cdh2*	Mesoderm
EHF	6	*Hmga2, Twist1, Prrx2, Foxc2*	Mesoderm
EHF	7	*Hand1, Krt18, Krt8, Foxf1, Tagln, Vim, Serpinh1, Ppic, Cnn2, Tpm1, Nrp1, Acta2, Bmp4, Myl6*	Mesoderm
EHF	8	*Dppa3, Ifitm3, Gdf3, Dnd1, Parp9, Tfap2c, Klf2, Parp12, Prdm1*	Mesoderm
EHF	9	*Amn, Apom, Apoa1, Cubn, Apob, Ttr*	Endoderm
EHF	10	*Krt8, Krt18, Sox17, Foxa1, Foxa2*	Endoderm
EHF	11	*Foxj1, 1110017D15Rik, Dynlrb2, Cfap52, Fam183b, Cfap126*	Mesoderm
EHF	12	*Lmo2, Gata1, Hbb-bh1, Runx1*	Mesoderm
EHF	13	*Kdr, Fli1, Etv2, Lmo2*	Mesoderm

Table S10. Expression of *Nanog* in LB- and CC-stage embryos.

Genotype = genotype of embryo, Embryo = embryo identifier, Cell_Type = cell type in embryo, Nanog = normalized transcript counts for *Nanog* in cell type.

Stage	Genotype	Embryo	*Nanog*
Late bud	Wildtype	LB1	0.05051302
Late bud	Wildtype	LB2	0.11359867
Late bud	Wildtype	LB3	0.08
Late bud	Wildtype	LB4	0.10866373
Late bud	Wildtype	LB5	0.07430341
Late bud	*Nipbl*$^{+/-}$	LB6	0.3121175
Late bud	*Nipbl*$^{+/-}$	LB7	0.20864662
Late bud	*Nipbl*$^{+/-}$	LB8	0.24953096
Late bud	*Nipbl*$^{+/-}$	LB9	0.18756219
Late bud	*Nipbl*$^{+/-}$	LB10	0.16984127
Late bud	*Nipbl*$^{+/-}$	LB11	0.19027661
Cardiac crescent	Wildtype	CC1	0.00668338
Cardiac crescent	Wildtype	CC2	0.01766304
Cardiac crescent	Wildtype	CC3	0.01175446
Cardiac crescent	Wildtype	CC4	0.00583771
Cardiac crescent	Wildtype	CC5	0.01085305
Cardiac crescent	Wildtype	CC6	0.00340408
Cardiac crescent	Wildtype	CC7	0.010921
Cardiac crescent	Wildtype	CC8	0.01019946
Cardiac crescent	*Nipbl*$^{+/-}$	CC9	0.15524194
Cardiac crescent	*Nipbl*$^{+/-}$	CC10	0.12248629
Cardiac crescent	*Nipbl*$^{+/-}$	CC11	0.08695652
Cardiac crescent	*Nipbl*$^{+/-}$	CC12	0.12486993
Cardiac crescent	*Nipbl*$^{+/-}$	CC13	0.11223629
Cardiac crescent	*Nipbl*$^{+/-}$	CC14	0.09711432
Cardiac crescent	*Nipbl*$^{+/-}$	CC15	0.07511408
Cardiac crescent	*Nipbl*$^{+/-}$	CC16	0.07357057

Table S11. Expression of *Pou5f1* in LB- and CC-stage embryos.

Genotype = genotype of embryo, Embryo = embryo identifier, Cell_Type = cell type in embryo, Pou5f1 = normalized transcript counts for *Pou5f1* in cell type.

**Stage**	**Genotype**	**Embryo**	***Pou5f1***
Late bud	Wildtype	LB1	8.3875296
Late bud	Wildtype	LB2	8.28772803
Late bud	Wildtype	LB3	8.98193548
Late bud	Wildtype	LB4	10.6762115
Late bud	Wildtype	LB5	7.02786378
Late bud	*Nipbl*$^{+/-}$	LB6	7.82741738
Late bud	*Nipbl*$^{+/-}$	LB7	12.1409774
Late bud	*Nipbl*$^{+/-}$	LB8	8.35897436
Late bud	*Nipbl*$^{+/-}$	LB9	10.3502488
Late bud	*Nipbl*$^{+/-}$	LB10	6.68201058
Late bud	*Nipbl*$^{+/-}$	LB11	7.03855826
Cardiac crescent	Wildtype	CC1	0.66165414
Cardiac crescent	Wildtype	CC2	1.05661232
Cardiac crescent	Wildtype	CC3	0.48193296
Cardiac crescent	Wildtype	CC4	0.83712785
Cardiac crescent	Wildtype	CC5	0.76383764
Cardiac crescent	Wildtype	CC6	0.43211854
Cardiac crescent	Wildtype	CC7	1.3815071
Cardiac crescent	Wildtype	CC8	0.96033545
Cardiac crescent	*Nipbl*$^{+/-}$	CC9	0.92271505
Cardiac crescent	*Nipbl*$^{+/-}$	CC10	0.74710542
Cardiac crescent	*Nipbl*$^{+/-}$	CC11	4.35652174
Cardiac crescent	*Nipbl*$^{+/-}$	CC12	1.9760666
Cardiac crescent	*Nipbl*$^{+/-}$	CC13	2.9164557
Cardiac crescent	*Nipbl*$^{+/-}$	CC14	0.91287458
Cardiac crescent	*Nipbl*$^{+/-}$	CC15	1.2965953
Cardiac crescent	*Nipbl*$^{+/-}$	CC16	1.39984006

Data S1. Differentially expressed genes among clusters of LB-stage WT embryos.

Differential gene expression analysis was performed between clusters of LB-stage WT embryos using the Mann-Whitney U test. P-values were adjusted for multiple hypotheses by Bonferroni Correction. Cluster = cluster number, Gene = gene, Pct_Expr_Cluster = percentage of cells in cluster expressing indicated gene, Pct_Expr_Other = percentage of cells in all other clusters expressing indicated gene, Avg_FC = average fold change in expression of indicated gene in cluster versus all other clusters, P_Val = p-value from Mann-Whitney U test, Bon_P_Val = Bonferroni corrected p-value, Germ_Layer = germ layer assigned to cluster.

Data S2. Differentially expressed genes among clusters of CC-stage WT embryos.

Differential gene expression analysis was performed between clusters of CC-stage WT embryos using the Mann-Whitney U test. P-values were adjusted for multiple hypotheses by Bonferroni Correction. Cluster = cluster number, Gene = gene, Pct_Expr_Cluster = percentage of cells in cluster expressing indicated gene, Pct_Expr_Other = percentage of cells in all other clusters expressing indicated gene, Avg_FC = average fold change in expression of indicated gene in cluster versus all other clusters, P_Val = p-value from Mann-Whitney U test, Bon_P_Val = Bonferroni corrected p-value, Germ_Layer = germ layer assigned to cluster.

Data S3. Differentially expressed genes among endodermal clusters of LB-stage WT embryos.

Differential gene expression analysis was performed between endodermal clusters of LB-stage WT embryos using the Mann-Whitney U test. P-values were adjusted for multiple hypotheses by Bonferroni Correction. Cluster = cluster number, Gene = gene, Pct_Expr_Cluster = percentage of cells in cluster expressing indicated gene, Pct_Expr_Other = percentage of cells in all other clusters expressing indicated gene, Avg_FC = average fold change in expression of indicated gene in cluster versus all other clusters, P_Val = p-value from Mann-Whitney U test, Bon_P_Val = Bonferroni corrected p-value, Cell_Type = cell type assigned to cluster, Transcription_Factor = Y, predicted by Animal Transcription Factor Database to be a transcription factor.

Data S4. Differentially expressed genes among endodermal clusters of CC-stage WT embryos.

Differential gene expression analysis was performed between endodermal clusters of CC-stage WT embryos using the Mann-Whitney U test. P-values were adjusted for multiple hypotheses by Bonferroni Correction. Cluster = cluster number, Gene = gene, Pct_Expr_Cluster = percentage of cells in cluster expressing indicated gene, Pct_Expr_Other = percentage of cells in all other clusters expressing indicated gene, Avg_FC = average fold change in expression of indicated gene in cluster versus all other clusters, P_Val = p-value from Mann-Whitney U test, Bon_P_Val = Bonferroni corrected p-value, Cell_Type = cell type assigned to cluster, Transcription_Factor = Y, predicted by Animal Transcription Factor Database to be a transcription factor.

Data S5. Differentially expressed genes among ectodermal clusters of LB-stage WT embryos.

Differential gene expression analysis was performed between ectodermal clusters of LB-stage WT embryos using the Mann-Whitney U test. P-values were adjusted for multiple hypotheses by Bonferroni Correction. Cluster = cluster number, Gene = gene, Pct_Expr_Cluster = percentage of cells in cluster expressing indicated gene, Pct_Expr_Other = percentage of cells in all other clusters expressing indicated gene, Avg_FC = average fold change in expression of indicated gene in cluster versus all other clusters, P_Val = p-value from Mann-Whitney U test, Bon_P_Val = Bonferroni corrected p-value, Cell_Type = cell type assigned to cluster, Transcription_Factor = Y, predicted by Animal Transcription Factor Database to be a transcription factor.

Data S6. Differentially expressed genes among ectodermal clusters of CC-stage WT embryos.

Differential gene expression analysis was performed between ectodermal clusters of CC-stage WT embryos using the Mann-Whitney U test. P-values were adjusted for multiple hypotheses by Bonferroni Correction. Cluster = cluster number, Gene = gene, Pct_Expr_Cluster = percentage of cells in cluster expressing indicated gene, Pct_Expr_Other = percentage of cells in all other clusters expressing indicated gene, Avg_FC = average fold change in expression of indicated gene in cluster versus all other clusters, P_Val = p-value from Mann-Whitney U test, Bon_P_Val = Bonferroni corrected p-value, Cell_Type = cell type assigned to cluster, Transcription_Factor = Y, predicted by Animal Transcription Factor Database to be a transcription factor.

Data S7. Differentially expressed genes among mesodermal clusters of LB-stage WT embryos.

Differential gene expression analysis was performed between mesodermal clusters of LB-stage WT embryos using the Mann-Whitney U test. P-values were adjusted for multiple hypotheses by Bonferroni Correction. Cluster = cluster number, Gene = gene, Pct_Expr_Cluster = percentage of cells in cluster expressing indicated gene, Pct_Expr_Other = percentage of cells in all other clusters expressing indicated gene, Avg_FC = average fold change in expression of indicated gene in cluster versus all other clusters, P_Val = p-value from Mann-Whitney U test, Bon_P_Val = Bonferroni corrected p-value, Cell_Type = cell type assigned to cluster, Transcription_Factor = Y, predicted by Animal Transcription Factor Database to be a transcription factor.

Data S8. Differentially expressed genes among mesodermal clusters of CC-stage WT embryos.

Differential gene expression analysis was performed between mesodermal clusters of CC-stage WT embryos using the Mann-Whitney U test. P-values were adjusted for multiple hypotheses by Bonferroni Correction. Cluster = cluster number, Gene = gene, Pct_Expr_Cluster = percentage of cells in cluster expressing indicated gene, Pct_Expr_Other = percentage of cells in all other clusters expressing indicated gene, Avg_FC = average fold change in expression of indicated gene in cluster versus all other clusters, P_Val = p-value from Mann-Whitney U test, Bon_P_Val = Bonferroni corrected p-value, Cell_Type = cell type assigned to cluster, Transcription_Factor = Y, predicted by Animal Transcription Factor Database to be a transcription factor.

Data S9. Numbers of cells per germ layer per LB-stage embryo.

Genotype = genotype of embryo, Embryo = embryo identifier, Germ_Layer = germ layer of embryo, No_Cells = number of cells in germ layer.

Data S10. Numbers of cells per mesodermal cell population per LB-stage embryo.

Genotype = genotype of embryo, Embryo = embryo identifier, Cell_Type = cell type of embryo, No_Cells = number of cells in cell type.

Data S11. Differentially expressed genes among clusters of LB-stage *Nipbl*$^{+/-}$ embryos.

Differential gene expression analysis was performed between clusters of LB-stage *Nipbl*$^{+/-}$ embryos using the Mann-Whitney U test. P-values were adjusted for multiple hypotheses by Bonferroni Correction. Cluster = cluster number, Gene = gene, Pct_Expr_Cluster = percentage of cells in cluster expressing indicated gene, Pct_Expr_Other = percentage of cells in all other clusters expressing indicated gene, Avg_FC = average fold change in expression of indicated gene in cluster versus all other clusters, P_Val = p-value from Mann-Whitney U test, Bon_P_Val = Bonferroni corrected p-value, Germ_Layer = germ layer assigned to cluster.

Data S12. Differentially expressed genes among mesodermal clusters of LB-stage *Nipbl*$^{+/-}$ embryos.

Differential gene expression analysis was performed between mesodermal clusters of LB-stage *Nipbl*$^{+/-}$ embryos using the Mann-Whitney U test. P-values were adjusted for multiple hypotheses by Bonferroni Correction. Cluster = cluster number, Gene = gene, Pct_Expr_Cluster = percentage of cells in cluster expressing indicated gene, Pct_Expr_Other = percentage of cells in all other clusters expressing indicated gene, Avg_FC = average fold change in expression of indicated gene in cluster versus all other clusters, P_Val = p-value from Mann-Whitney U test, Bon_P_Val = Bonferroni corrected p-value, Cell_Type = cell type assigned to cluster.

Data S13. Numbers of cells per germ layer per LB-stage embryo following projection of WT cells onto *Nipbl*$^{+/-}$ germ layers.

Genotype = genotype of embryo, Embryo = embryo identifier, Germ_Layer = germ layer of embryo, No_Cells = number of cells in germ layer.

Data S14. Numbers of cells per mesodermal cell population per LB-stage embryo following projection of WT cells onto *Nipbl*$^{+/-}$ mesodermal cell populations.

Genotype = genotype of embryo, Embryo = embryo identifier, Cell_Type = cell type of embryo, No_Cells = number of cells in cell type.

Data S15. Differentially expressed genes among clusters of EB-stage WT embryos.

Differential gene expression analysis was performed between clusters of EB-stage WT embryos using the Mann-Whitney U test. P-values were adjusted for multiple hypotheses by Bonferroni Correction. Cluster = cluster number, Gene = gene, Pct_Expr_Cluster = percentage of cells in cluster expressing indicated gene, Pct_Expr_Other = percentage of cells in all other clusters expressing indicated gene, Avg_FC = average fold change in expression of indicated gene in cluster versus all other clusters, P_Val = p-value from Mann-Whitney U test, Bon_P_Val = Bonferroni corrected p-value, Germ_Layer = germ layer assigned to cluster.

Data S16. Differentially expressed genes among clusters of EHF-stage WT embryos.

Differential gene expression analysis was performed between clusters of EHF-stage WT embryos using the Mann-Whitney U test. P-values were adjusted for multiple hypotheses by Bonferroni Correction. Cluster = cluster number, Gene = gene, Pct_Expr_Cluster = percentage of cells in cluster expressing indicated gene, Pct_Expr_Other = percentage of cells in all other clusters expressing indicated gene, Avg_FC = average fold change in expression of indicated gene in cluster versus all other clusters, P_Val = p-value from Mann-Whitney U test, Bon_P_Val = Bonferroni corrected p-value, Germ_Layer = germ layer assigned to cluster.

Data S17. Pseudotime values of cells from EB-, LB-, and CC-stage embryos.

Pseudotime of cells from EB-, LB-, and CC-stage embryos were calculated using URD. Stage = stage of embryo, Genotype = genotype of embryo, Embryo = identifier of embryo, Cell_Barcode = barcode identifying cell, Cluster = cluster number, Germ_Layer = germ layer assigned to cell, Pseudotime = pseudotime value of cell.

Data S18. RNA velocity vectors of mesoderm cells from LB-stage WT embryos.

scVelo was used to calculate the RNA velocities of mesoderm cells from LB-stage WT embryos. Embryo = embryo identifier, Cell_Barcode = barcode identifying cell, UMAP_1 = first coordinate in UMAP, UMAP_2 = second coordinate in UMAP, Cluster = cluster number, Cell_Type = cell type assigned to cell, Velocity_1 = first coordinate of RNA velocity vector, Velocity_2 = second coordinate of RNA velocity vector.

Data S19. RNA velocity vectors of mesoderm cells from LB-stage *Nipbl*$^{+/-}$ embryos.

scVelo was used to calculate the RNA velocities of mesoderm cells from LB-stage *Nipbl*$^{+/-}$ embryos. Embryo = embryo identifier, Cell_Barcode = barcode identifying cell, UMAP_1 = first coordinate in UMAP, UMAP_2 = second coordinate in UMAP, Cluster = cluster number, Cell_Type = cell type assigned to cell, Velocity_1 = first coordinate of RNA velocity vector, Velocity_2 = second coordinate of RNA velocity vector.

Data S20. Fate probabilities of mesoderm cells into first heart field, second heart field, and paraxial mesoderm fates from LB-stage WT embryos.

CellRank was used to calculate the fate probabilities of mesoderm cells into first heart field, second heart field, and paraxial mesoderm fates from LB-stage WT embryos. Embryo = embryo identifier, Cell_Barcode = barcode identifying cell, UMAP_1 = first coordinate in UMAP, UMAP_2 = second coordinate in UMAP, Cluster = cluster number, Cell_Type = cell type assigned to cell, FHF_Prob = first heart field fate probability, SHF_Prob = second heart field fate probability, PM_Prob = paraxial mesoderm fate probability.

Data S21. Fate probabilities of mesoderm cells into first heart field, second heart field, and paraxial mesoderm fates from LB-stage *Nipbl*$^{+/-}$ embryos.

CellRank was used to calculate the fate probabilities of mesoderm cells into first heart field, second heart field, and paraxial mesoderm fates from LB-stage *Nipbl*$^{+/-}$ embryos. Embryo = embryo identifier, Cell_Barcode = barcode identifying cell, UMAP_1 = first coordinate in UMAP, UMAP_2 = second coordinate in UMAP, Cluster = cluster number, Cell_Type = cell type assigned to cell, FHF_Prob = first heart field fate probability, SHF_Prob = second heart field fate probability, PM_Prob = paraxial mesoderm fate probability.

Data S22. Differentially expressed genes from Reactome, Hallmark, and Langemeijer Apoptosis Gene Set between germ layers of LB-stage WT and *Nipbl*$^{+/-}$ embryos.

Differential gene expression analysis was performed using genes from the Reactome, Hallmark, and Langemeijer Apoptosis Gene Set between the germ layers of LB-stage WT and *Nipbl*$^{+/-}$ embryos using the Mann-Whitney U test. P-values were adjusted for multiple hypotheses by Bonferroni Correction. Gene = gene, Avg_Expr_Wildtype = average expression of indicated gene in Wildtype cells, Avg_Expr_Nipbl+/- = average expression of indicated gene in *Nipbl*$^{+/-}$ cells, SE_Wildtype = standard error of average expression in Wildtype cells, SE_Nipbl+/- = standard error of average expression in *Nipbl*$^{+/-}$ cells, Avg_FC = average fold change in expression of indicated gene in *Nipbl*$^{+/-}$ cells versus WT cells, P_Val = p-value from Mann-Whitney U test, Bon_P_Val = Bonferroni corrected p-value.

Data S23. Differentially expressed genes from meta-PCNA Gene Set between germ layers of LB-stage WT and *Nipbl*$^{+/-}$ embryos.

Differential gene expression analysis was performed using genes from the meta-PCNA Gene Set between the germ layers of LB-stage WT and *Nipbl*$^{+/-}$ embryos using the Mann-Whitney U test. P-values were adjusted for multiple hypotheses by Bonferroni Correction. Gene = gene, Avg_Expr_Wildtype = average expression of indicated gene in Wildtype cells, Avg_Expr_Nipbl+/- = average expression of indicated gene in *Nipbl*$^{+/-}$ cells, SE_Wildtype = standard error of average expression in Wildtype cells, SE_Nipbl+/- = standard error of average expression in *Nipbl*$^{+/-}$ cells, Avg_FC = average fold change in expression of indicated gene in *Nipbl*$^{+/-}$ cells versus WT cells, P_Val = p-value from Mann-Whitney U test, Bon_P_Val = Bonferroni corrected p-value.

Data S24. Cell cycle scores and phases of cells in germ layers of LB-stage WT and *Nipbl*$^{+/-}$ embryos.

Seurat was used to score and assign cells from all germ layers of LB-stage WT and *Nipbl*$^{+/-}$ embryos into either G1, S, or G2/M phase on the basis of the expression of markers of S and G2/M phase. Expression of these marker sets are considered anticorrelated and when cells express neither, they are considered to be in G1 phase. Dimensional reduction was performed using principal component analysis on S phase and G2/M markers. Genotype = genotype of embryo, Embryo = identifier of embryo, Cell_Barcode = barcode identifying cell, PC_1 = principal component 1, PC_2 = principal component 2, Score_S_Phase = score for S phase markers, Score_G2M_Phase = score for G2/M phase markers, Phase = assigned cell cycle phase.

Data S25. First heart field drivers and anti-drivers predicted by CellRank.

Gene = gene, Slope = change in expression of gene over change in absorption probability towards FHF fate in WT cells in Euclidean space, Spearman_Coefficient = correlation coefficient from Spearman's Rank Correlation Test between expression of gene and absorption probability, P_Val = p-value from Spearman's Rank Correlation Test, Ben_Hoch_P_Val = Benjamini-Hochberg corrected p-value, Avg_Expr_WT = average expression of gene in WT cells, Transcription_Factor = Y, predicted by Animal Transcription Factor Database to be a transcription factor.

Data S26. Paraxial mesoderm drivers and anti-drivers predicted by CellRank.

Gene = gene, Slope = change in expression of gene over change in absorption probability towards PM fate in WT cells in Euclidean space, Spearman_Coefficient = correlation coefficient from Spearman's Rank Correlation Test between expression of gene and absorption probability, P_Val = p-value from Spearman's Rank Correlation Test, Ben_Hoch_P_Val = Benjamini-Hochberg corrected p-value, Avg_Expr_WT = average expression of gene in WT cells, Transcription_Factor = Y, predicted by Animal Transcription Factor Database to be a transcription factor.

Data S27. Differentially expressed genes between first heart field lineage of LB-stage WT and *Nipbl*$^{+/-}$ embryos.

Differential gene expression analysis was performed between FHF lineage of LB-stage WT and *Nipbl*$^{+/-}$ embryos using the Mann-Whitney U test. P-values were adjusted for multiple hypotheses by Bonferroni Correction. Gene = gene, Avg_FC = average fold change in expression of indicated gene in *Nipbl*$^{+/-}$ cells versus WT cells, P_Val = p-value from Mann-Whitney U test, Bon_P_Val = Bonferroni corrected p-value, Transcription_Factor = Y, predicted by Animal Transcription Factor Database to be a transcription factor.

Data S28. Differentially expressed genes between paraxial mesoderm lineage of LB-stage WT and *Nipbl*$^{+/-}$ embryos.

Differential gene expression analysis was performed between PM lineage of LB-stage WT and *Nipbl*$^{+/-}$ embryos using the Mann-Whitney U test. P-values were adjusted for multiple hypotheses by Bonferroni Correction. Gene = gene, Avg_FC = average fold change in expression of indicated gene in *Nipbl*$^{+/-}$ cells versus WT cells, P_Val = p-value from Mann-Whitney U test, Bon_P_Val = Bonferroni corrected p-value, Transcription_Factor = Y, predicted by Animal Transcription Factor Database to be a transcription factor.

Data S29. Differentially expressed genes between mesoderms of LB-stage WT and *Nipbl*$^{+/-}$ embryos.

Differential gene expression analysis was performed between mesoderms of LB-stage WT and *Nipbl*$^{+/-}$ embryos using the Mann-Whitney U test. P-values were adjusted for multiple hypotheses by Bonferroni Correction. Gene = gene, Avg_Expr_Wildtype = average expression of indicated gene in Wildtype cells, Avg_Expr_Nipbl+/- = average expression of indicated gene in *Nipbl*$^{+/-}$ cells, SE_Wildtype = standard error of average expression in Wildtype cells, SE_Nipbl+/- = standard error of average expression in *Nipbl*$^{+/-}$ cells, Avg_FC = average fold change in expression of indicated gene in *Nipbl*$^{+/-}$ cells versus WT cells, P_Val = p-value from Mann-Whitney U test, Bon_P_Val = Bonferroni corrected p-value, STRING_Interactor = Y, predicted by STRING to interact with other genes in a network, Nanog_Target = predicted by Fig. 8G to be a target of *Nanog*.

Data S30. Differentially expressed genes between ectoderms of LB-stage WT and *Nipbl*$^{+/-}$ embryos.

Differential gene expression analysis was performed between ectoderms of LB-stage WT and *Nipbl*$^{+/-}$ embryos using the Mann-Whitney U test. P-values were adjusted for multiple hypotheses by Bonferroni Correction. Gene = gene, Avg_Expr_Wildtype = average expression of indicated gene in Wildtype cells, Avg_Expr_Nipbl+/- = average expression of indicated gene in *Nipbl*$^{+/-}$ cells, SE_Wildtype = standard error of average expression in Wildtype cells, SE_Nipbl+/- = standard error of average expression in *Nipbl*$^{+/-}$ cells, Avg_FC = average fold change in expression of indicated gene in *Nipbl*$^{+/-}$ cells versus WT cells, P_Val = p-value from Mann-Whitney U test, Bon_P_Val = Bonferroni corrected p-value, STRING_Interactor = Y, predicted by STRING to interact with other genes in a network, Nanog_Target = predicted by Fig. 8G to be a target of *Nanog*.

Data S31. Differentially expressed genes between endoderms of LB-stage WT and *Nipbl*$^{+/-}$ embryos.

Differential gene expression analysis was performed between endoderms of LB-stage WT and *Nipbl*$^{+/-}$ embryos using the Mann-Whitney U test. P-values were adjusted for multiple hypotheses by Bonferroni Correction. Gene = gene, Avg_Expr_Wildtype = average expression of indicated gene in Wildtype cells, Avg_Expr_Nipbl+/- = average expression of indicated gene in *Nipbl*$^{+/-}$ cells, SE_Wildtype = standard error of average expression in Wildtype cells, SE_Nipbl+/- = standard error of average expression in *Nipbl*$^{+/-}$ cells, Avg_FC = average fold change in expression of indicated gene in *Nipbl*$^{+/-}$ cells versus WT cells, P_Val = p-value from Mann-Whitney U test, Bon_P_Val = Bonferroni corrected p-value, STRING_Interactor = Y, predicted by STRING to interact with other genes in a network, Nanog_Target = predicted by Fig. 8G to be a target of *Nanog*.

Data S32. Differentially expressed genes between mesoderms of LB-stage WT and *Nipbl*$^{+/-}$ embryos following projection of WT cells onto *Nipbl*$^{+/-}$ germ layers.

Differential gene expression analysis was performed between mesoderms of LB-stage WT and *Nipbl*$^{+/-}$ embryos using the Mann-Whitney U test. P-values were adjusted for multiple hypotheses by Bonferroni Correction. Gene = gene, Avg_Expr_Wildtype = average expression of indicated gene in Wildtype cells, Avg_Expr_Nipbl+/- = average expression of indicated gene in *Nipbl*$^{+/-}$ cells, SE_Wildtype = standard error of average expression in Wildtype cells, SE_Nipbl+/- = standard error of average expression in *Nipbl*$^{+/-}$ cells, Avg_FC = average fold change in expression of indicated gene in *Nipbl*$^{+/-}$ cells versus WT cells, P_Val = p-value from Mann-Whitney U test, Bon_P_Val = Bonferroni corrected p-value.

Data S33. Differentially expressed genes between ectoderms of LB-stage WT and *Nipbl*$^{+/-}$ embryos following projection of WT cells onto *Nipbl*$^{+/-}$ germ layers.

Differential gene expression analysis was performed between ectoderms of LB-stage WT and *Nipbl*$^{+/-}$ embryos using the Mann-Whitney U test. P-values were adjusted for multiple hypotheses by Bonferroni Correction. Gene = gene, Avg_Expr_Wildtype = average expression of indicated gene in Wildtype cells, Avg_Expr_Nipbl+/- = average expression of indicated gene in *Nipbl*$^{+/-}$ cells, SE_Wildtype = standard error of average expression in Wildtype cells, SE_Nipbl+/- = standard error of average expression in *Nipbl*$^{+/-}$ cells, Avg_FC = average fold change in expression of indicated gene in *Nipbl*$^{+/-}$ cells versus WT cells, P_Val = p-value from Mann-Whitney U test, Bon_P_Val = Bonferroni corrected p-value.

Data S34. Differentially expressed genes between endoderms of LB-stage WT and *Nipbl*$^{+/-}$ embryos following projection of WT cells onto *Nipbl*$^{+/-}$ germ layers.

Differential gene expression analysis was performed between endoderms of LB-stage WT and *Nipbl*$^{+/-}$ embryos using the Mann-Whitney U test. P-values were adjusted for multiple hypotheses by Bonferroni Correction. Gene = gene, Avg_Expr_Wildtype = average expression of indicated gene in Wildtype cells, Avg_Expr_Nipbl+/- = average expression of indicated gene in *Nipbl*$^{+/-}$ cells, SE_Wildtype = standard error of average expression in Wildtype cells, SE_Nipbl+/- = standard error of average expression in *Nipbl*$^{+/-}$ cells, Avg_FC = average fold change in expression of indicated gene in *Nipbl*$^{+/-}$ cells versus WT cells, P_Val = p-value from Mann-Whitney U test, Bon_P_Val = Bonferroni corrected p-value.

Data S35. Network of gene interactions predicted by STRING for genes differentially expressed more than two-fold up or down in germ layers of LB-stage *Nipbl*$^{+/-}$ embryos than WT embryos.

For each germ layer, genes differentially expressed more than two-fold up or down in Nipbl+/- embryos relative to WT embryos were inputted into STRING. STRING outputted networks of predicted gene interactions.

Data S36. Expression of *Nanog* in mesodermal cell populations of LB-stage embryos.

Genotype = genotype of embryo, Embryo = embryo identifier, Cell_Type = cell type in embryo, Nanog = normalized transcript counts for *Nanog* in cell type.

Data S37. Expression of *Pou5f1* in mesodermal cell populations of LB-stage embryos.

Genotype = genotype of embryo, Embryo = embryo identifier, Cell_Type = cell type in embryo, Pou5f1 = normalized transcript counts for *Pou5f1* in cell type.

Data S38. qRT-PCR results for *Nipbl*, *Nanog*, and *Pou5f1* in *Nipbl$^{FLEX/+}$* and *Nipbl$^{Flrt/+}$* ES cells.

RNA extracted from 9 clones of *Nipbl$^{FLEX/+}$* (WT) and *Nipbl$^{Flrt/+}$* (*Nipbl$^{+/-}$*) ES cells. cDNA was synthesized from extracted RNA. qRT-PCR was performed for *Nipbl*, *Nanog*, and *Pou5f1* for each clone in technical replicates of three, with *Rpl4* as the housekeeping gene.

Data S39. Differentially expressed genes between whole LB-stage WT and *Nipbl$^{+/-}$* embryos.

Differential gene expression analysis was performed between whole LB-stage WT and *Nipbl$^{+/-}$* embryos using the Mann-Whitney U test. P-values were adjusted for multiple hypotheses by Bonferroni Correction. Gene = gene, Avg_Expr_Wildtype = average expression of indicated gene in Wildtype cells, Avg_Expr_Nipbl+/- = average expression of indicated gene in *Nipbl$^{+/-}$* cells, SE_Wildtype = standard error of average expression in Wildtype cells, SE_Nipbl+/- = standard error of average expression in *Nipbl$^{+/-}$* cells, Avg_FC = average fold change in expression of indicated gene in *Nipbl$^{+/-}$* cells versus WT cells, P_Val = p-value from Mann-Whitney U test, Bon_P_Val = Bonferroni corrected p-value, Nanog_Target = predicted by Fig. 8A to be a target of *Nanog*.

Data S40. Differentially expressed genes between whole CC-stage WT and *Nipbl$^{+/-}$* embryos.

Differential gene expression analysis was performed between whole CC-stage WT and *Nipbl$^{+/-}$* embryos using the Mann-Whitney U test. P-values were adjusted for multiple hypotheses by Bonferroni Correction. Gene = gene, Avg_Expr_Wildtype = average expression of indicated gene in Wildtype cells, Avg_Expr_Nipbl+/- = average expression of indicated gene in *Nipbl$^{+/-}$* cells, SE_Wildtype = standard error of average expression in Wildtype cells, SE_Nipbl+/- = standard error of average expression in *Nipbl$^{+/-}$* cells, Avg_FC = average fold change in expression of indicated gene in *Nipbl$^{+/-}$* cells versus WT cells, P_Val = p-value from Mann-Whitney U test, Bon_P_Val = Bonferroni corrected p-value, Nanog_Target = predicted by Fig. 8C to be a target of *Nanog*.

Data S41. Differentially expressed genes between mesoderms of CC-stage WT and *Nipbl$^{+/-}$* embryos.

Differential gene expression analysis was performed between mesoderms of CC-stage WT and *Nipbl$^{+/-}$* embryos using the Mann-Whitney U test. P-values were adjusted for multiple hypotheses by Bonferroni Correction. Gene = gene, Avg_Expr_Wildtype = average expression of indicated gene in Wildtype cells, Avg_Expr_Nipbl+/- = average expression of indicated gene in *Nipbl$^{+/-}$* cells, SE_Wildtype = standard error of average expression in Wildtype cells, SE_Nipbl+/- = standard error of average expression in *Nipbl$^{+/-}$* cells, Avg_FC = average fold change in expression of indicated gene in *Nipbl$^{+/-}$* cells versus WT cells, P_Val = p-value from Mann-Whitney U test, Bon_P_Val = Bonferroni corrected p-value, Nanog_Target = predicted by Fig. 8H to be a target of *Nanog*.

Data S42. Differentially expressed genes between ectoderms of CC-stage WT and *Nipbl*$^{+/-}$ embryos.

Differential gene expression analysis was performed between ectoderms of CC-stage WT and *Nipbl*$^{+/-}$ embryos using the Mann-Whitney U test. P-values were adjusted for multiple hypotheses by Bonferroni Correction. Gene = gene, Avg_Expr_Wildtype = average expression of indicated gene in Wildtype cells, Avg_Expr_Nipbl+/- = average expression of indicated gene in *Nipbl*$^{+/-}$ cells, SE_Wildtype = standard error of average expression in Wildtype cells, SE_Nipbl+/- = standard error of average expression in *Nipbl*$^{+/-}$ cells, Avg_FC = average fold change in expression of indicated gene in *Nipbl*$^{+/-}$ cells versus WT cells, P_Val = p-value from Mann-Whitney U test, Bon_P_Val = Bonferroni corrected p-value, Nanog_Target = predicted by Fig. 8H to be a target of *Nanog*.

Data S43. Differentially expressed genes between endoderms of CC-stage WT and *Nipbl*$^{+/-}$ embryos.

Differential gene expression analysis was performed between endoderms of CC-stage WT and *Nipbl*$^{+/-}$ embryos using the Mann-Whitney U test. P-values were adjusted for multiple hypotheses by Bonferroni Correction. Gene = gene, Avg_Expr_Wildtype = average expression of indicated gene in Wildtype cells, Avg_Expr_Nipbl+/- = average expression of indicated gene in *Nipbl*$^{+/-}$ cells, SE_Wildtype = standard error of average expression in Wildtype cells, SE_Nipbl+/- = standard error of average expression in *Nipbl*$^{+/-}$ cells, Avg_FC = average fold change in expression of indicated gene in *Nipbl*$^{+/-}$ cells versus WT cells, P_Val = p-value from Mann-Whitney U test, Bon_P_Val = Bonferroni corrected p-value, Nanog_Target = predicted by Fig. 8H to be a target of *Nanog*.

Data S44. Expression of *Hox* genes in mesoderms of LB- and CC-stage embryos.

Stage = stage of embryo, Genotype = genotype of embryo, Embryo = embryo identifier, Gene = *Hox* gene, Norm_Transcripts = normalized transcript counts for indicated *Hox* gene.

Data S45. Expression of *Hox* genes in ectoderms of LB- and CC-stage WT embryos.

Stage = stage of embryo, Genotype = genotype of embryo, Embryo = embryo identifier, Gene = *Hox* gene, Norm_Transcripts = normalized transcript counts for indicated *Hox* gene.
